# Supplementary figures and images for: An in vitro tumor recurrence model based on platinum-resistant colon cancer cells as a research tool for studying cancer cell dormancy
Source: PLoS One. 2025 Oct 8;20(10):e0333671. doi: 10.1371/journal.pone.0333671 (PMC12507233; doi:10.1371/journal.pone.0333671)

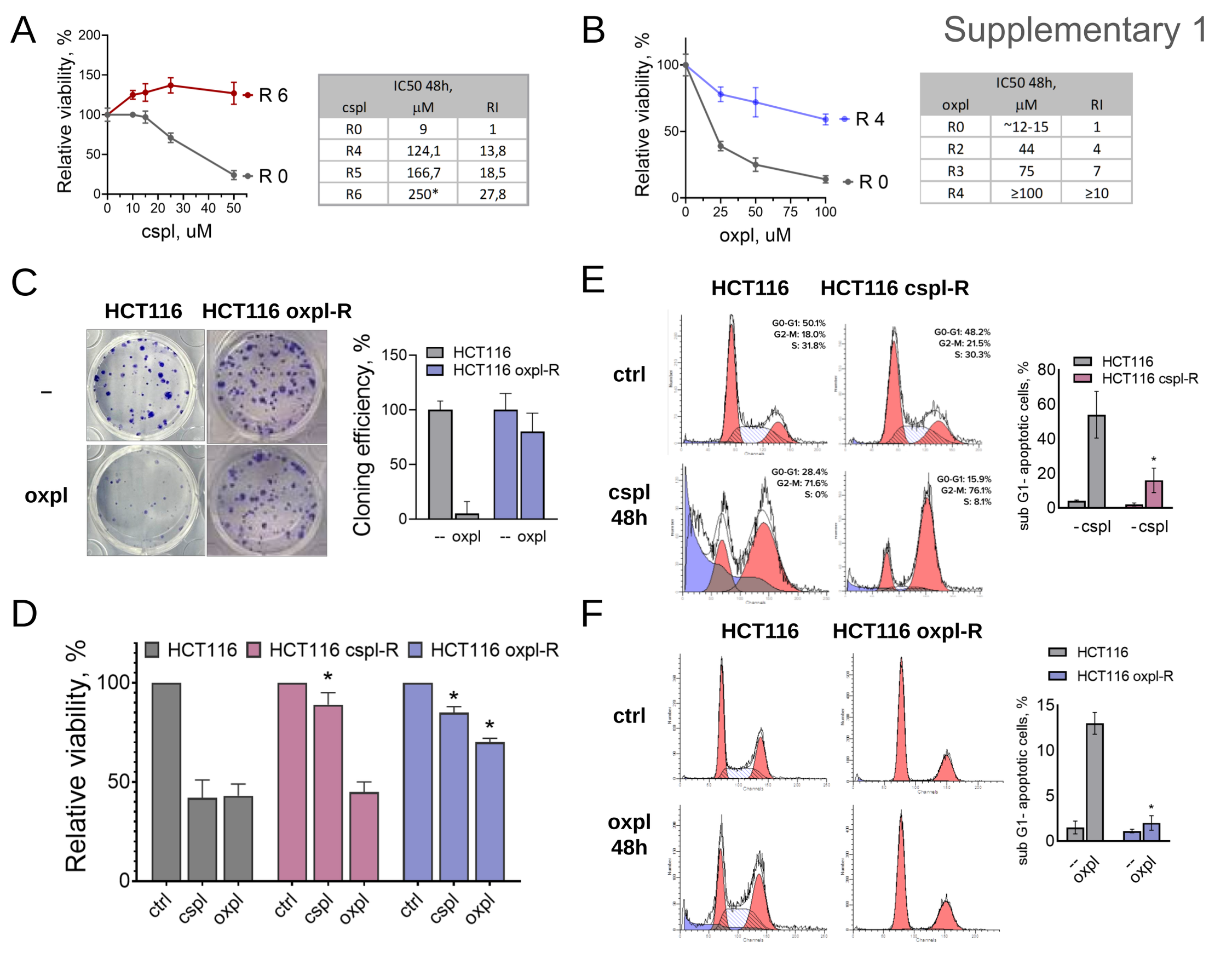

Supplement: S1 Fig — (A,B) Dose-dependent curves and IC50 tables for (A) cisplatin-resistant (cspl-R), and (B) oxaliplatin-resistant (oxpl-R) HCT116 cells. (C) Colony formation assay comparing oxaliplatin-sensitive HCT116 cells and oxaliplatin-resistant (oxpl-R) cells after 24 h treatment with 5 µM oxaliplatin (day 12). (D) MTT assay showing cross-resistance to platinum drugs in three cell lines: parental HCT116 cells, cisplatin-resistant HCT116 cells (HCT116 cspl-R), and oxaliplatin-resistant HCT116 cells (HCT116 oxpl-R). (E,F) Flow cytometry analysis of DNA content distribution (PI staining) in cisplatin-resistant (HCT116 cspl-R) (E) and oxaliplatin-resistant (HCT116 oxpl-R) (F) cells compared to parental HCT116 cells. Bars depict the percentage of cells with subdiploid DNA. All data represent biological triplicate experiments, mean ± SEM. (TIF) [file pone.0333671.s001.tif]

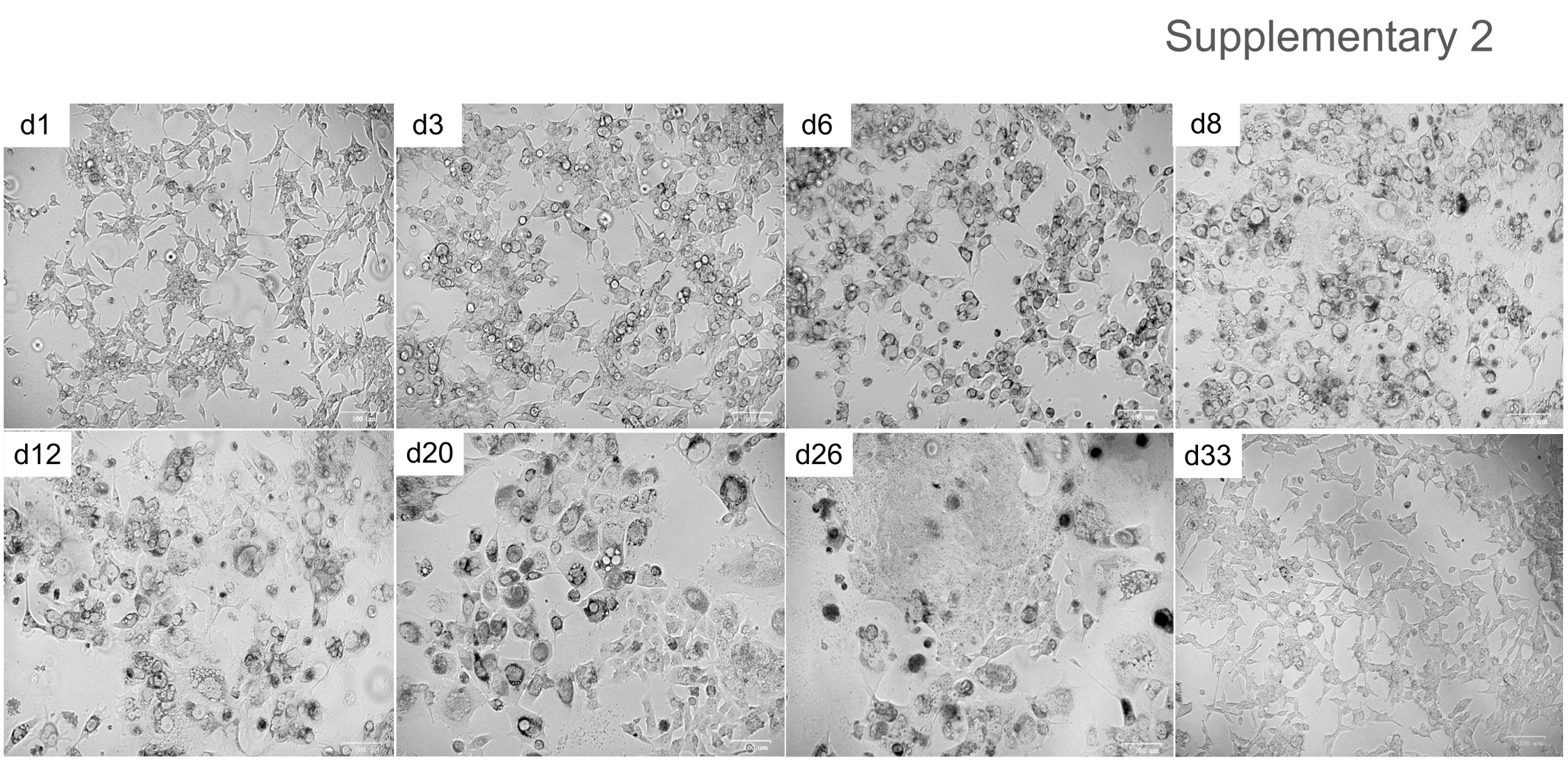

Supplement: S2 Fig — The scale bars represent 100 μm. (TIF) [file pone.0333671.s002.tif]

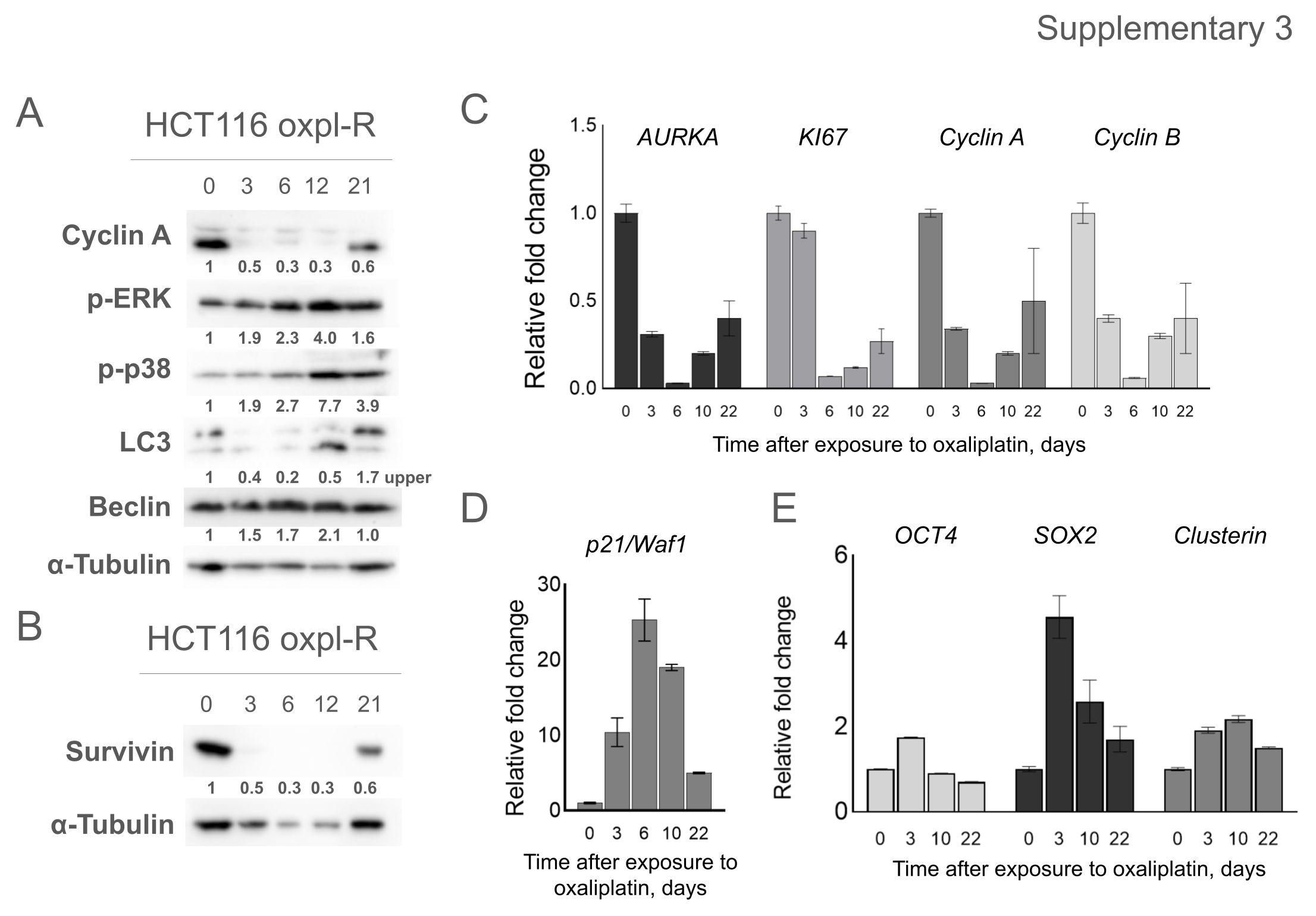

Supplement: S3 Fig — Immunoblot analysis of Cyclin A, p-ERK, p-p38, LC3, Beclin (A), and Survivin (B) protein levels (α-Tubulin loading control) in oxaliplatin-resistant HCT116 cells (HCT116 oxpl-R) after oxaliplatin exposure (days 0–21). Numbers indicate normalized densitometry values relative to day 0. Normalized expression of proliferation-related genes (AURKA, KI67, Cyclin A, Cycline B, p21/Waf1) (C,D) and stemness-related genes (OCT4, SOX2, NANOG) (E) after oxaliplatin exposure (days 0–22), relative to day 0. Expression of the GAPDH gene served as the endogenous control. Data represent biological triplicate experiments and are displayed as mean ± SEM. (TIF) [file pone.0333671.s003.tif]
