# Supplementary material for: An in vitro tumor recurrence model based on platinum-resistant colon cancer cells as a research tool for studying cancer cell dormancy
Source: PLoS One. 2025 Oct 8;20(10):e0333671. doi: 10.1371/journal.pone.0333671 (PMC12507233; doi:10.1371/journal.pone.0333671)
Supplement: S1 File — (PDF) [file pone.0333671.s004.pdf]

Raw blot images

Raw blot images

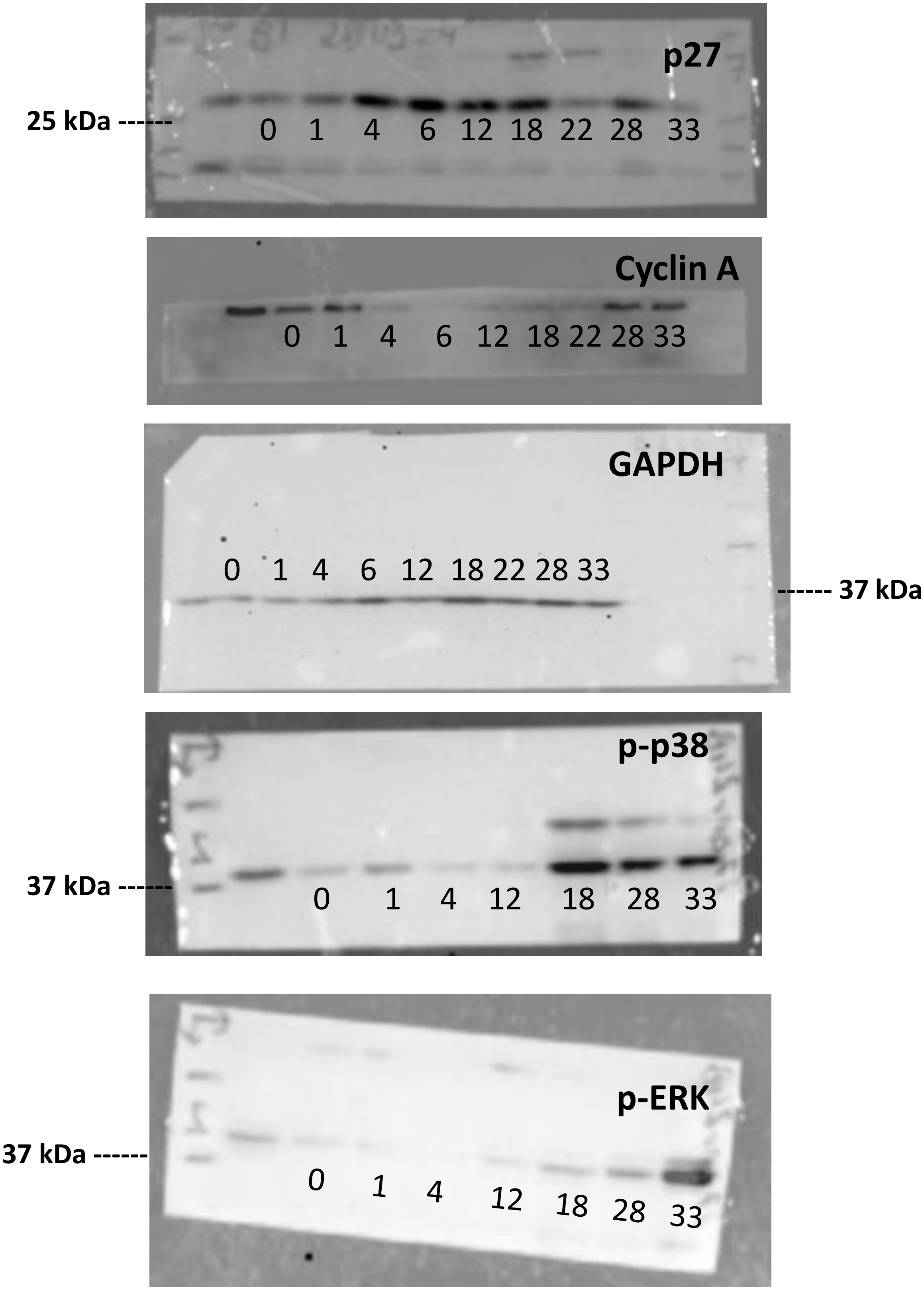

Raw blot images

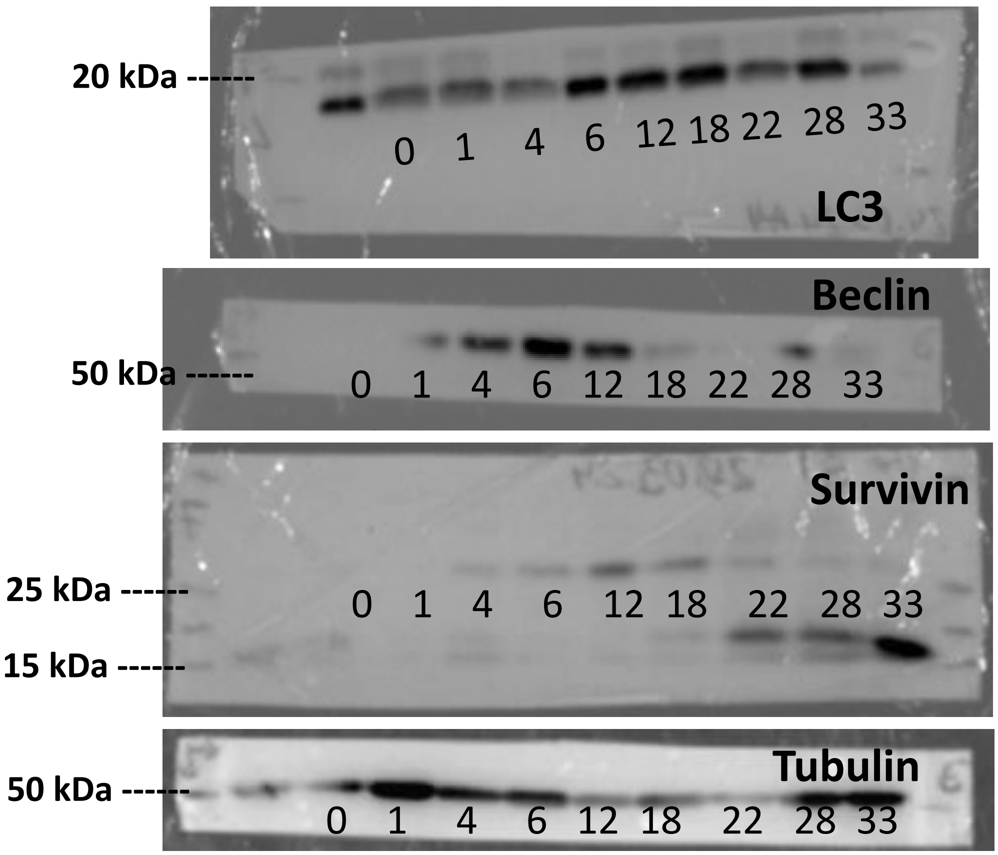

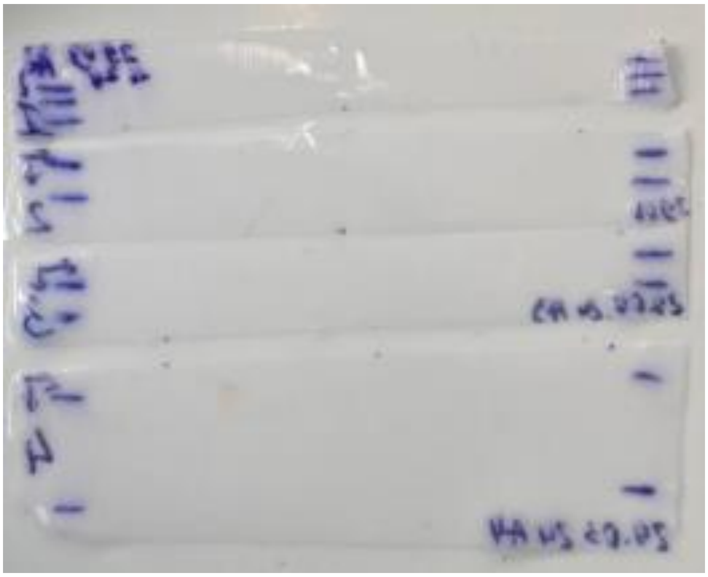

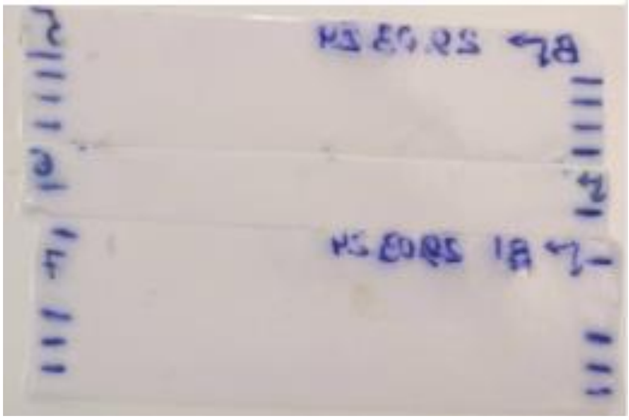

Raw microscopic images

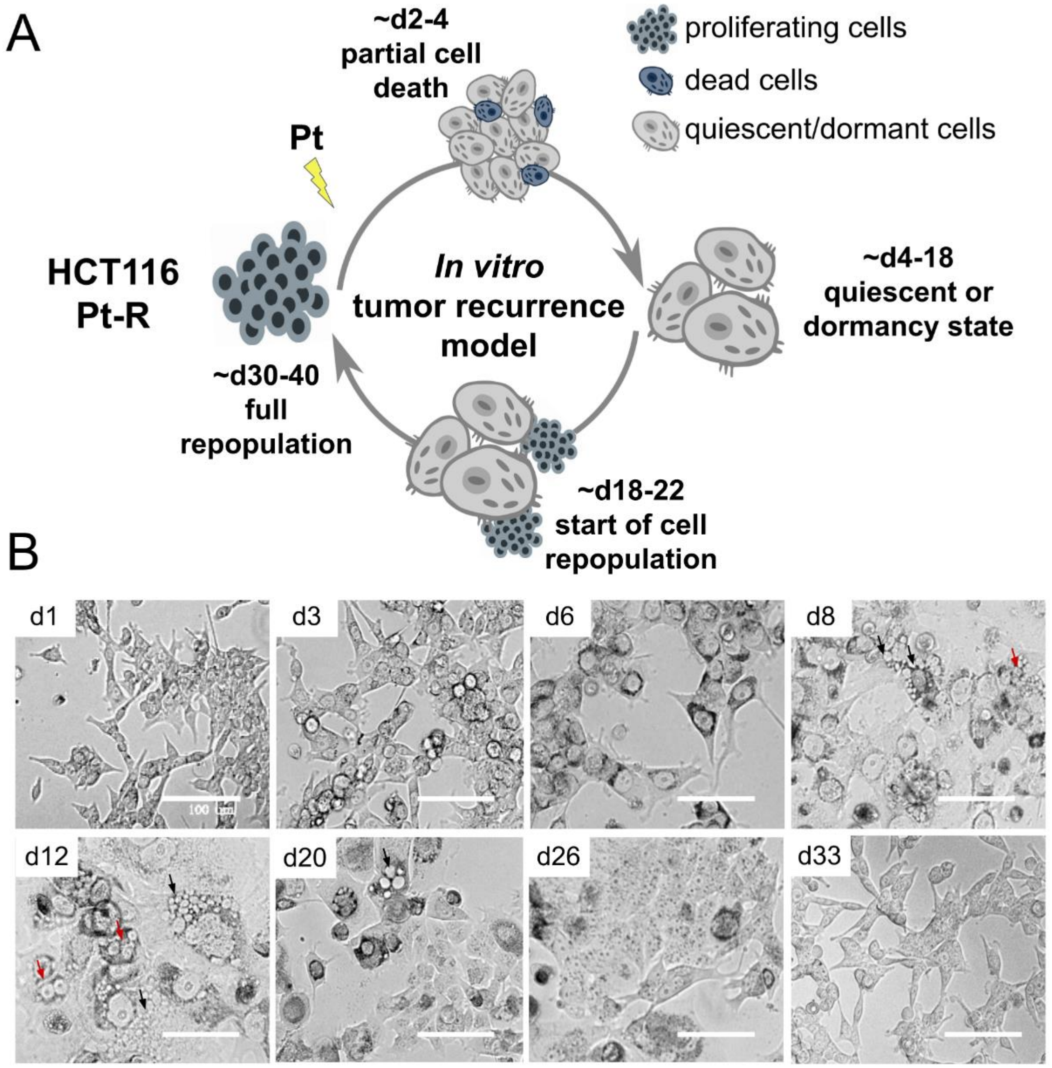

Supplementary 2

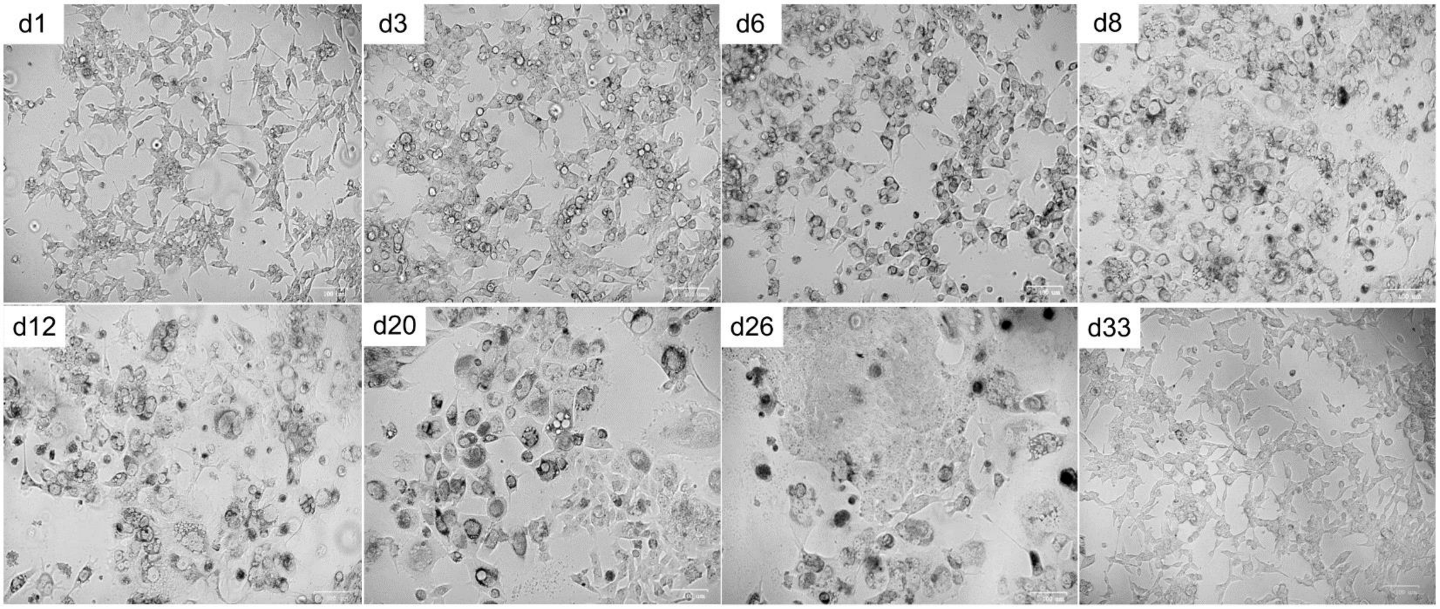

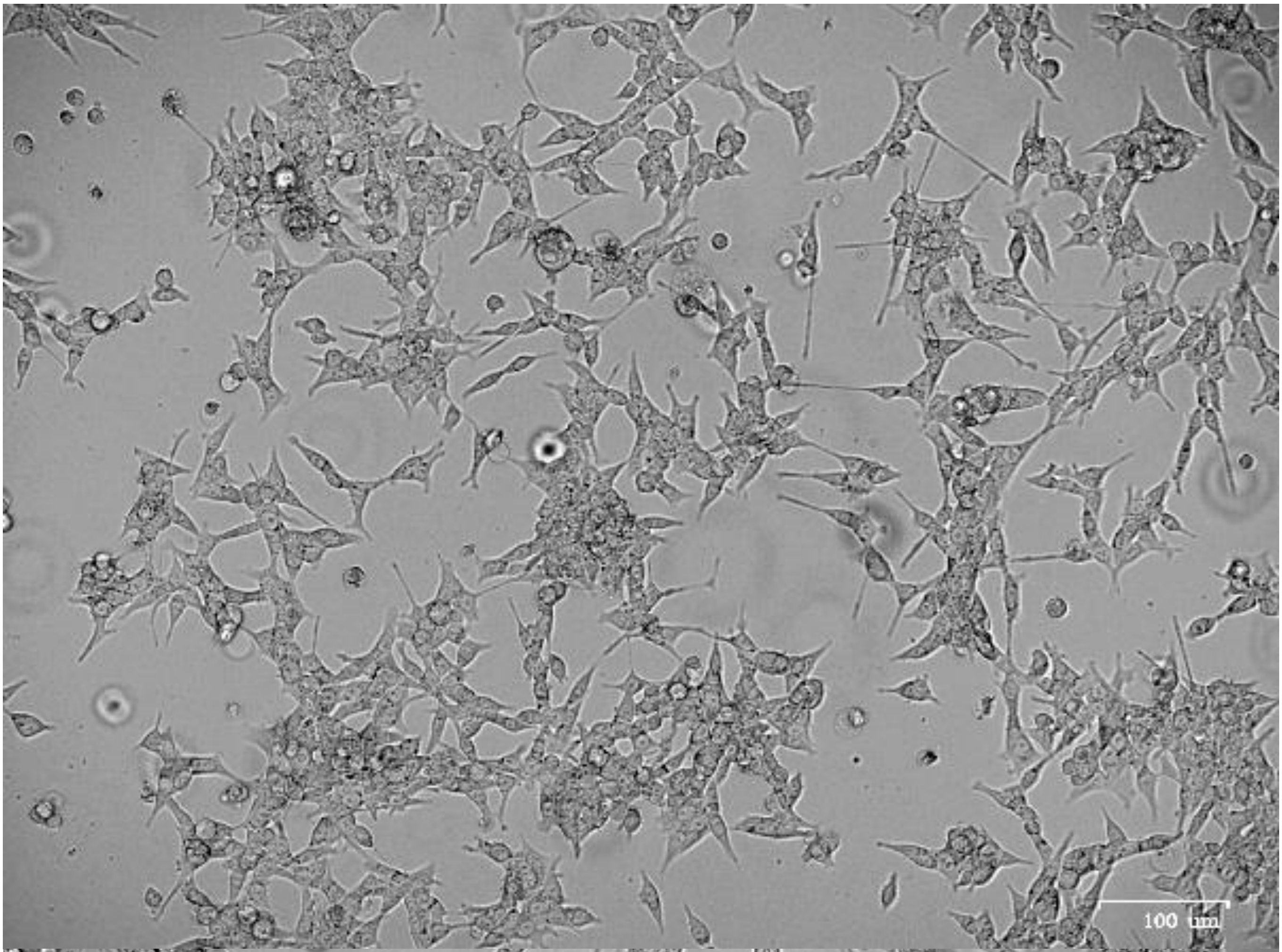
100 um

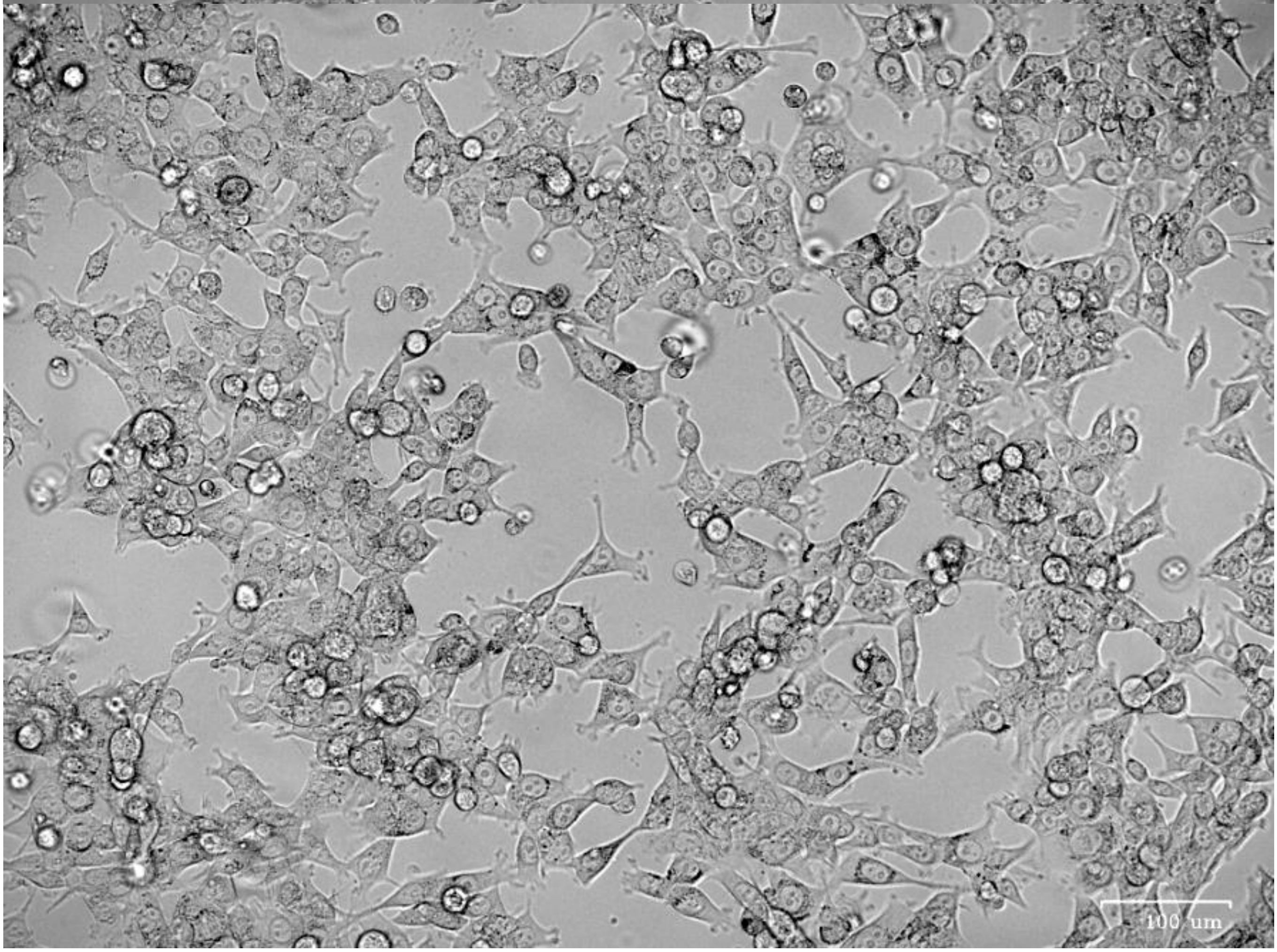
100 um

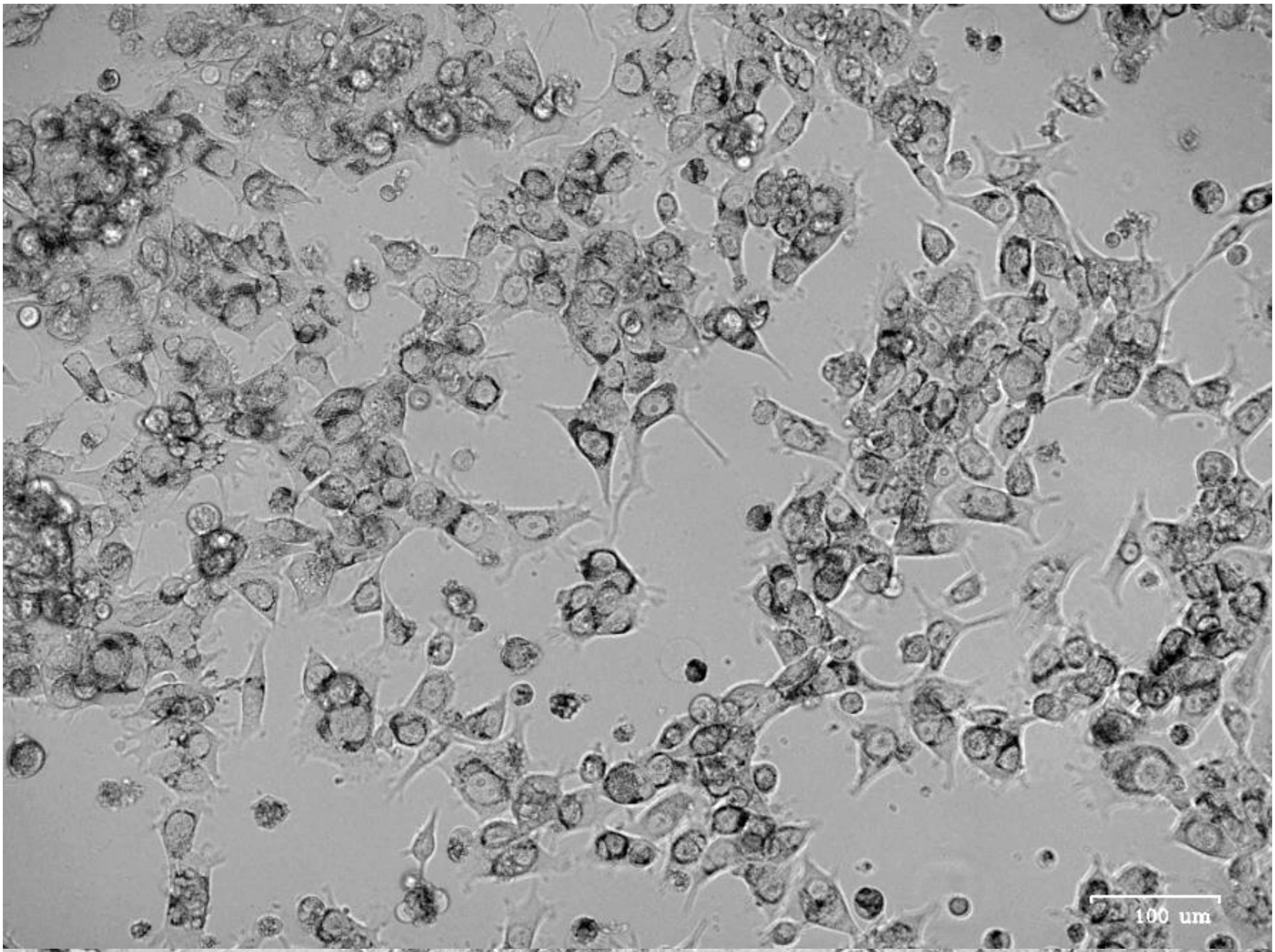
100 um
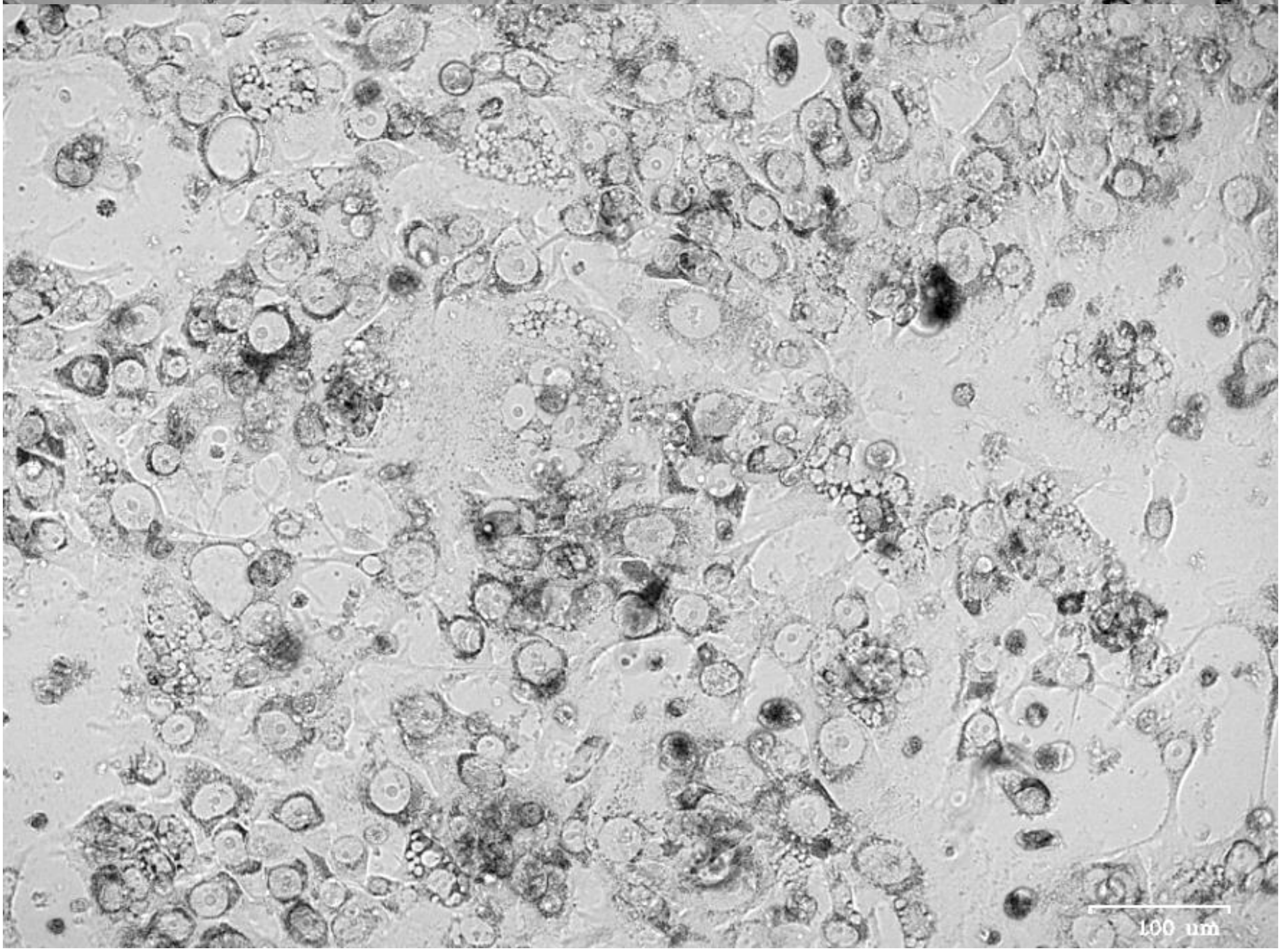
100 um

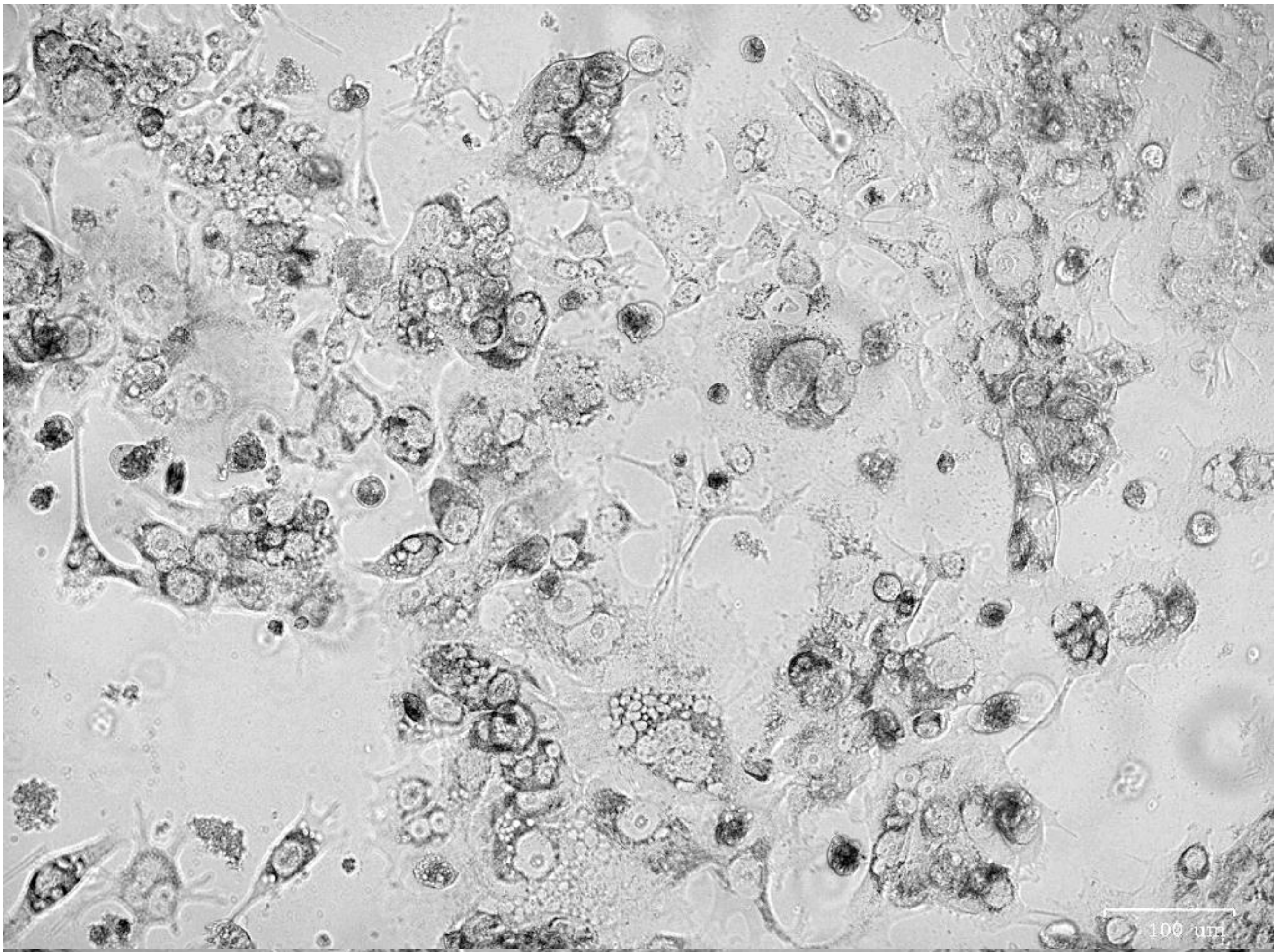
100 um

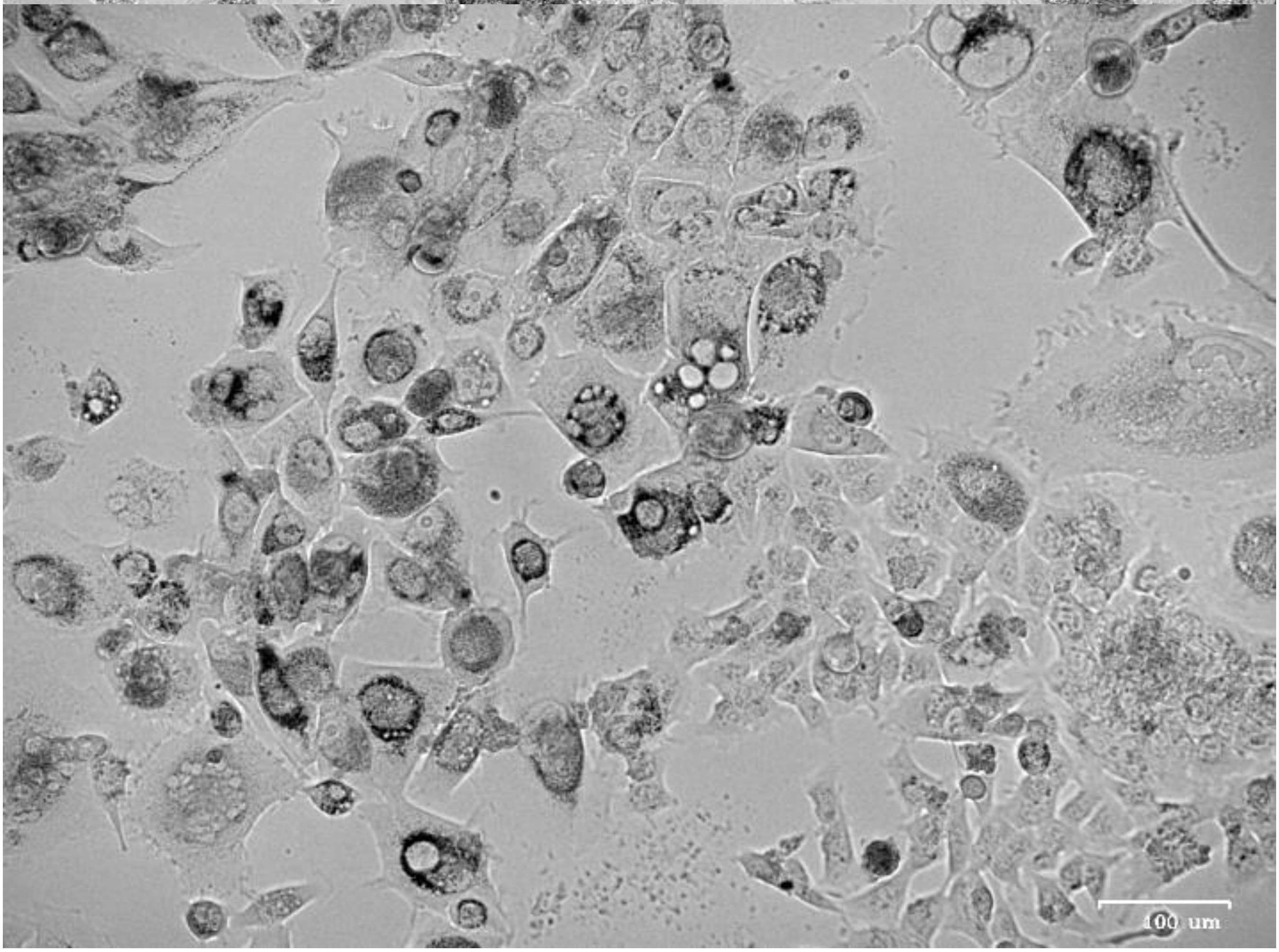
100 um

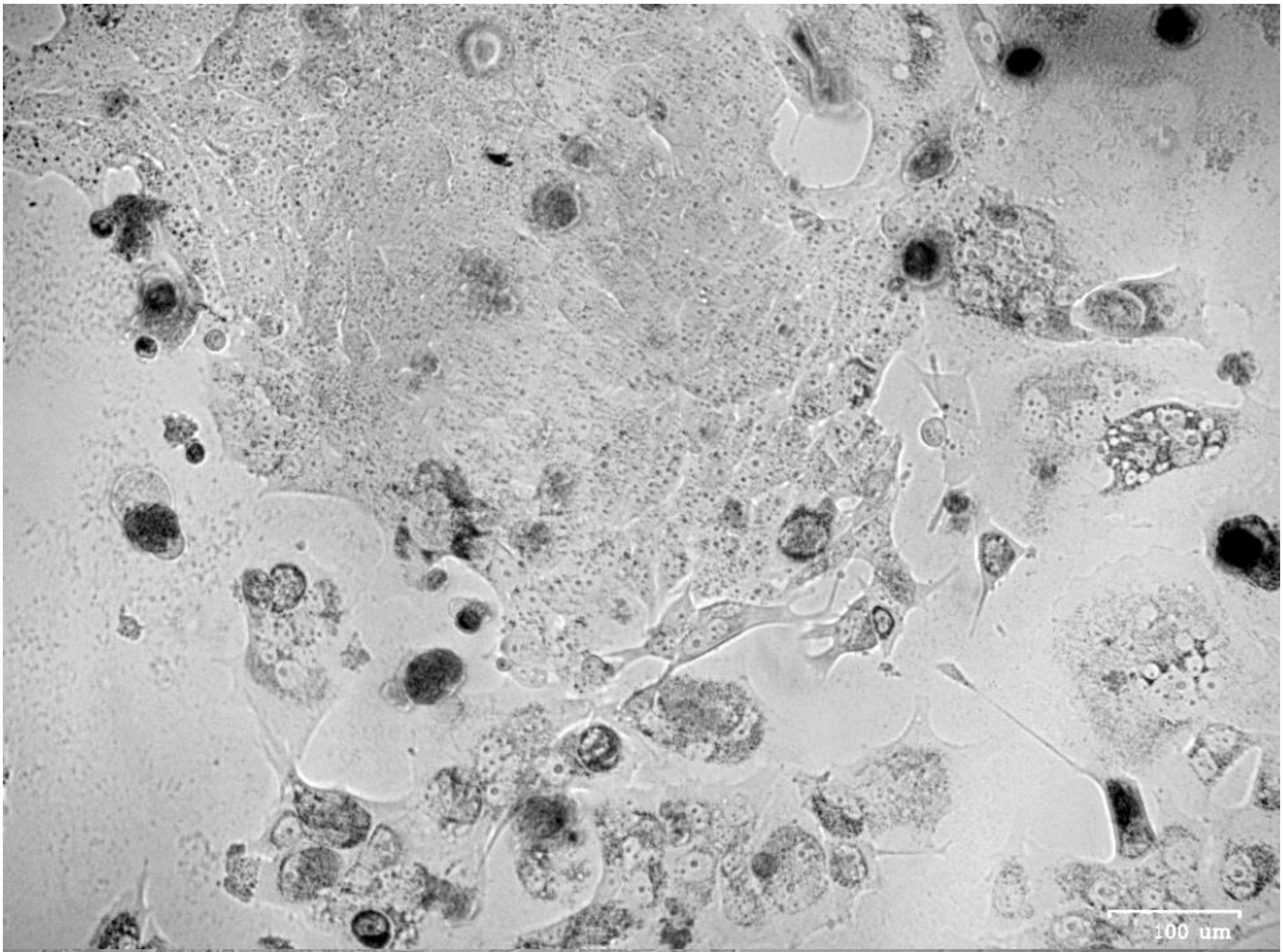
100 um

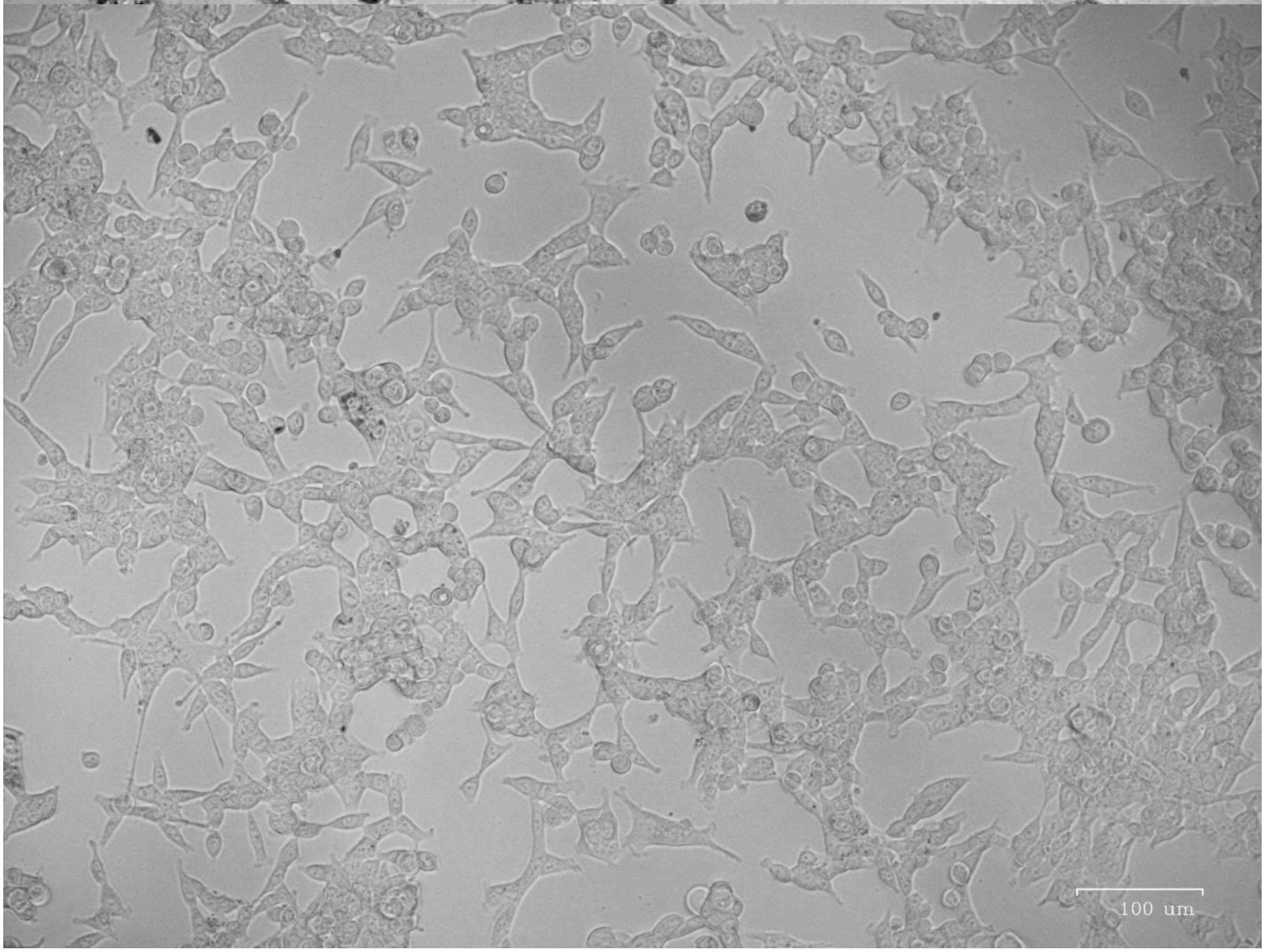
100 um

A

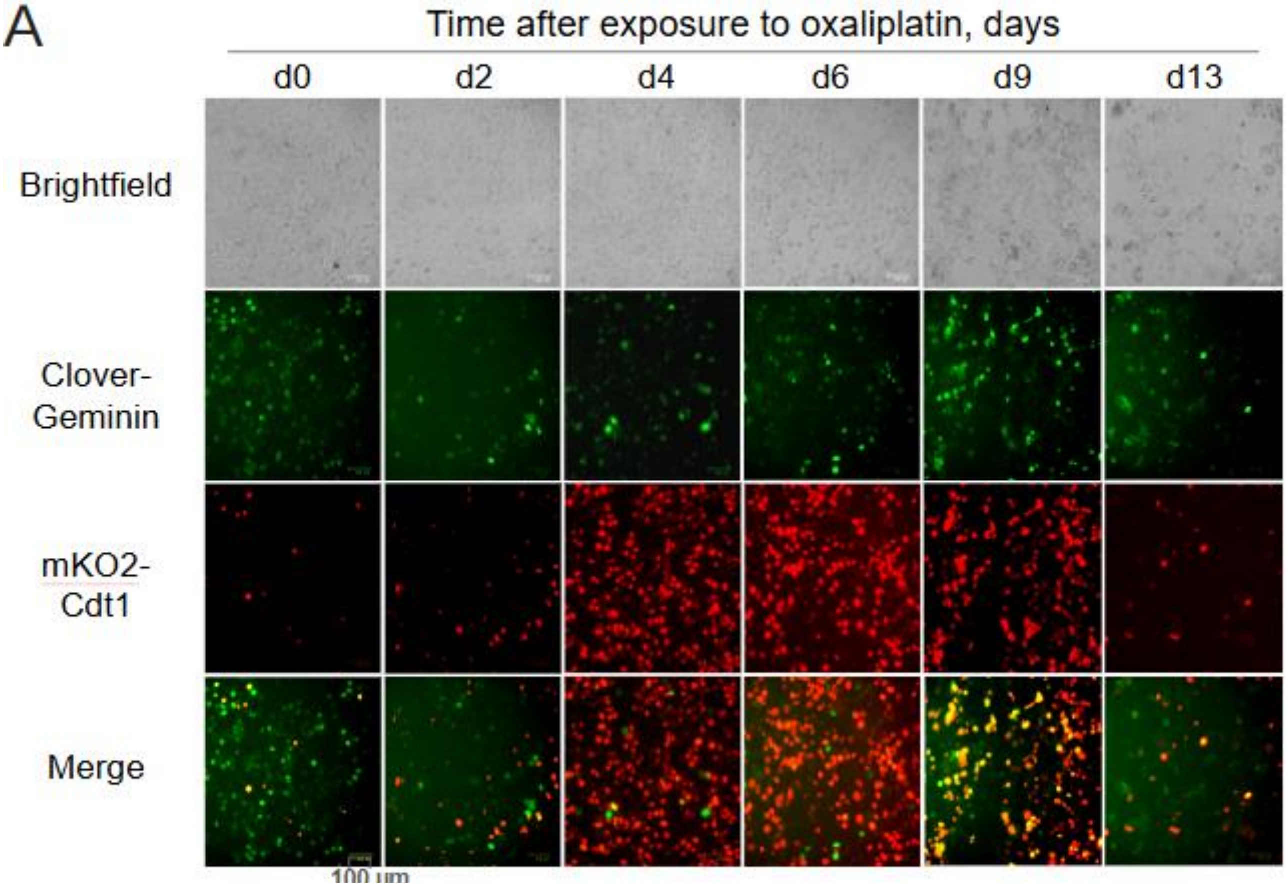
Time after exposure to oxaliplatin, days
d0
d2
d4
d6
d9
d13
Brightfield
Clover-
Geminin
mKO2-
Cdt1
Merge
100 μm

Brightfield images from day 0 to day 13:

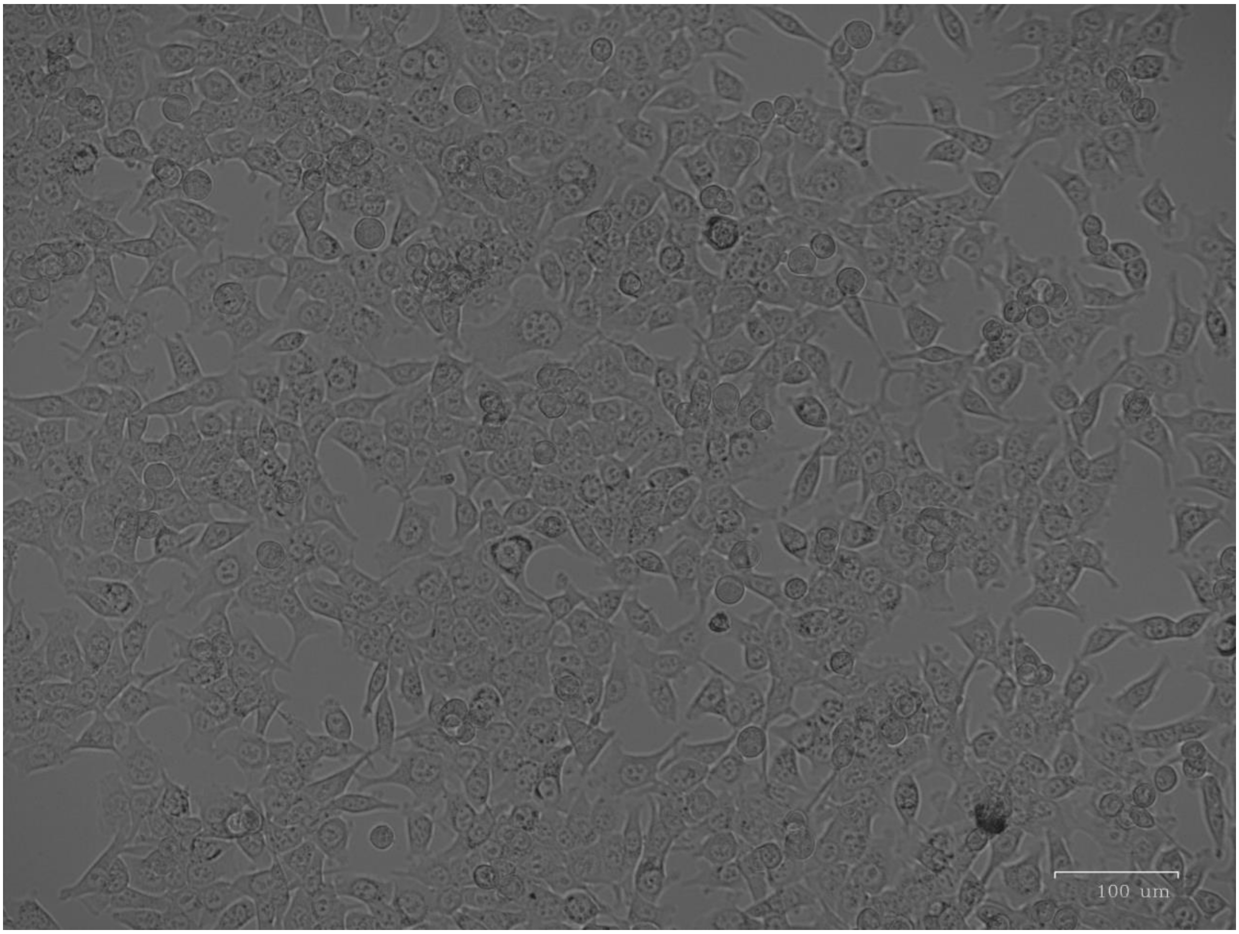

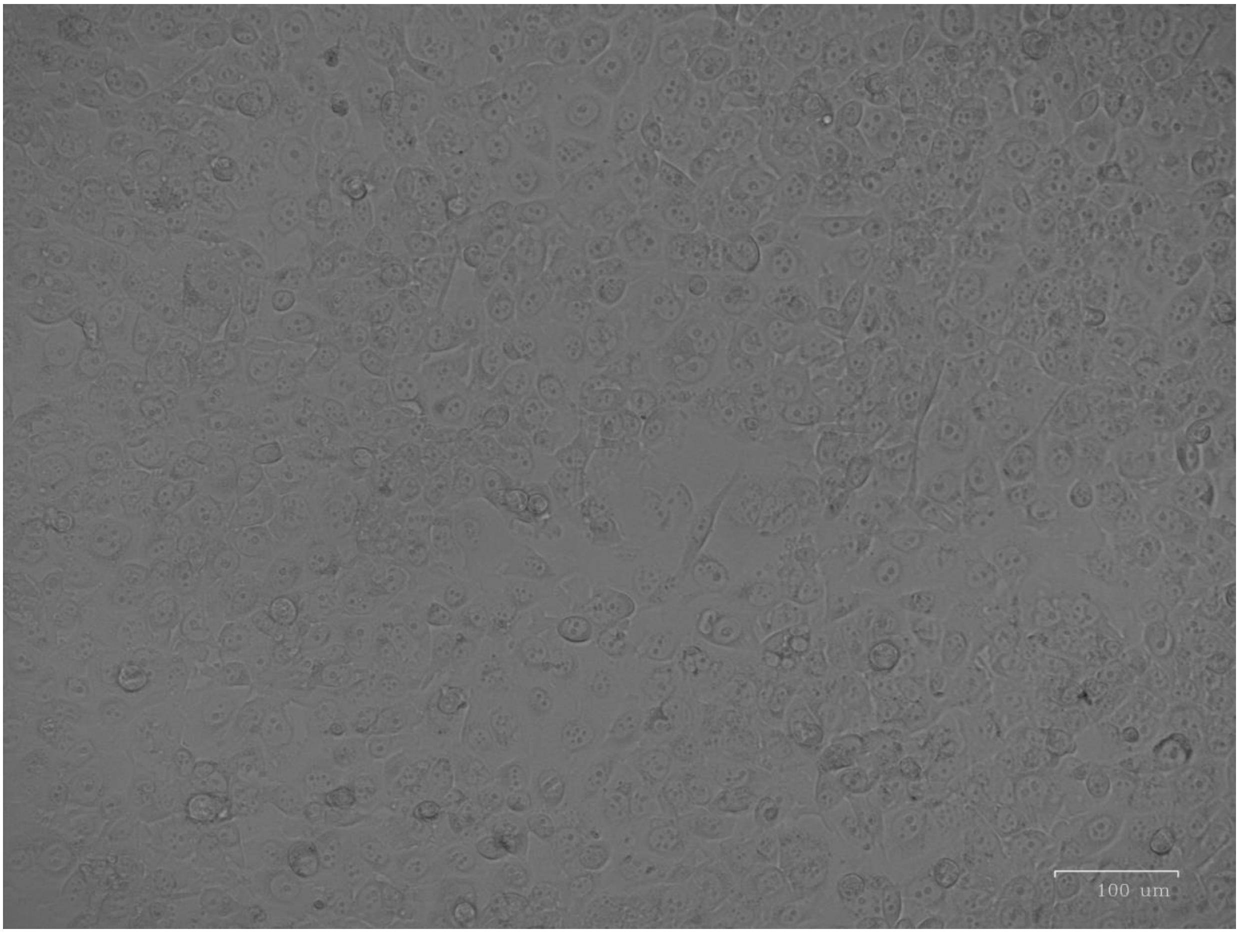

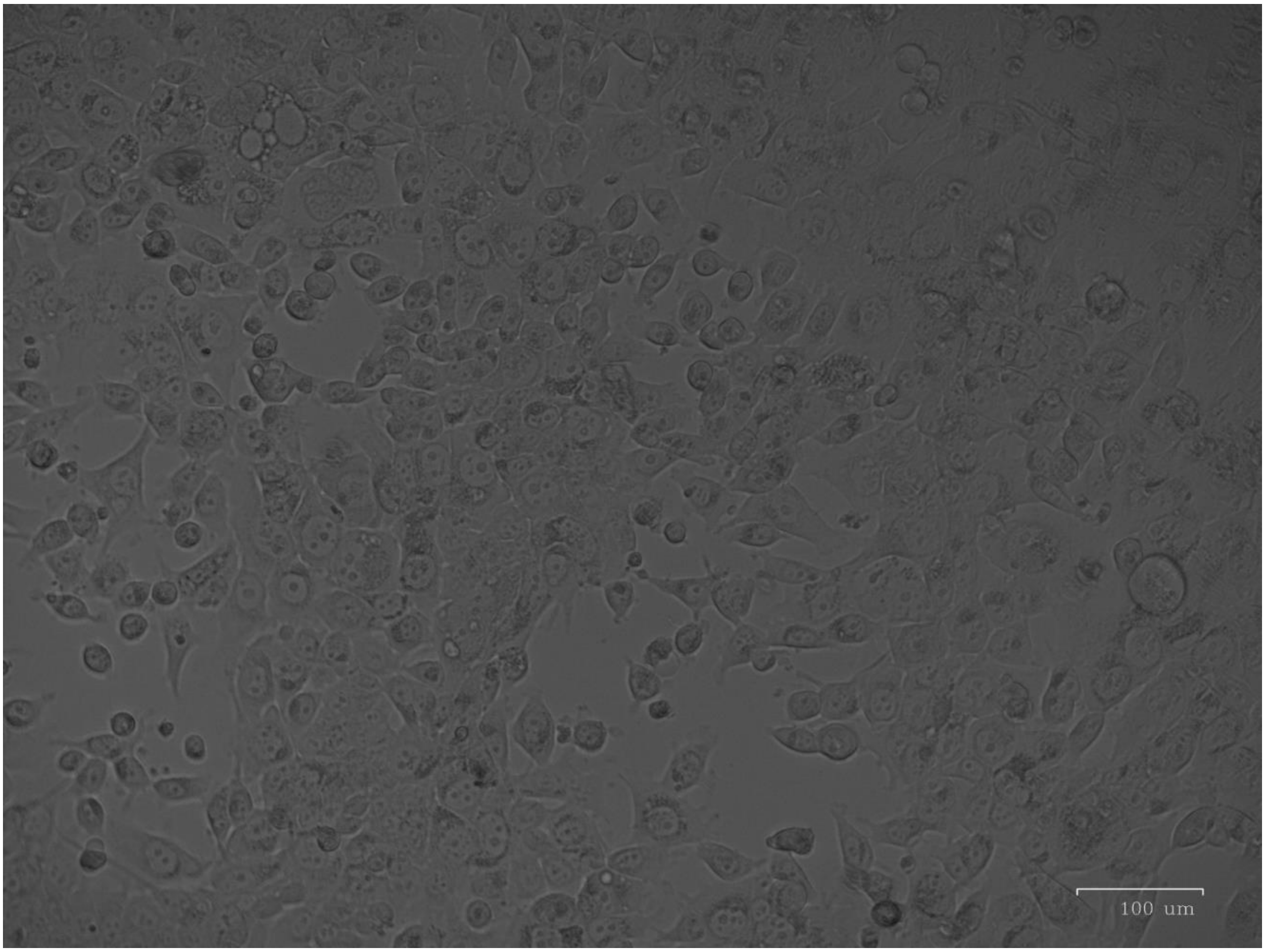
100 um

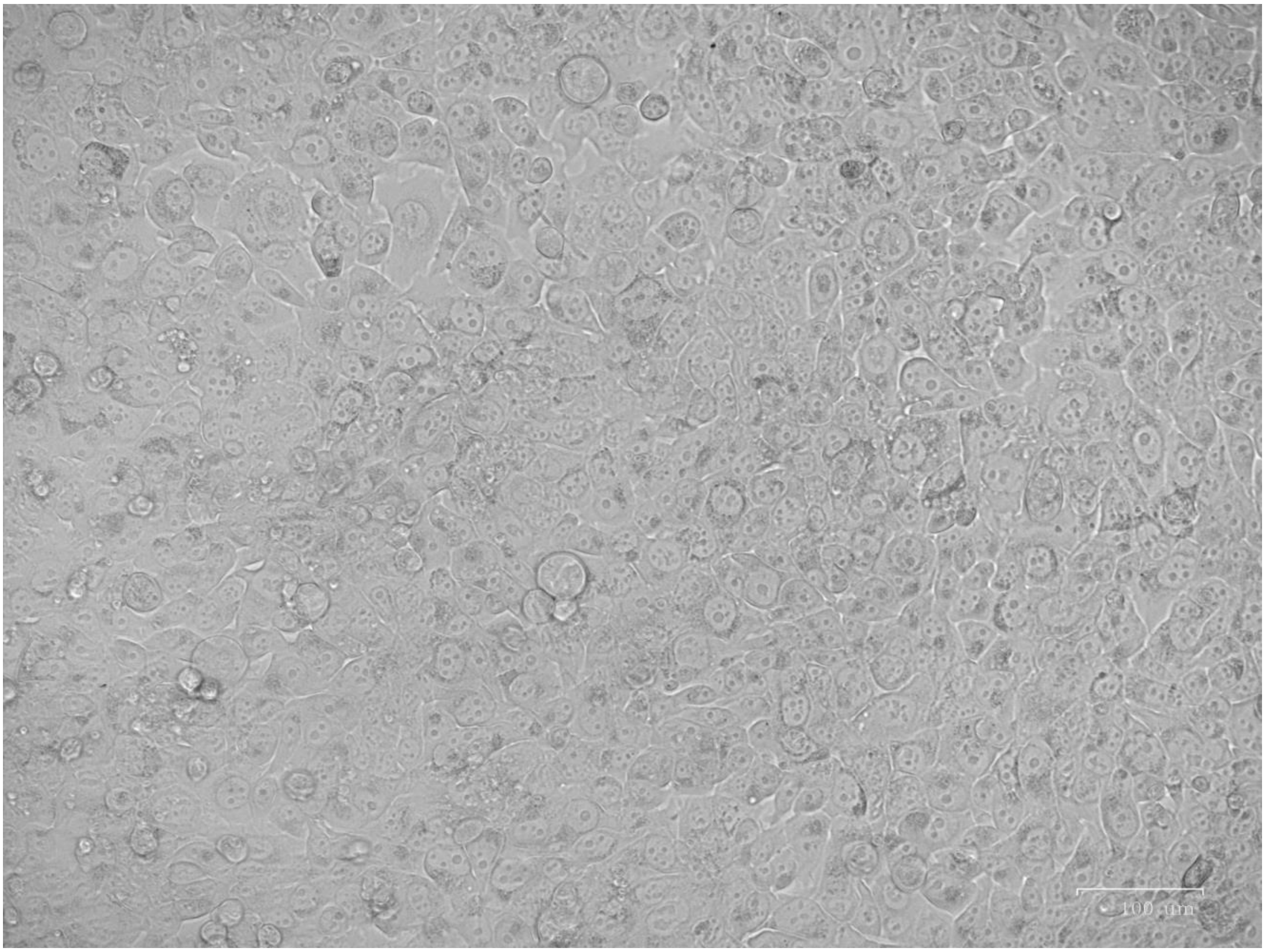
100 um

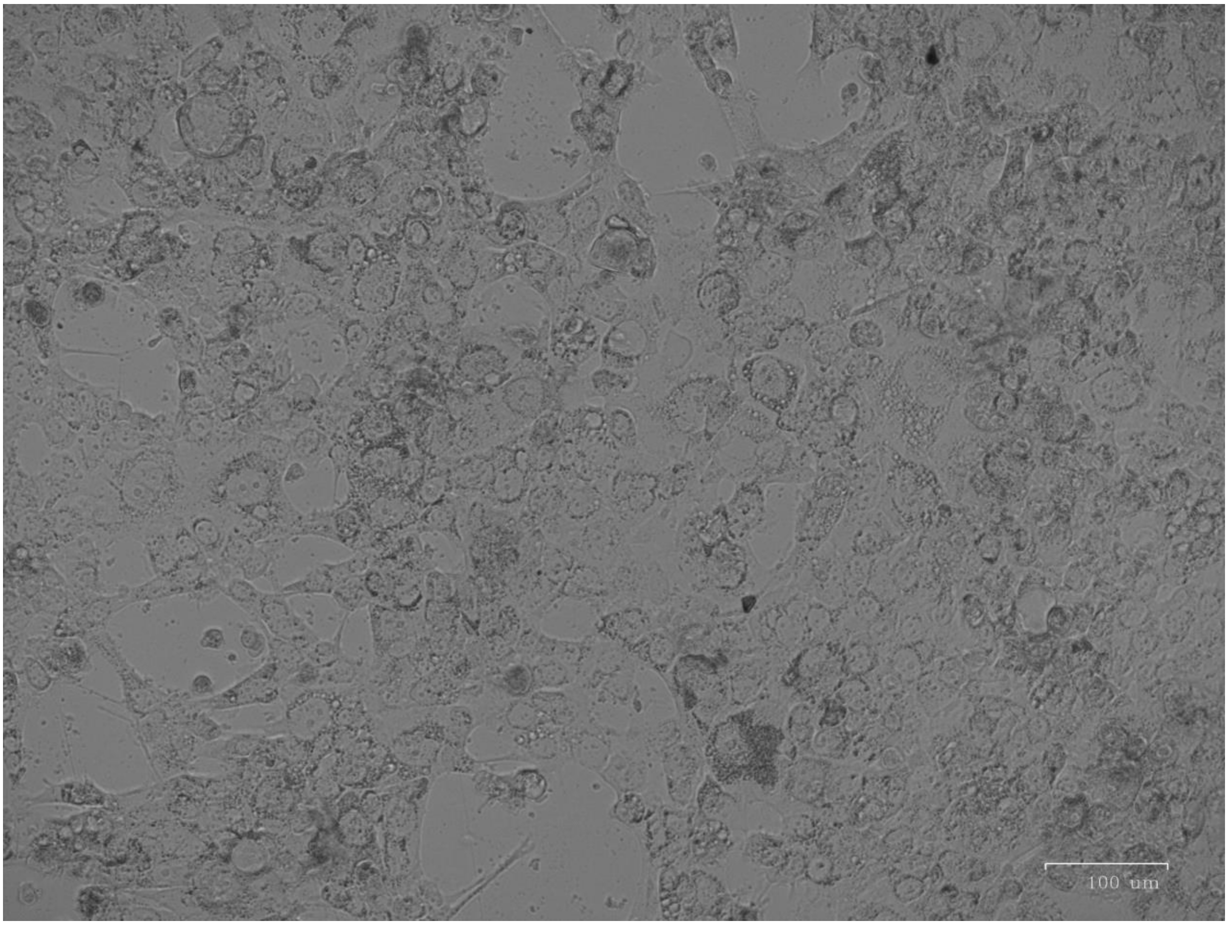
100 um

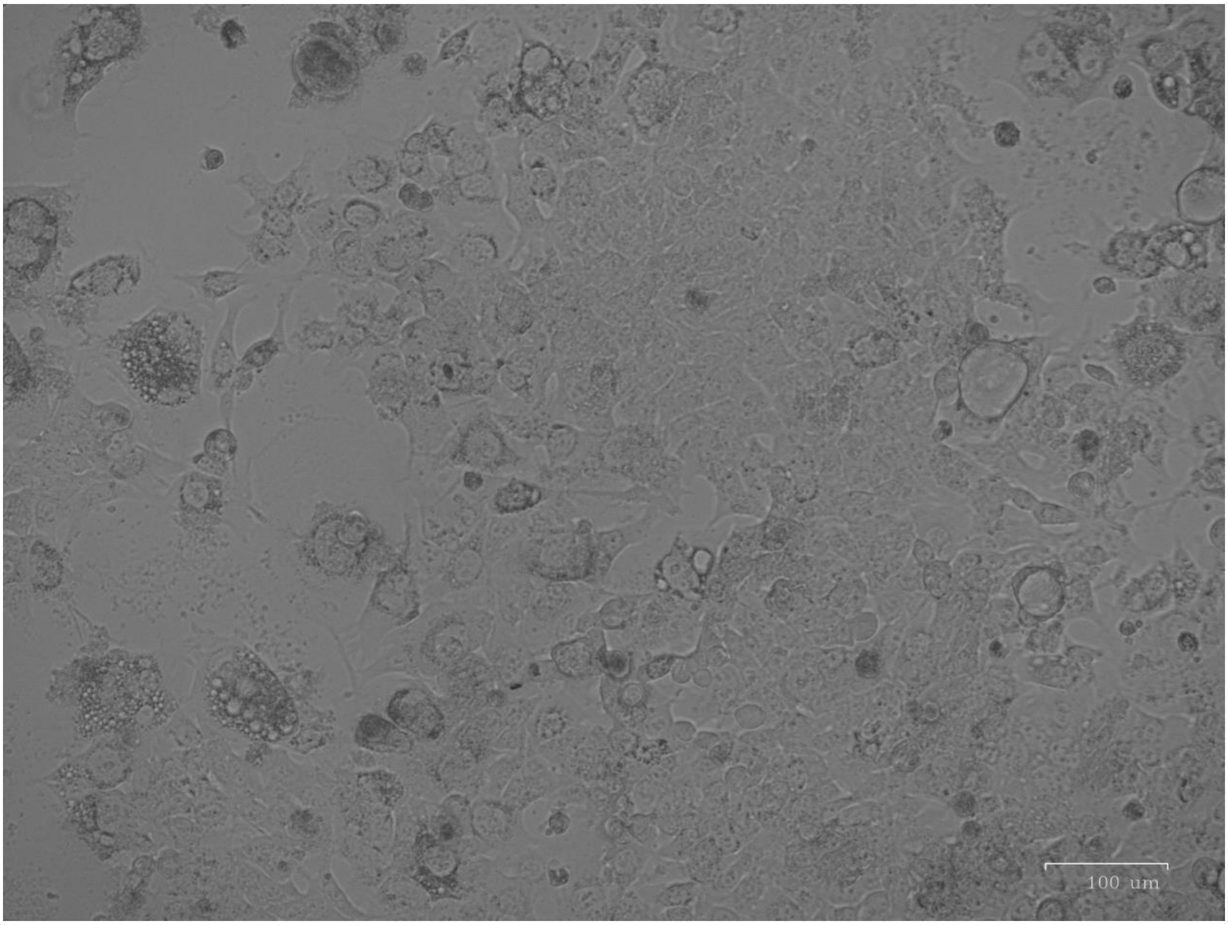
100 um

Red images from day 0 to day 13:

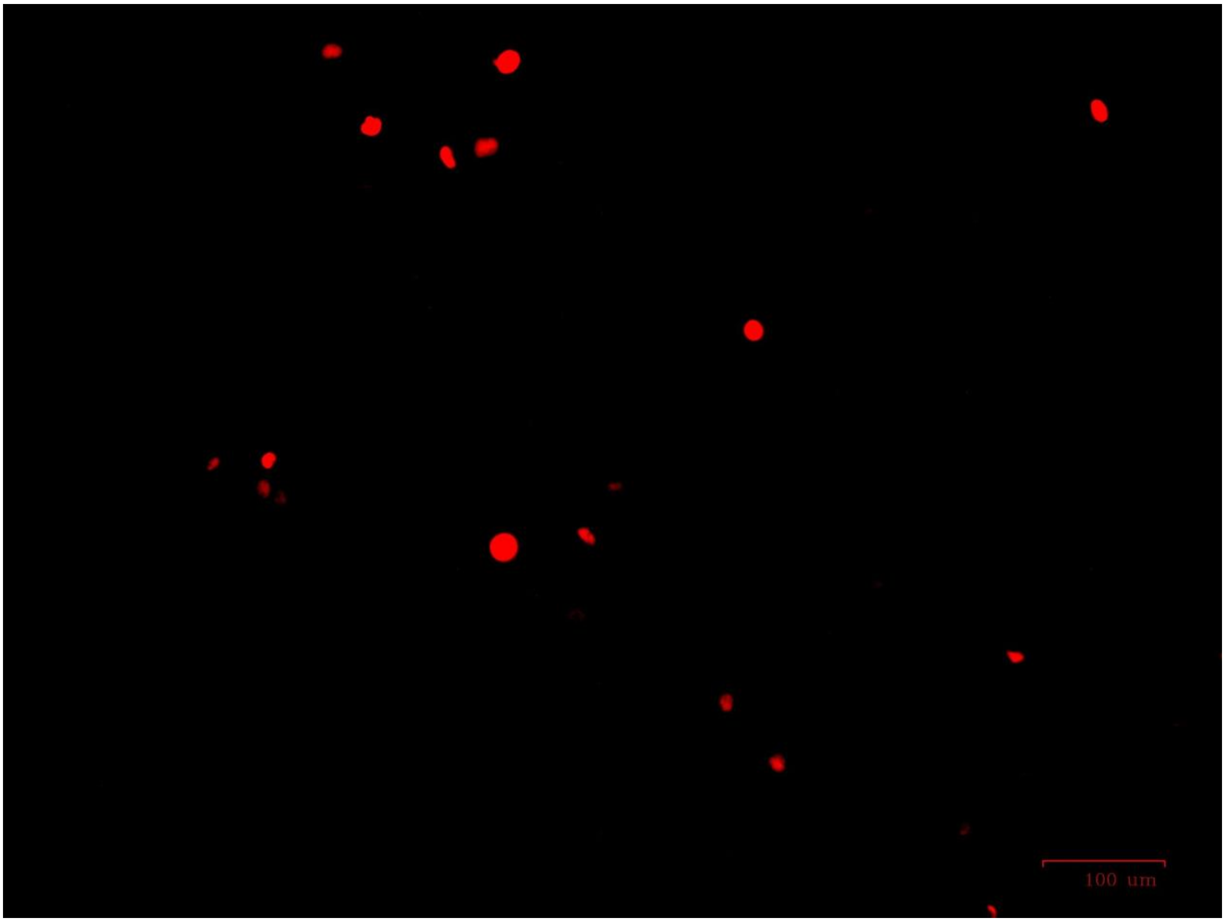

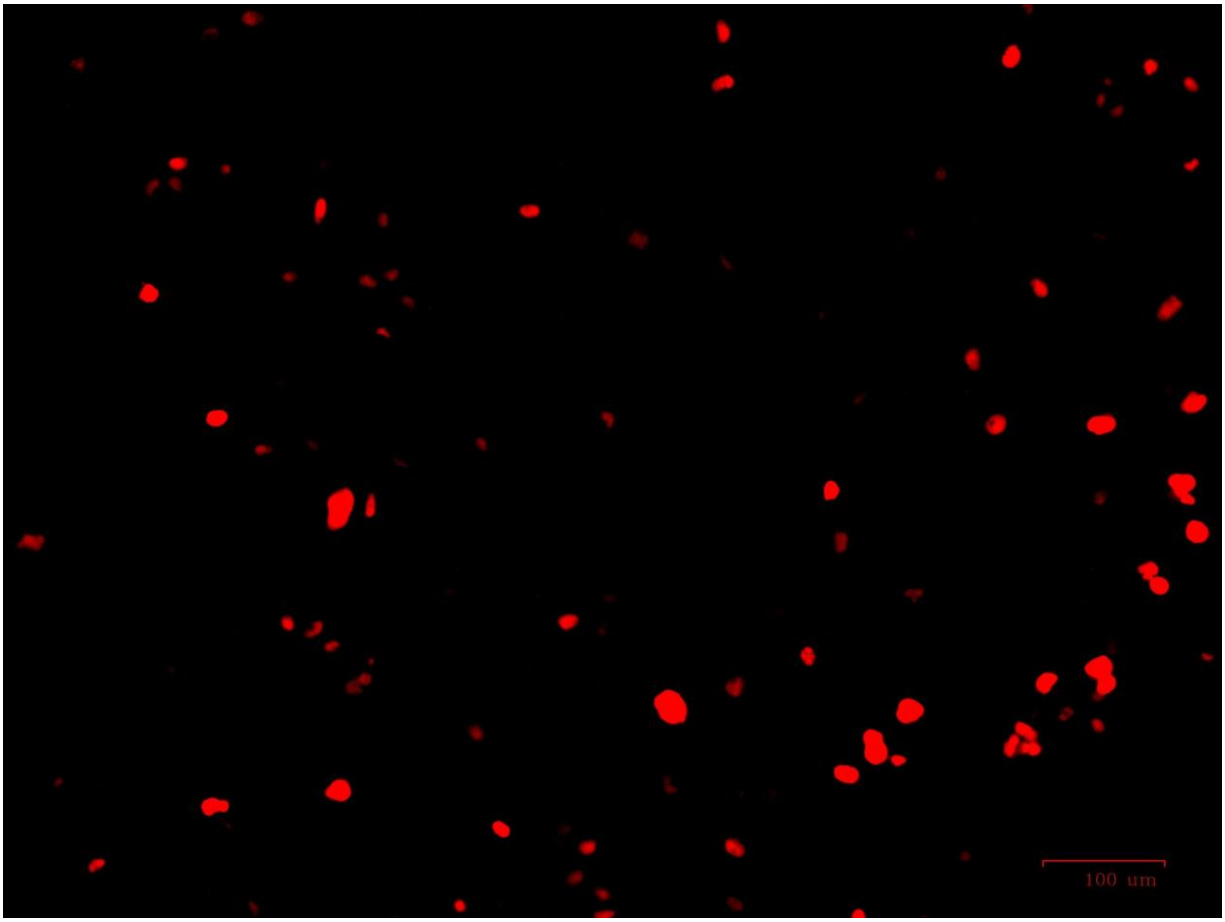

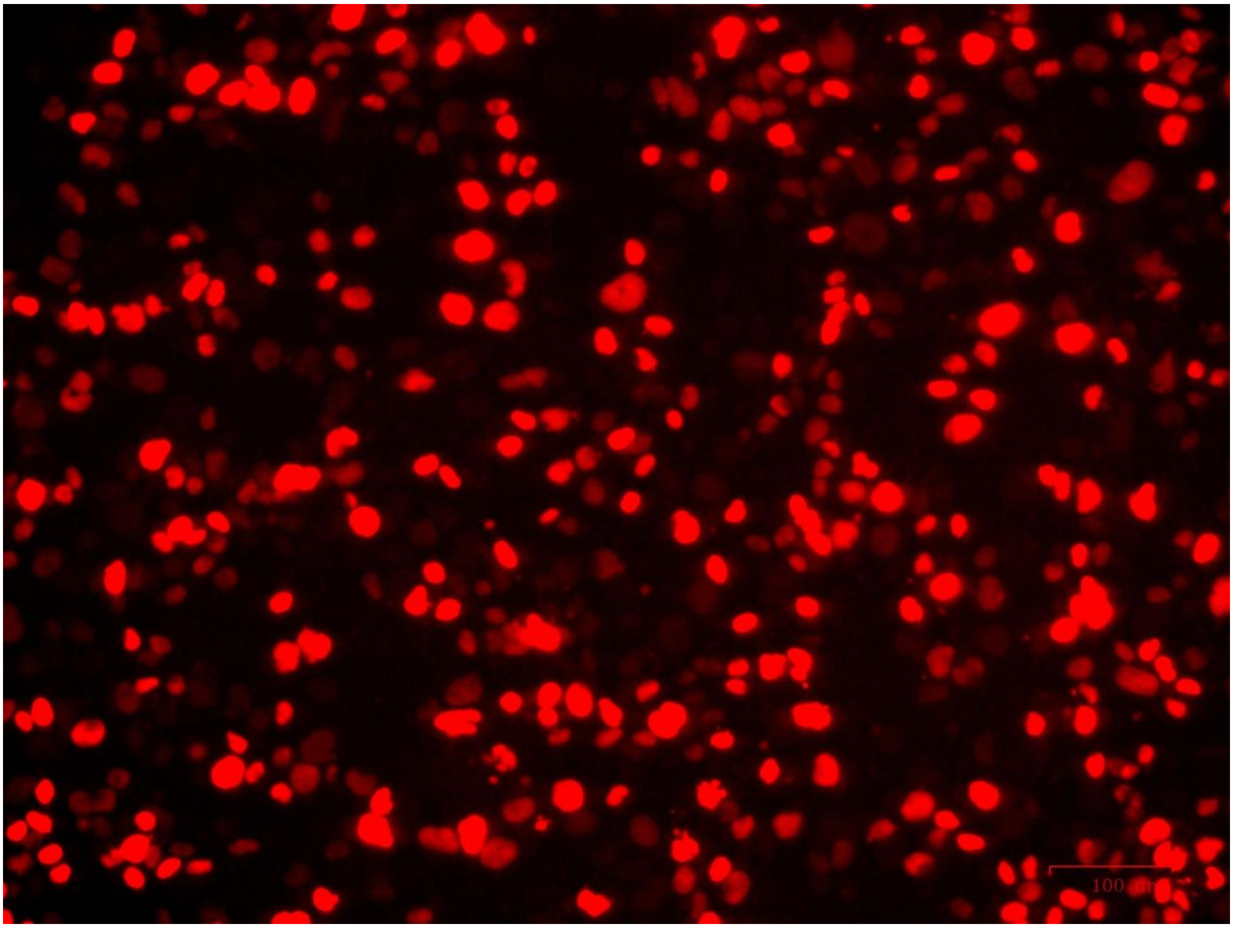
100

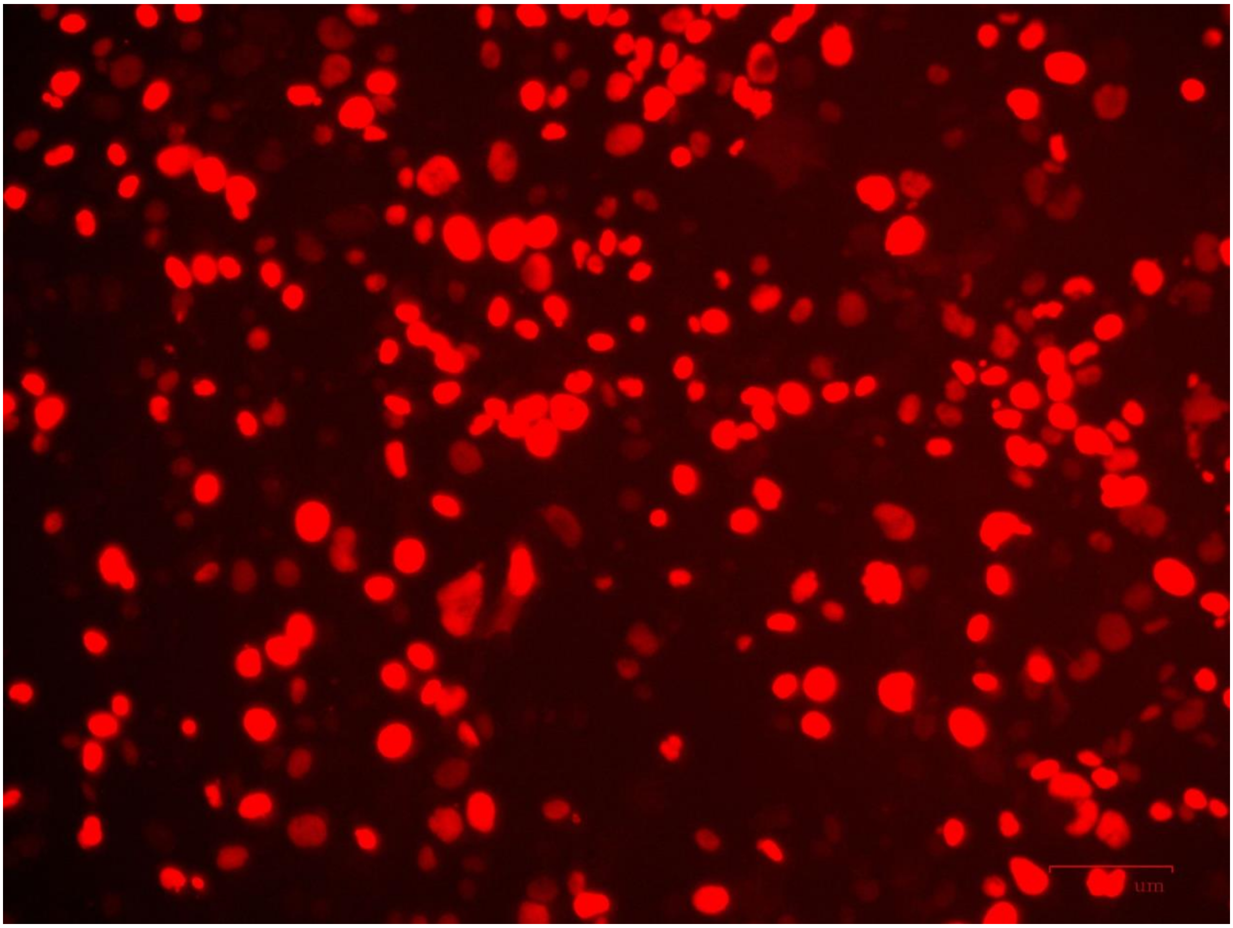
um

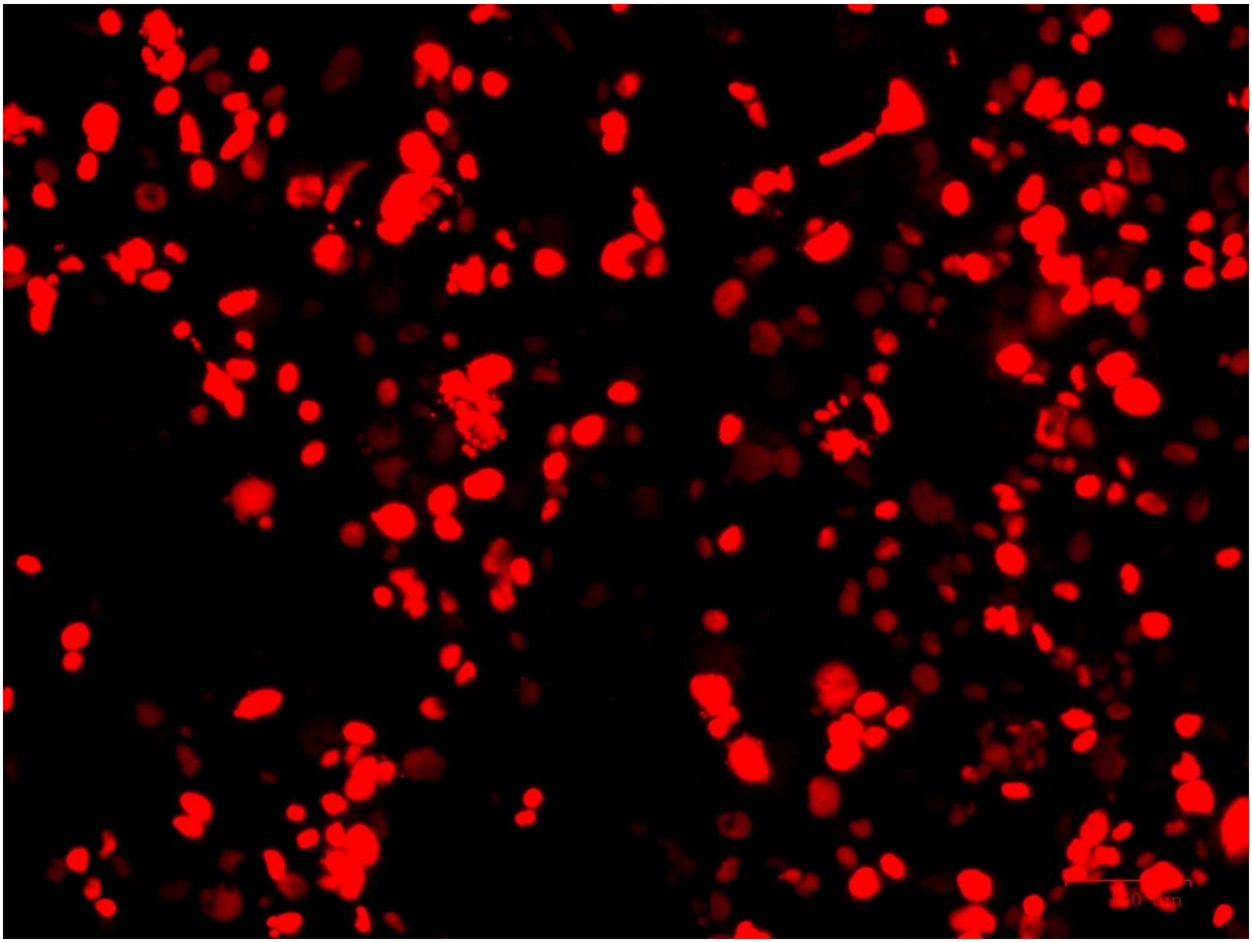

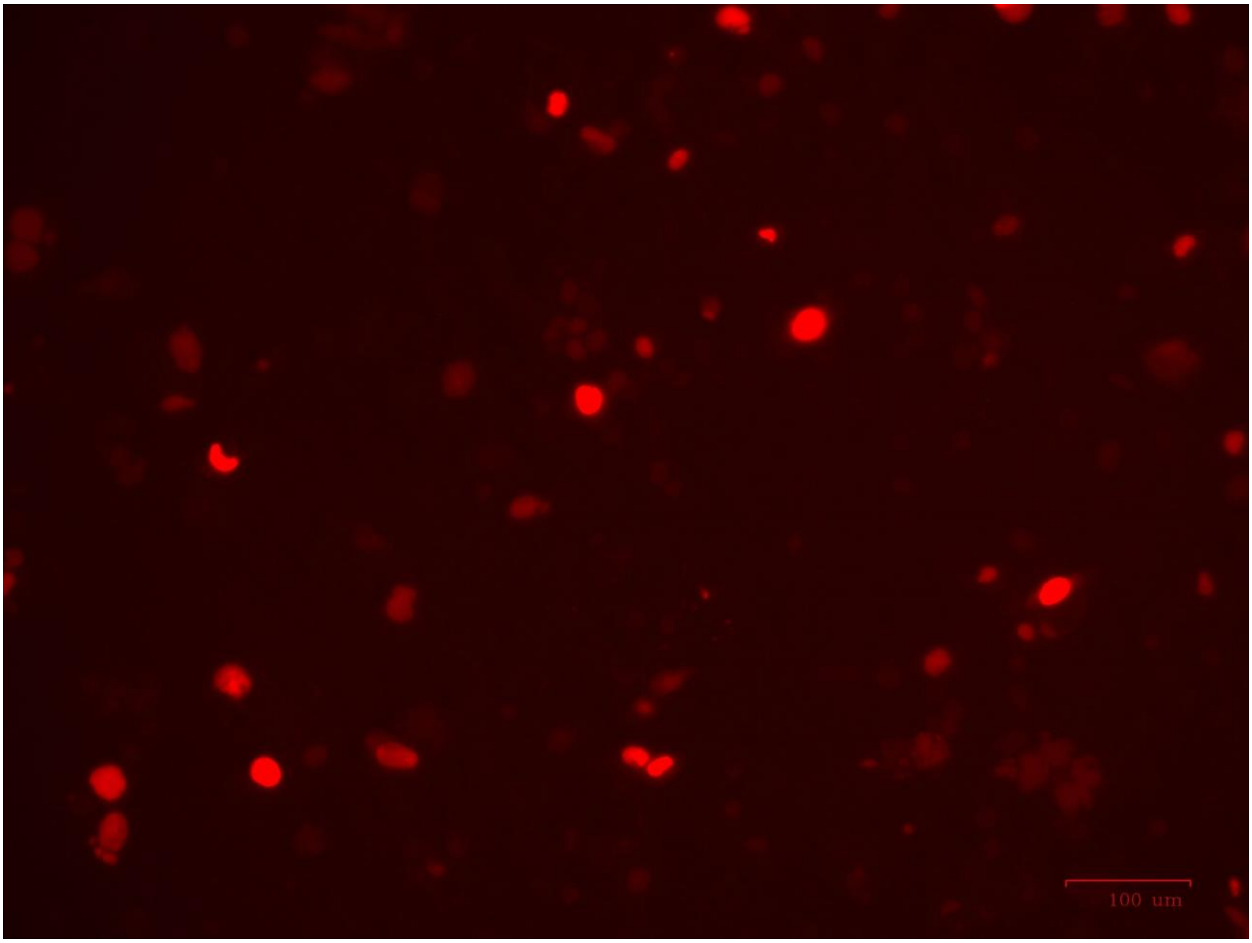
100 um

Green images from day 0 to day 13:

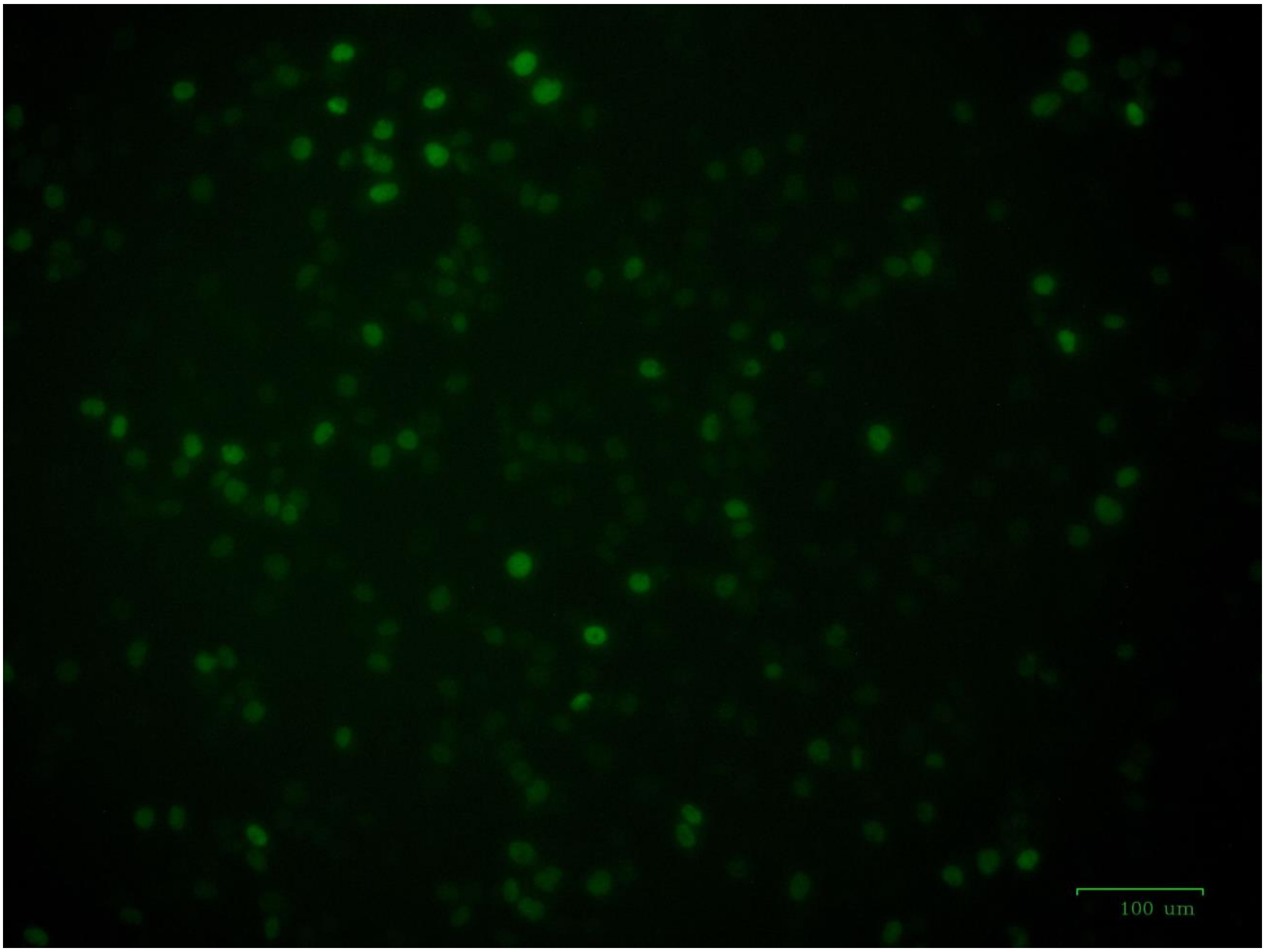

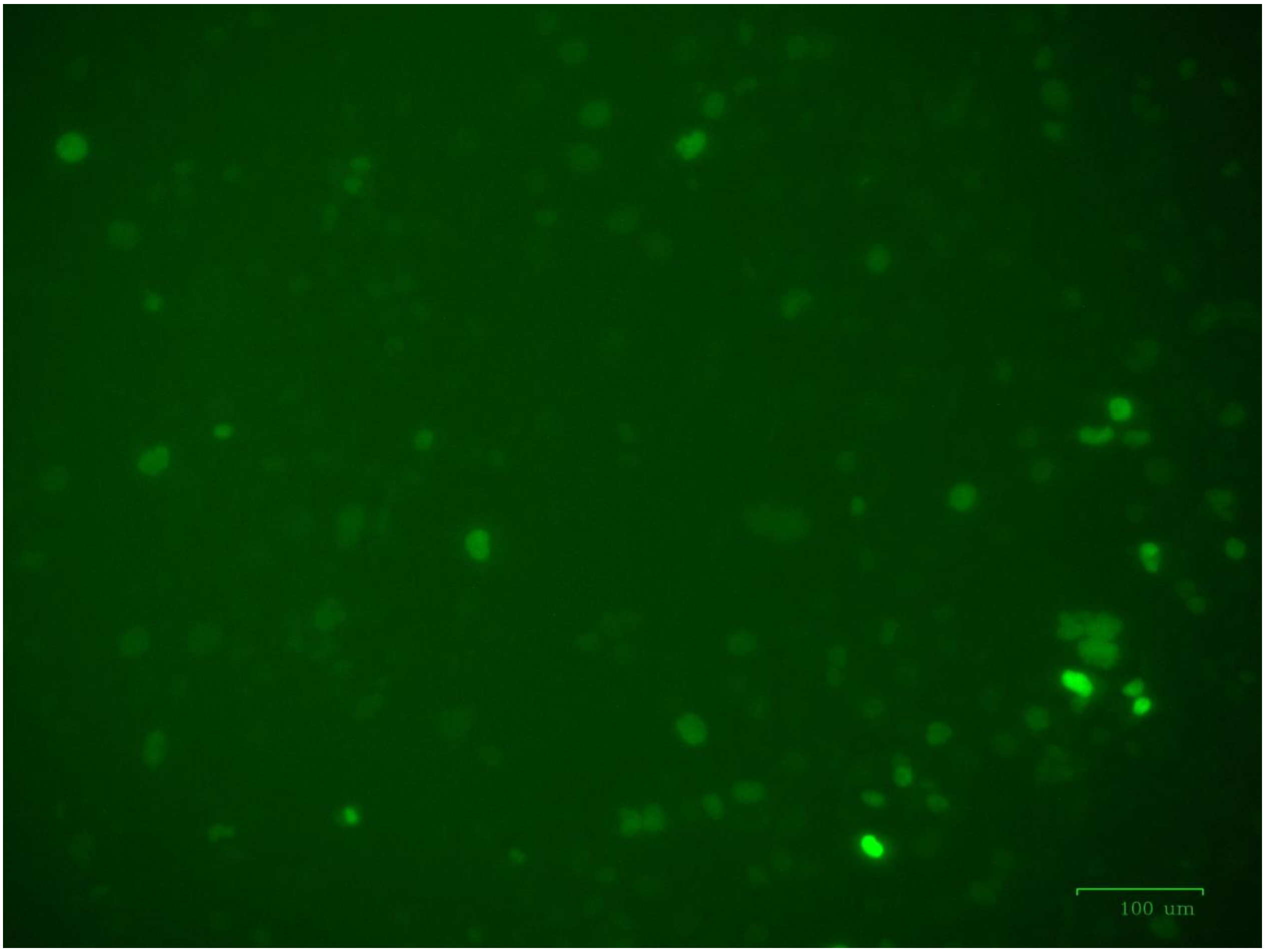

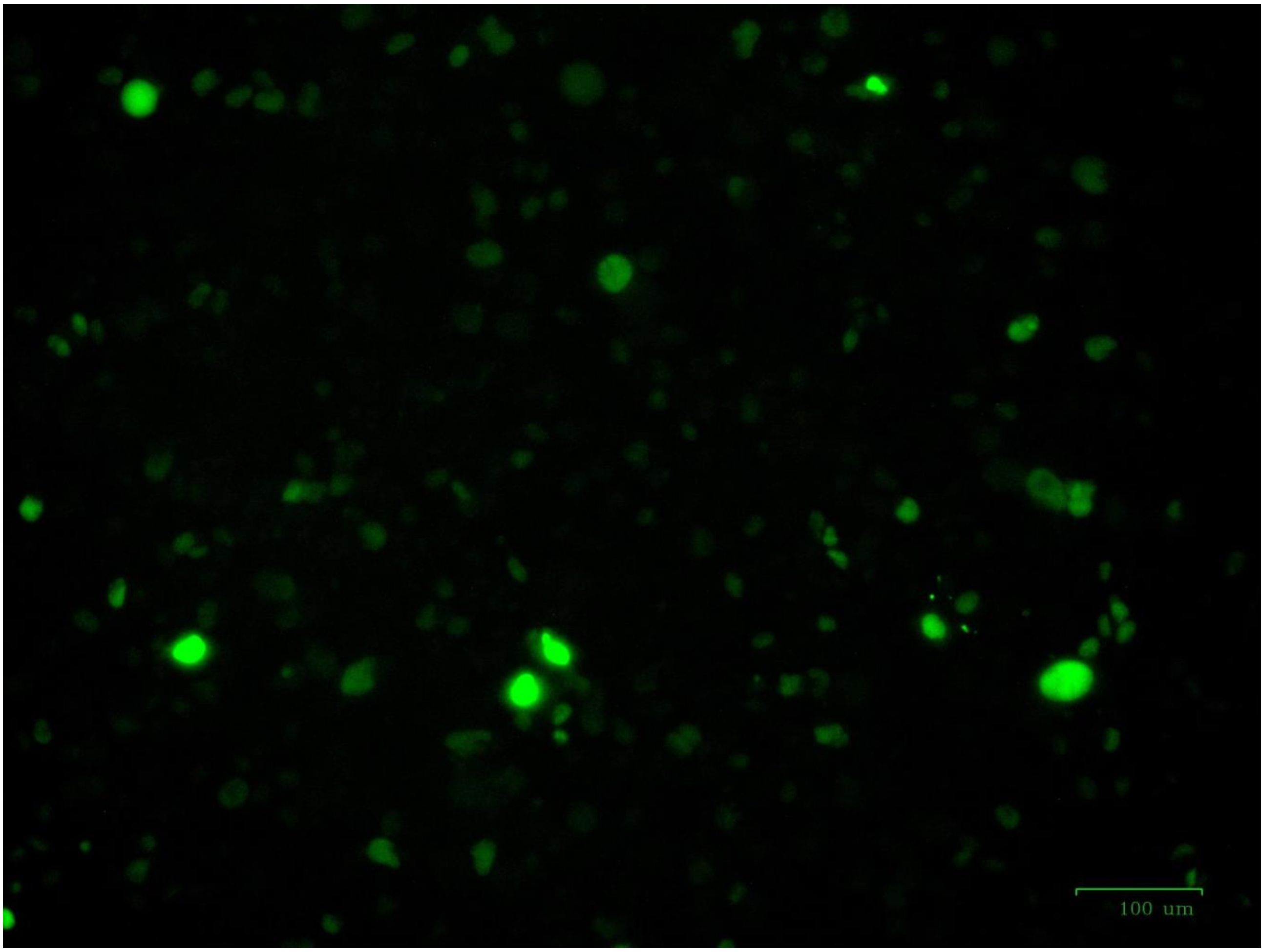
100 um

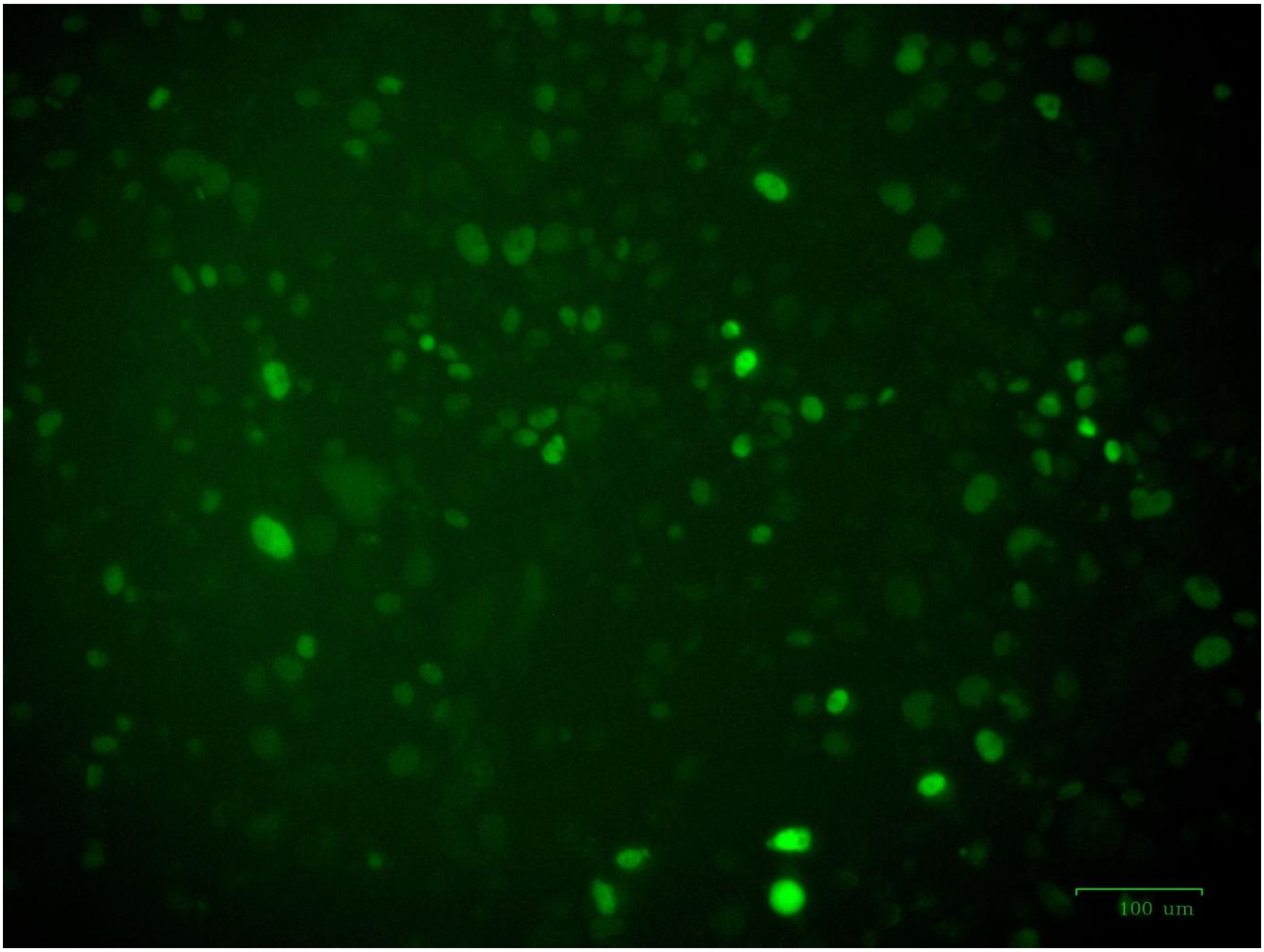
100 um

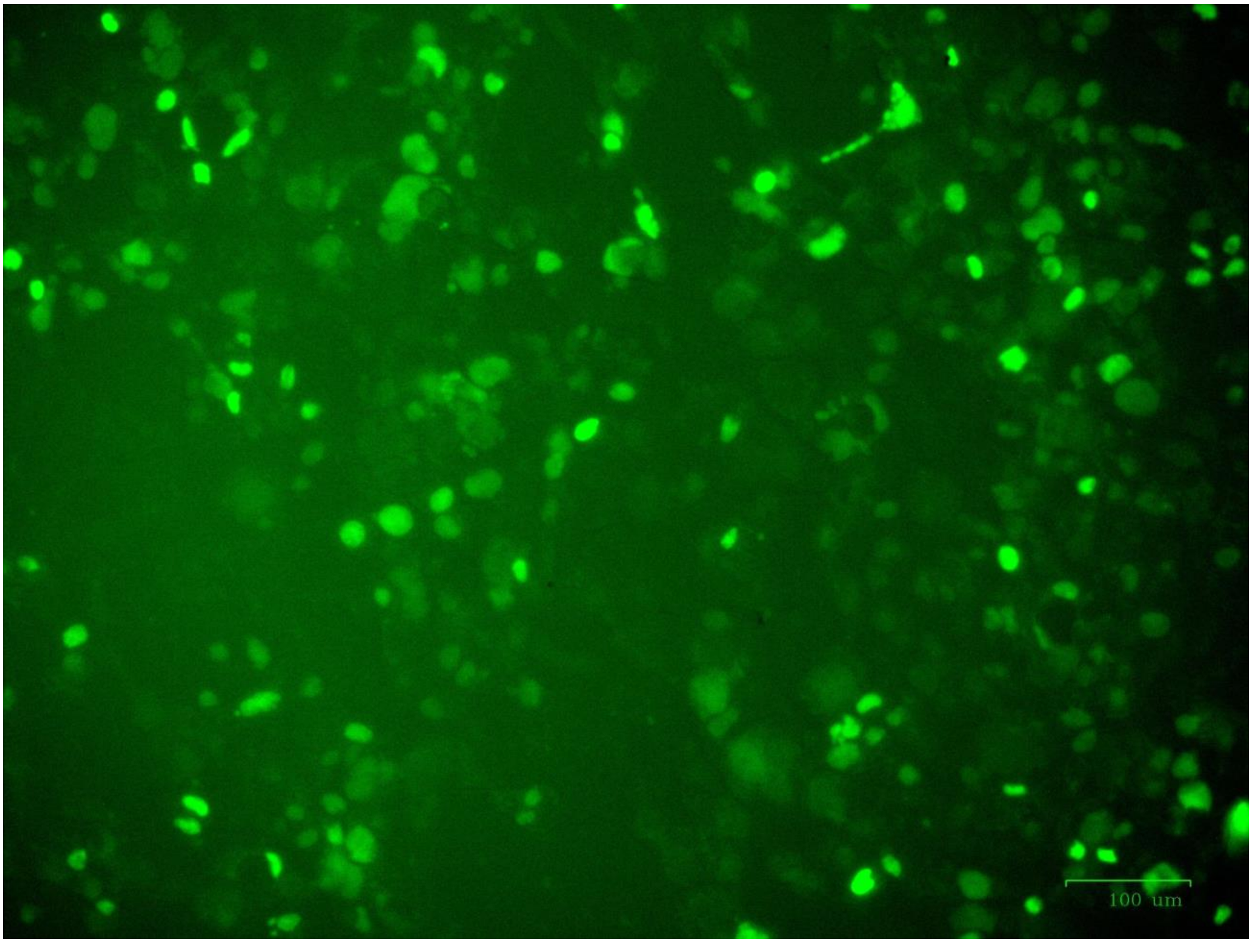
100 um

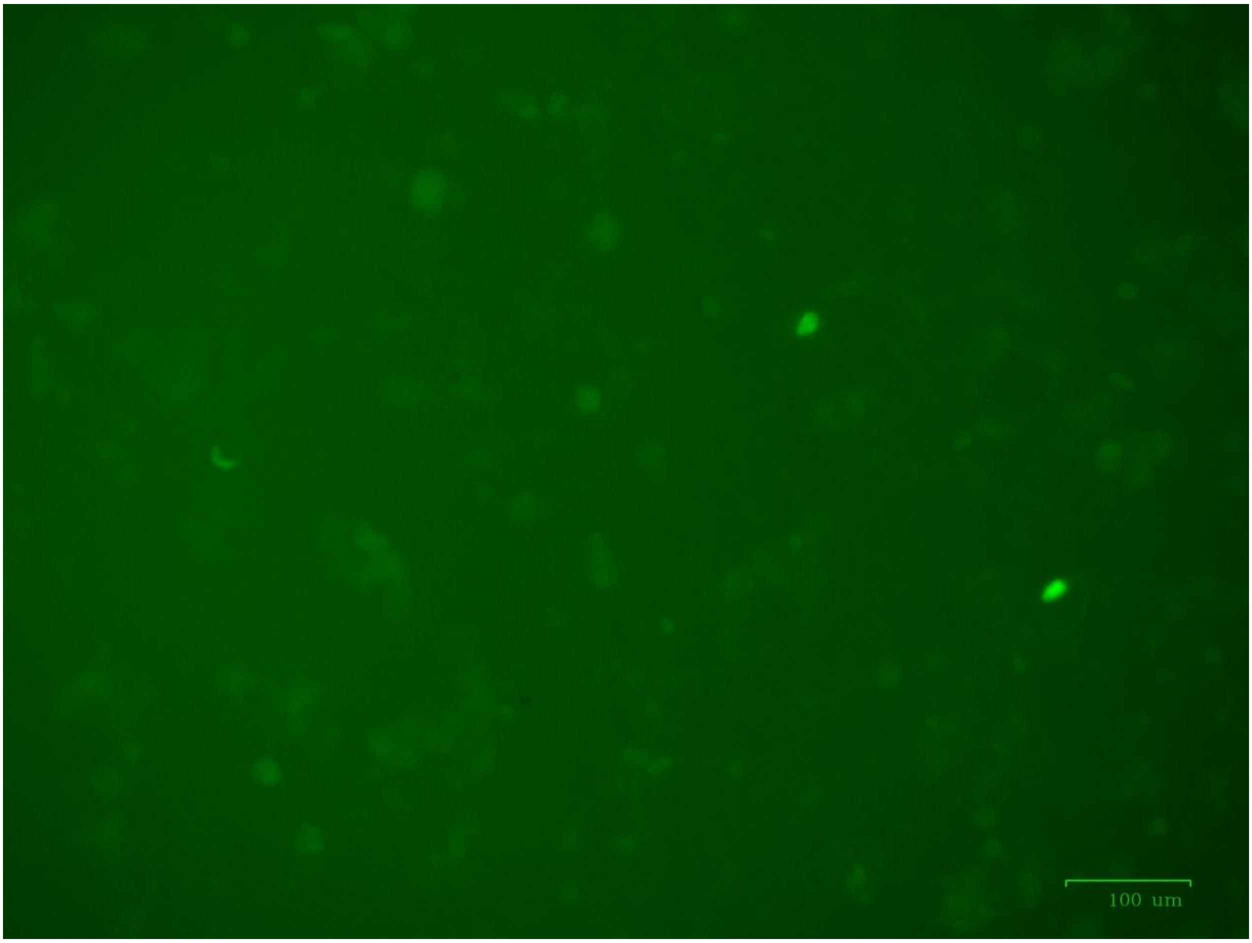
100 um

Merged images from day 0 to day 13:

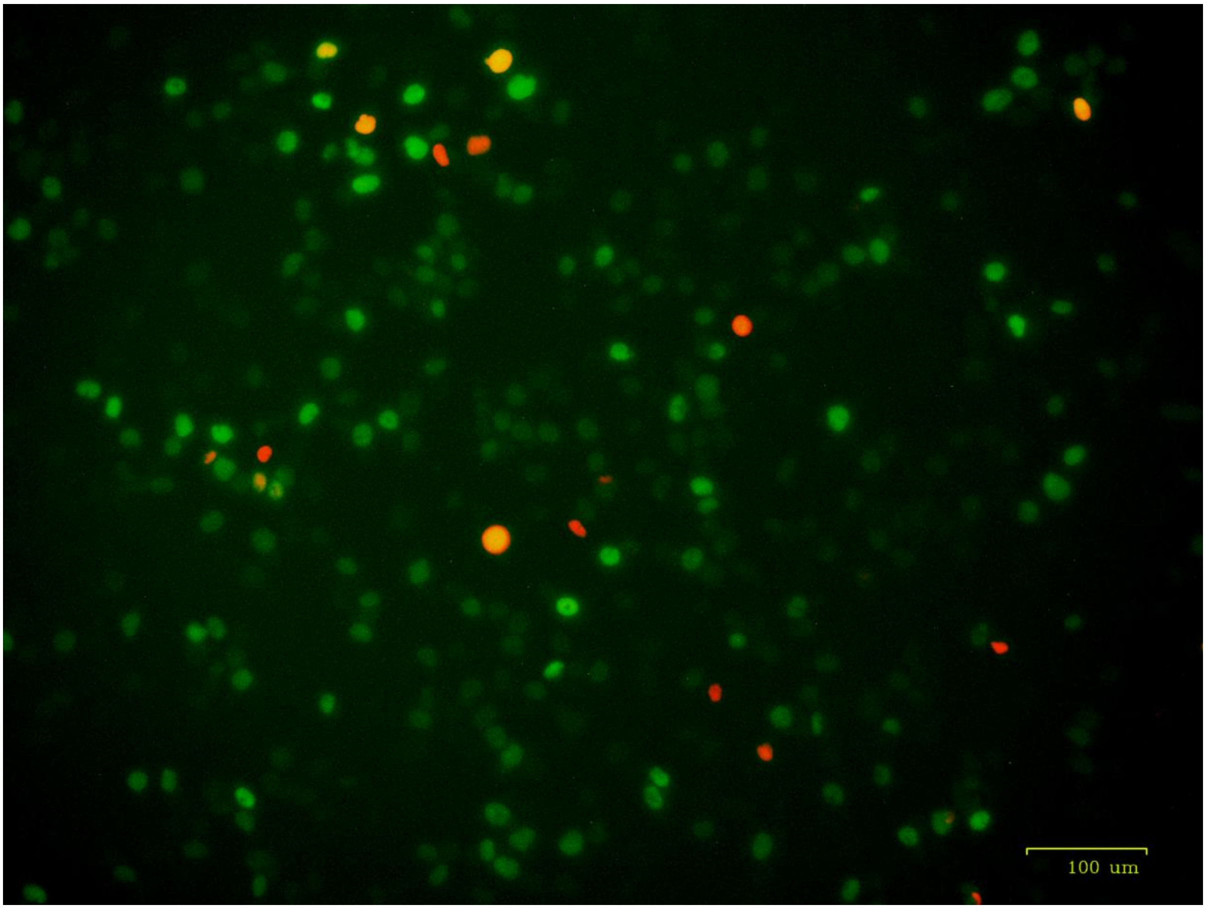

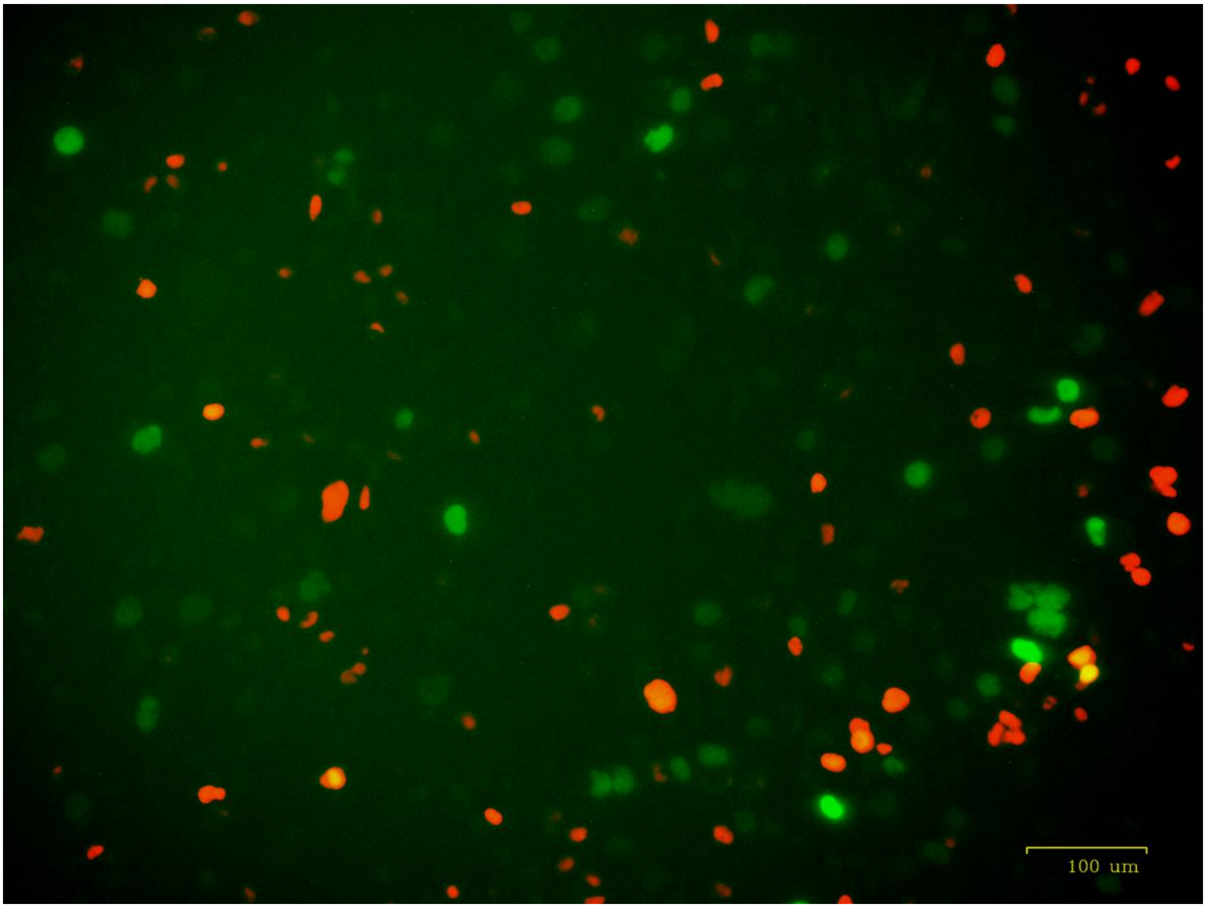

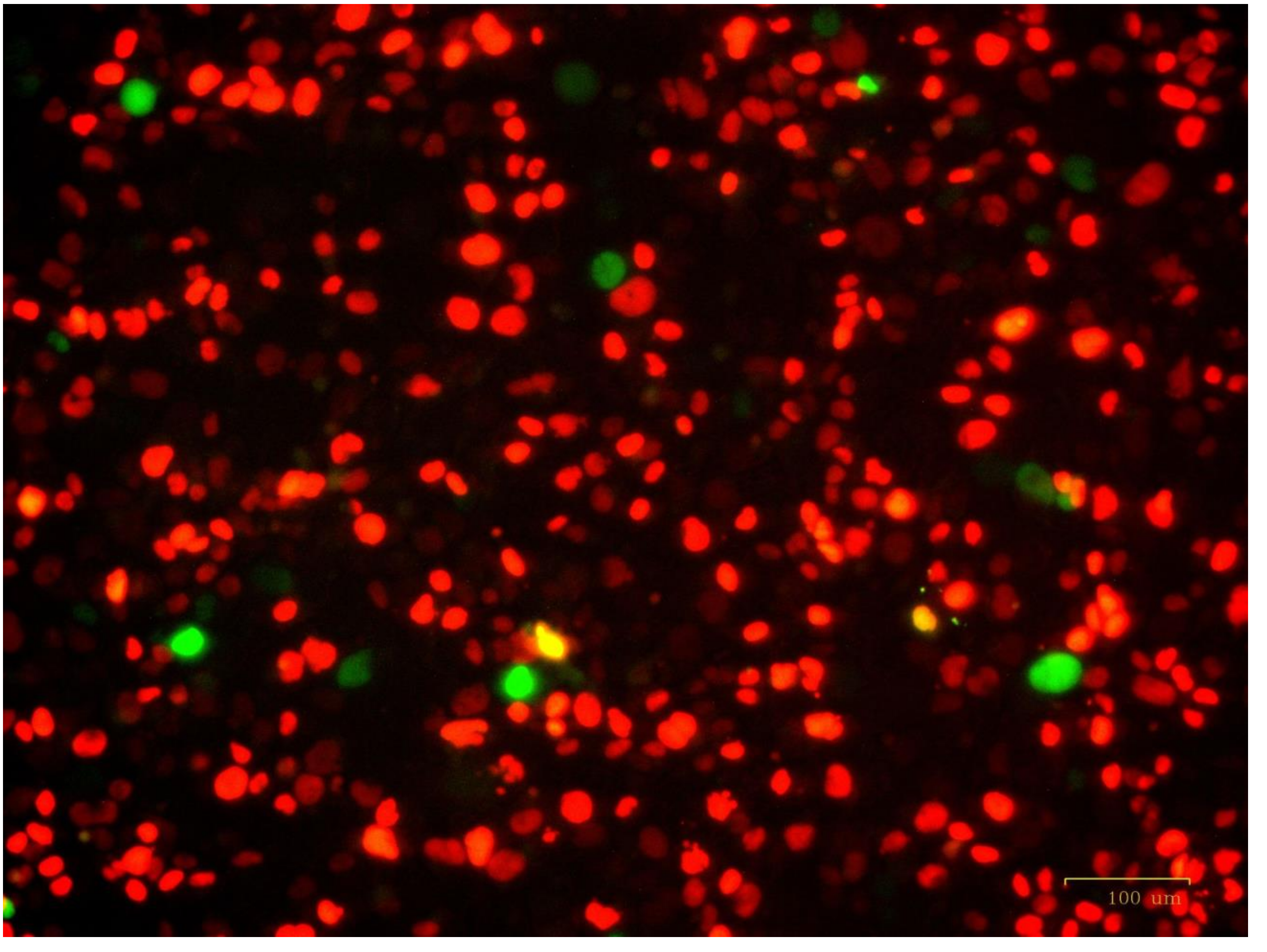
100 um

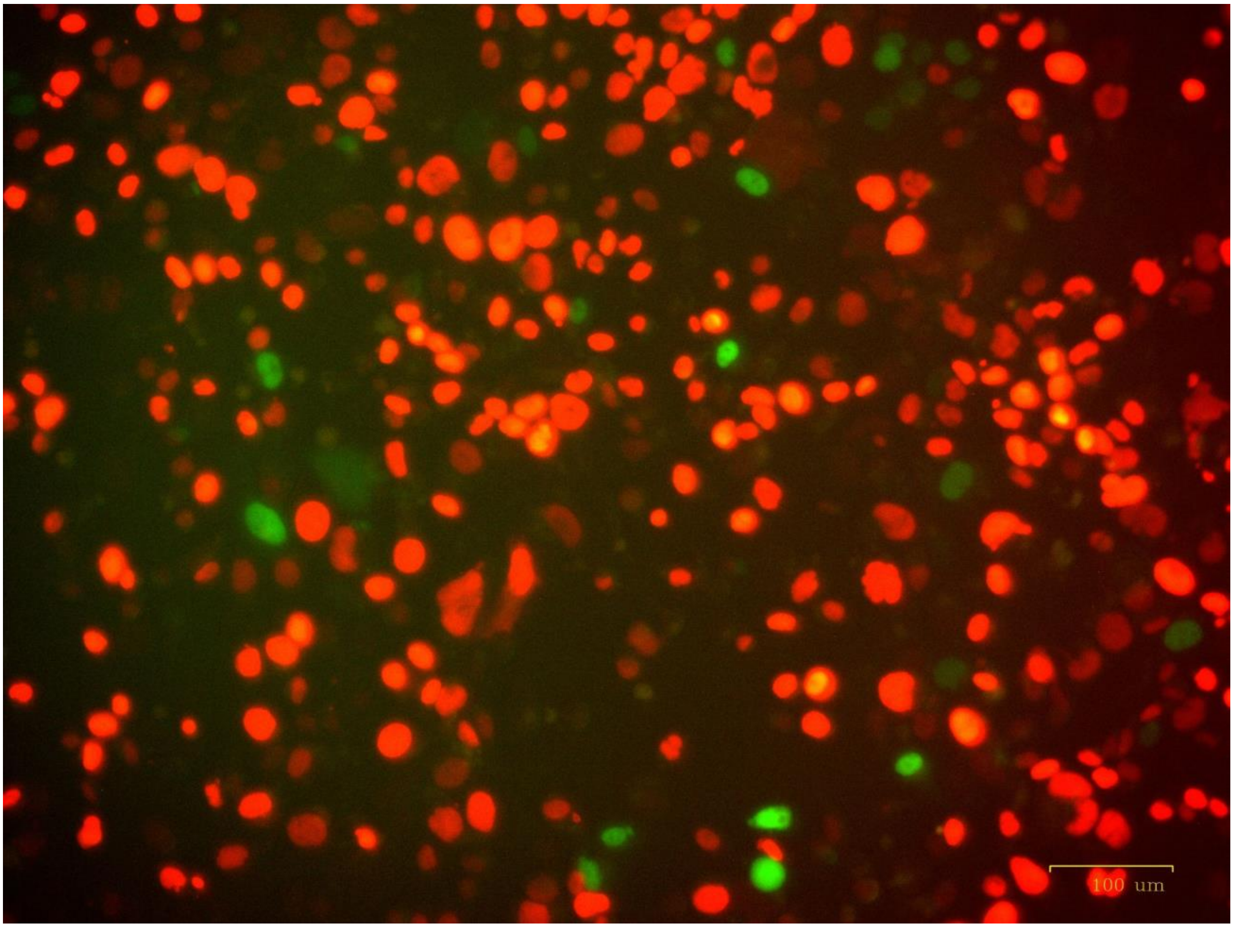
100 um

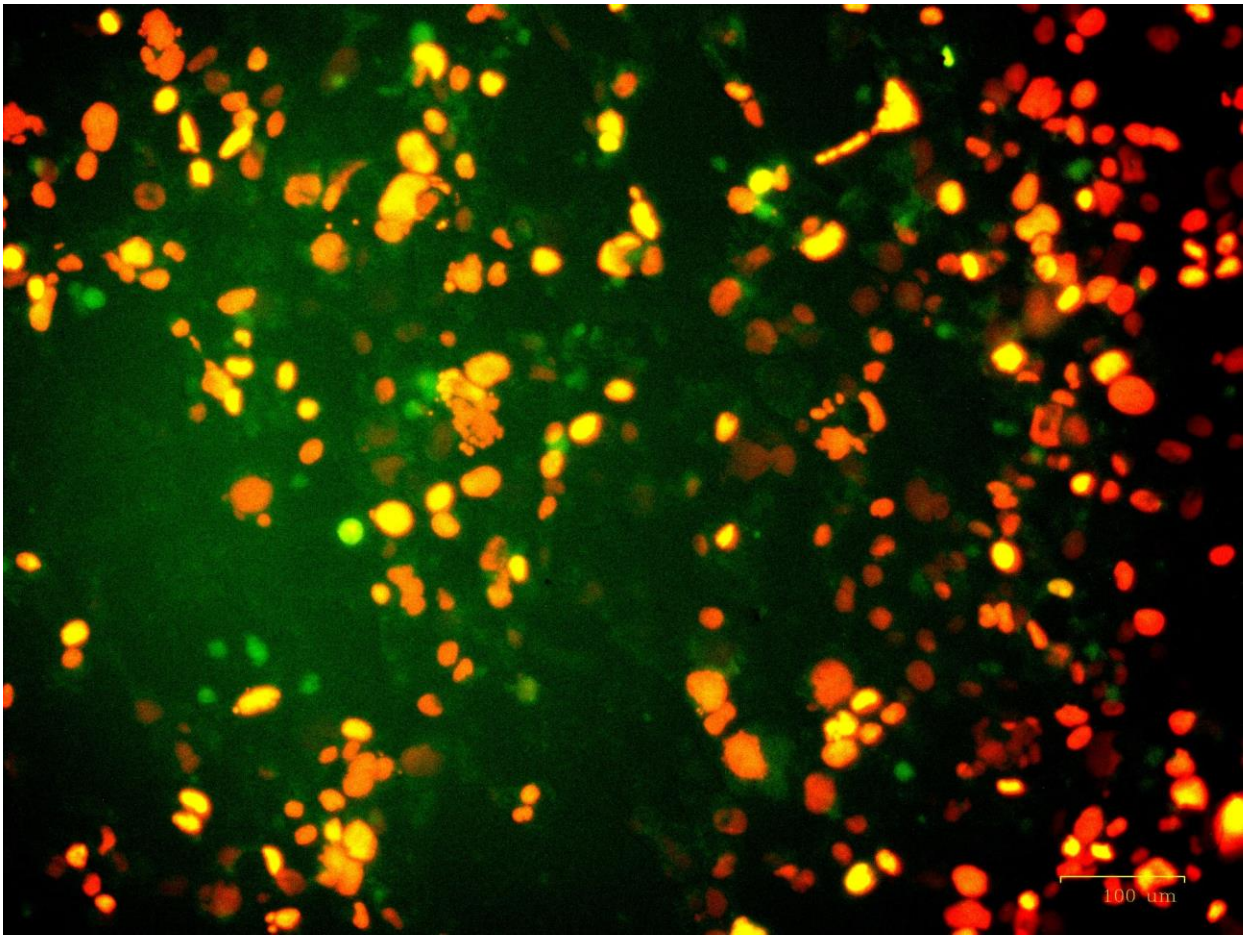
100 um

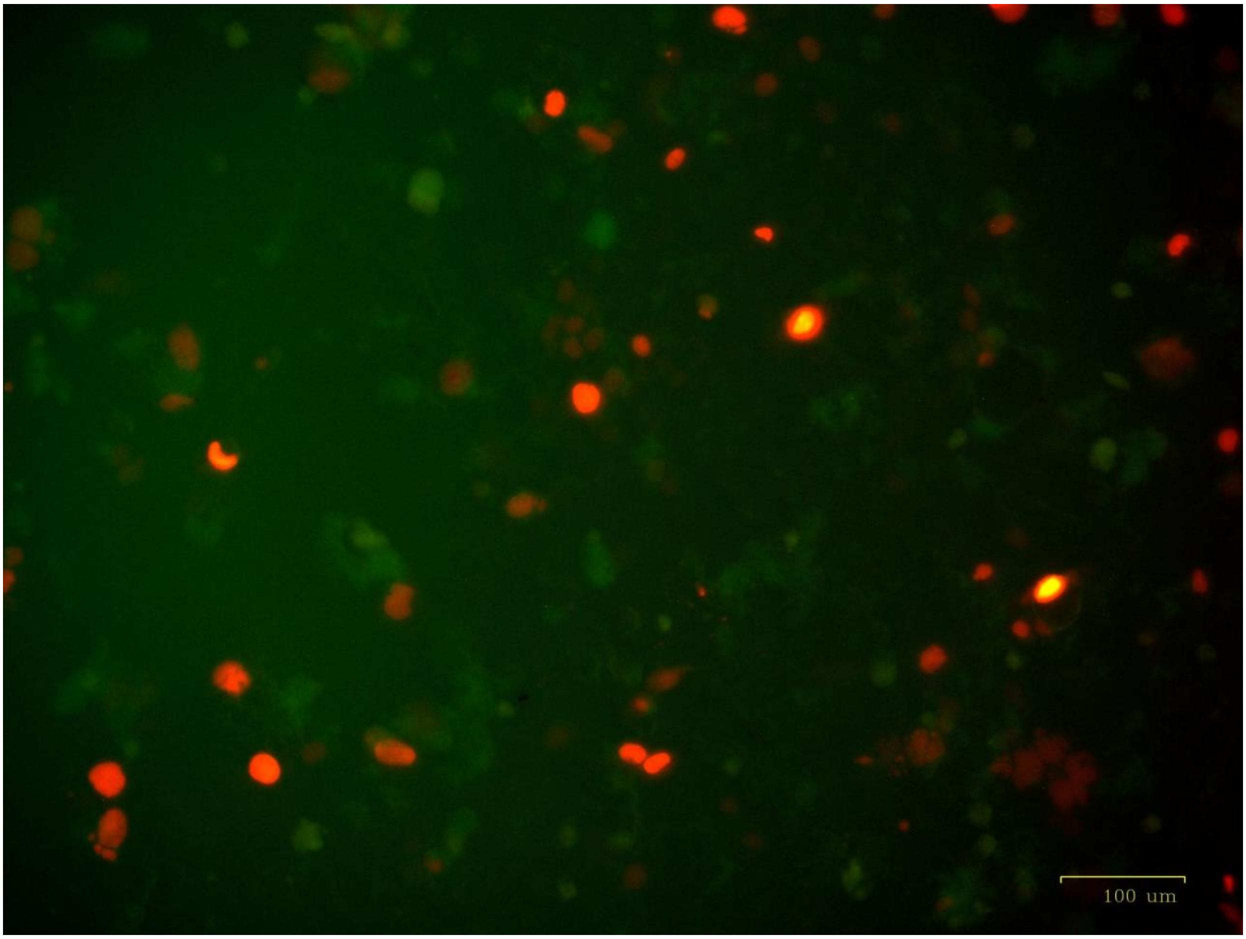
100 um

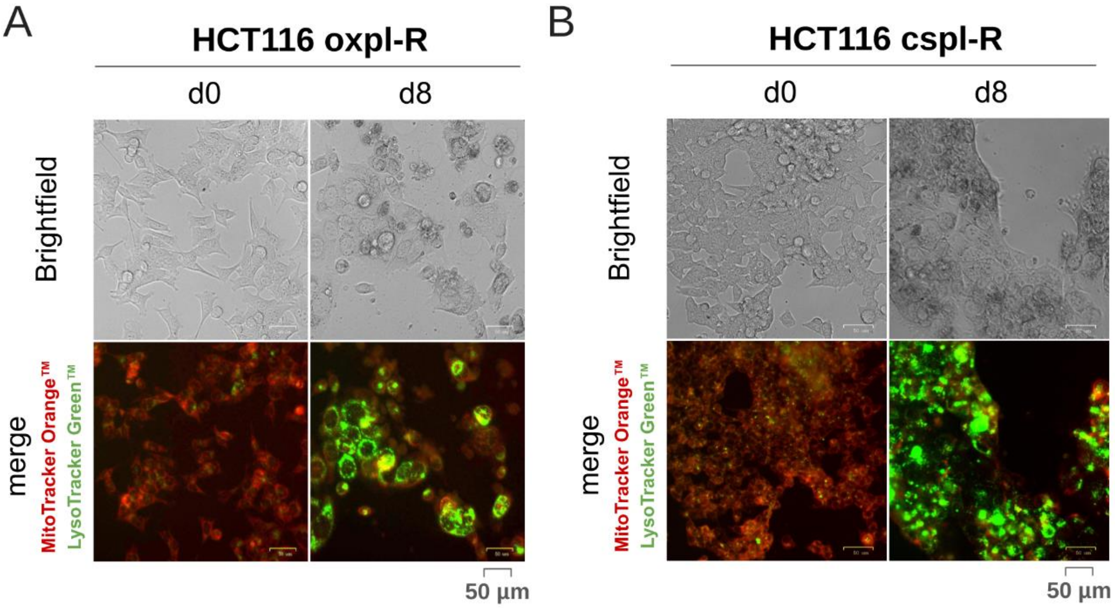

A
HCT116 oxpl-R
d0
d8
Brightfield
merge
MitoTracker Orange™
LysoTracker Green™
50 µm
B
HCT116 cspl-R
d0
d8
Brightfield
merge
MitoTracker Orange™
LysoTracker Green™
50 µm

HCT116 oxpl-R - proliferating

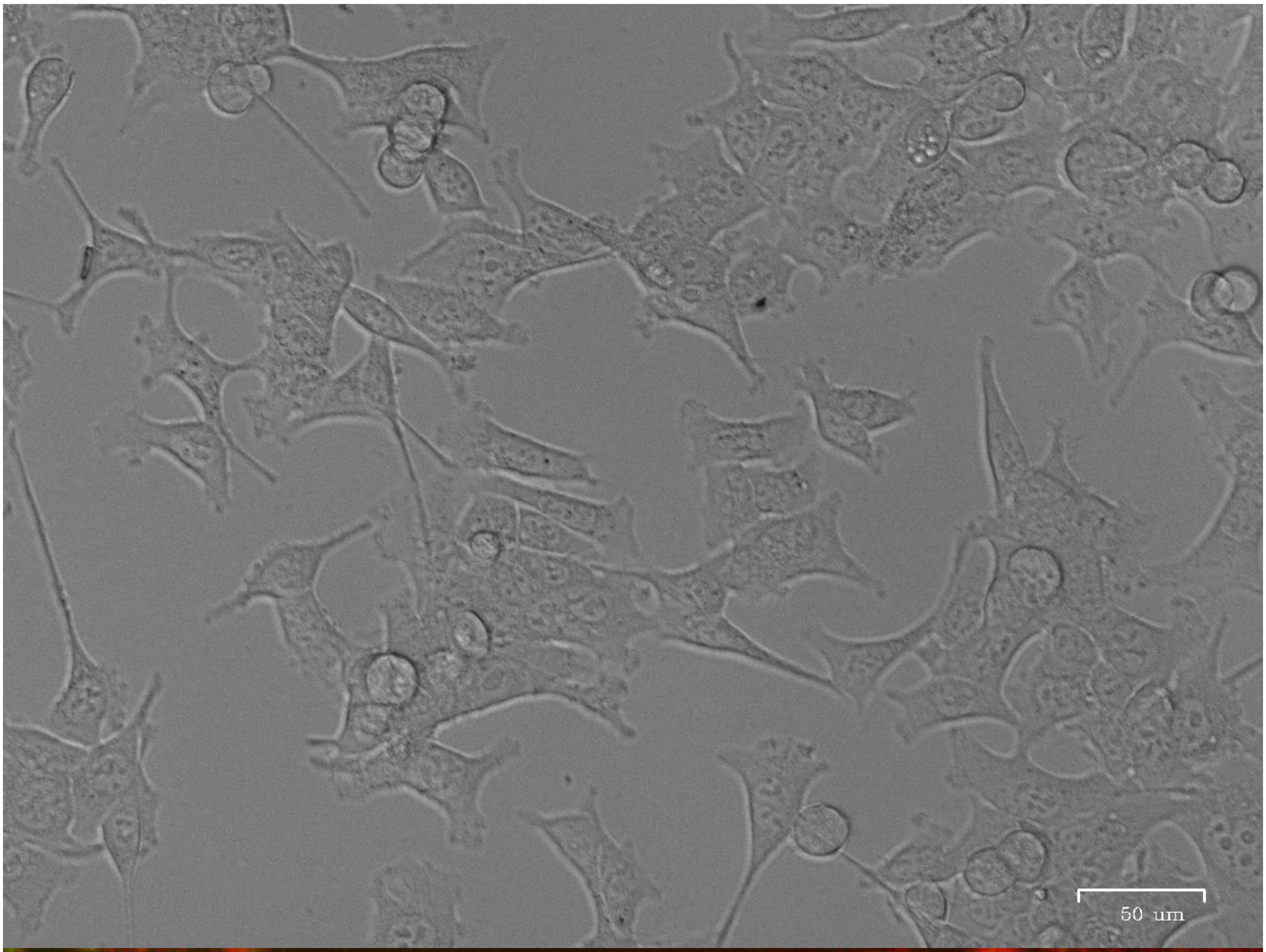

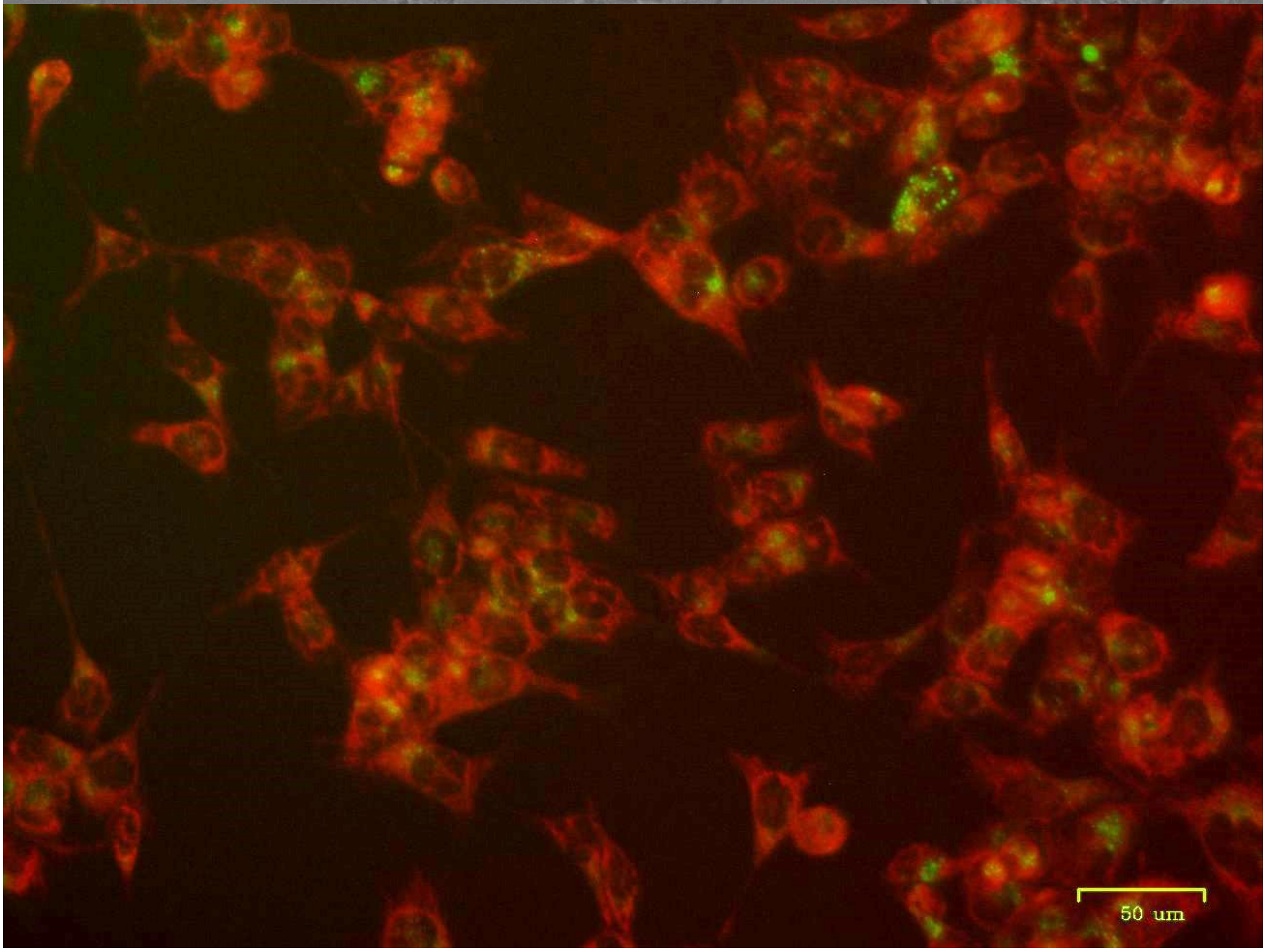

HCT116 oxpl-R - dormant

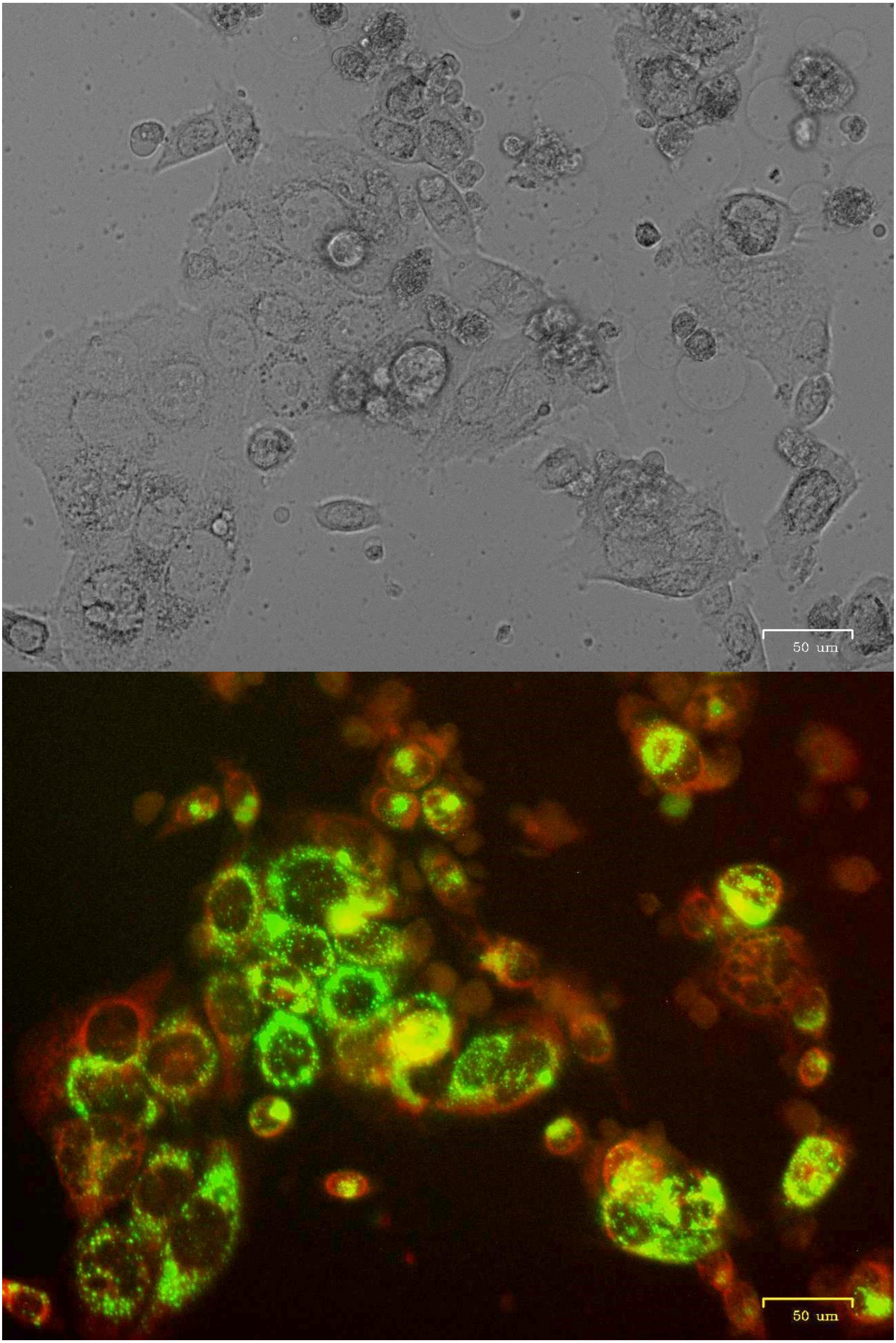

HCT116 cspII-R - proliferating

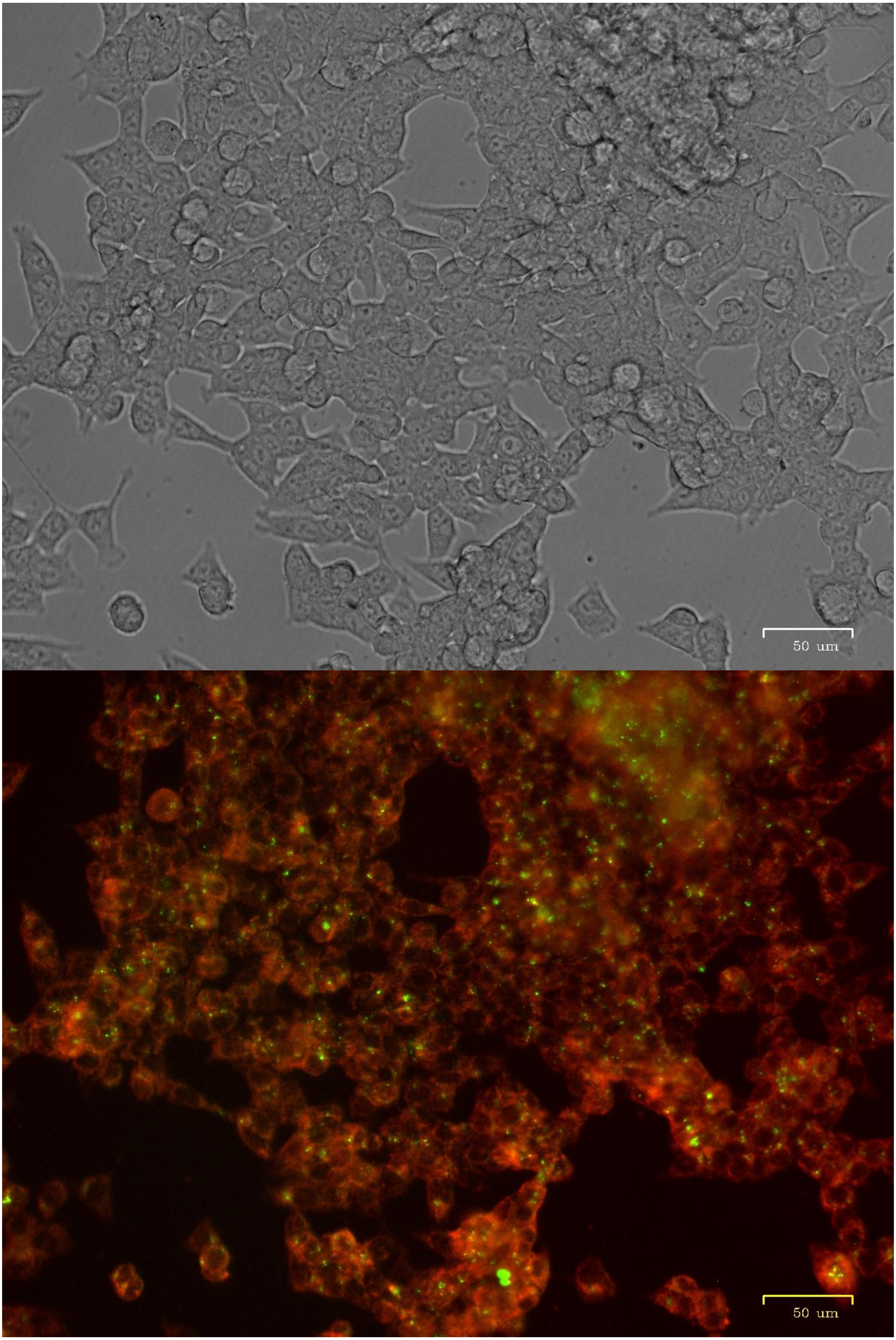

HCT116 cspll-R - dormant

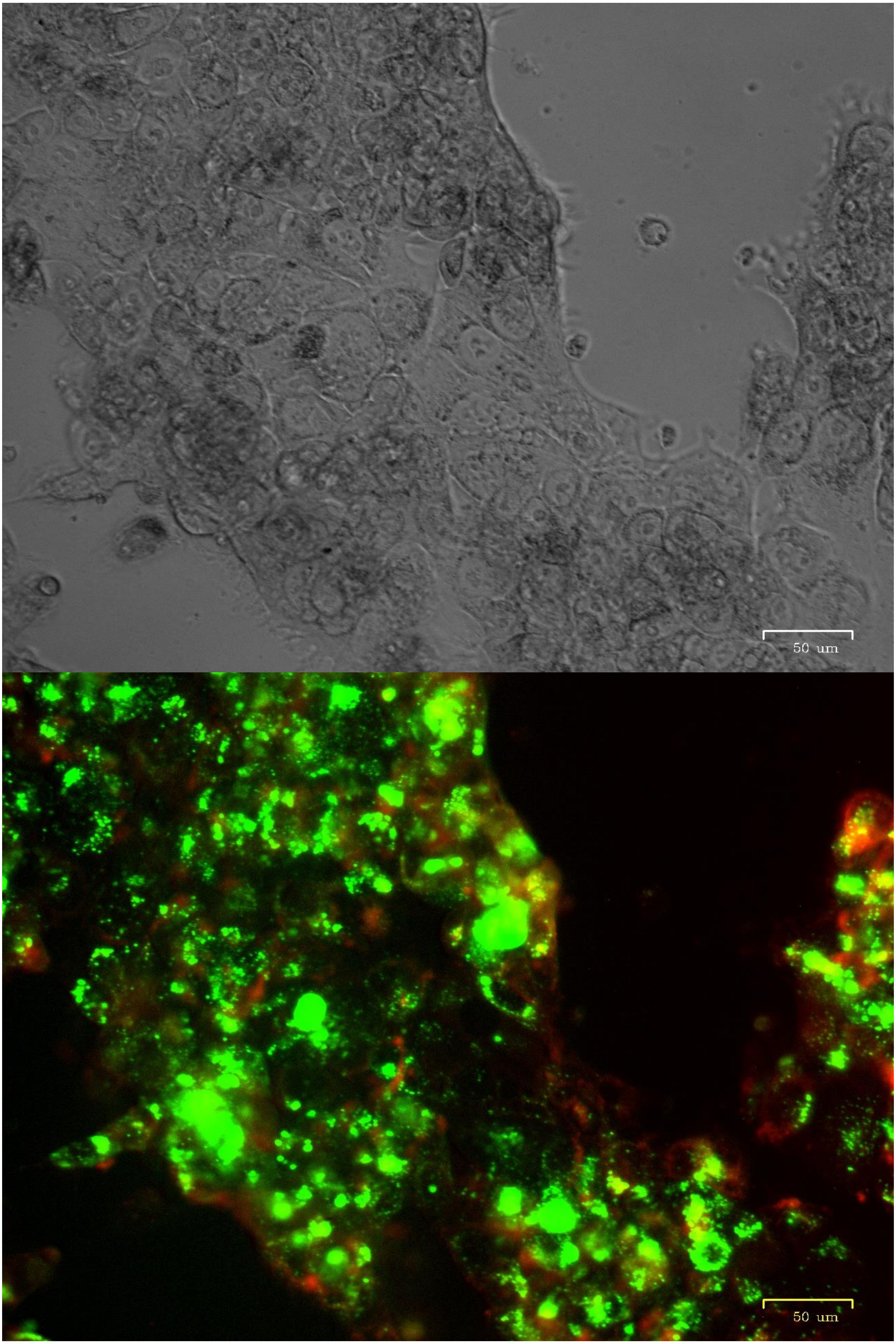

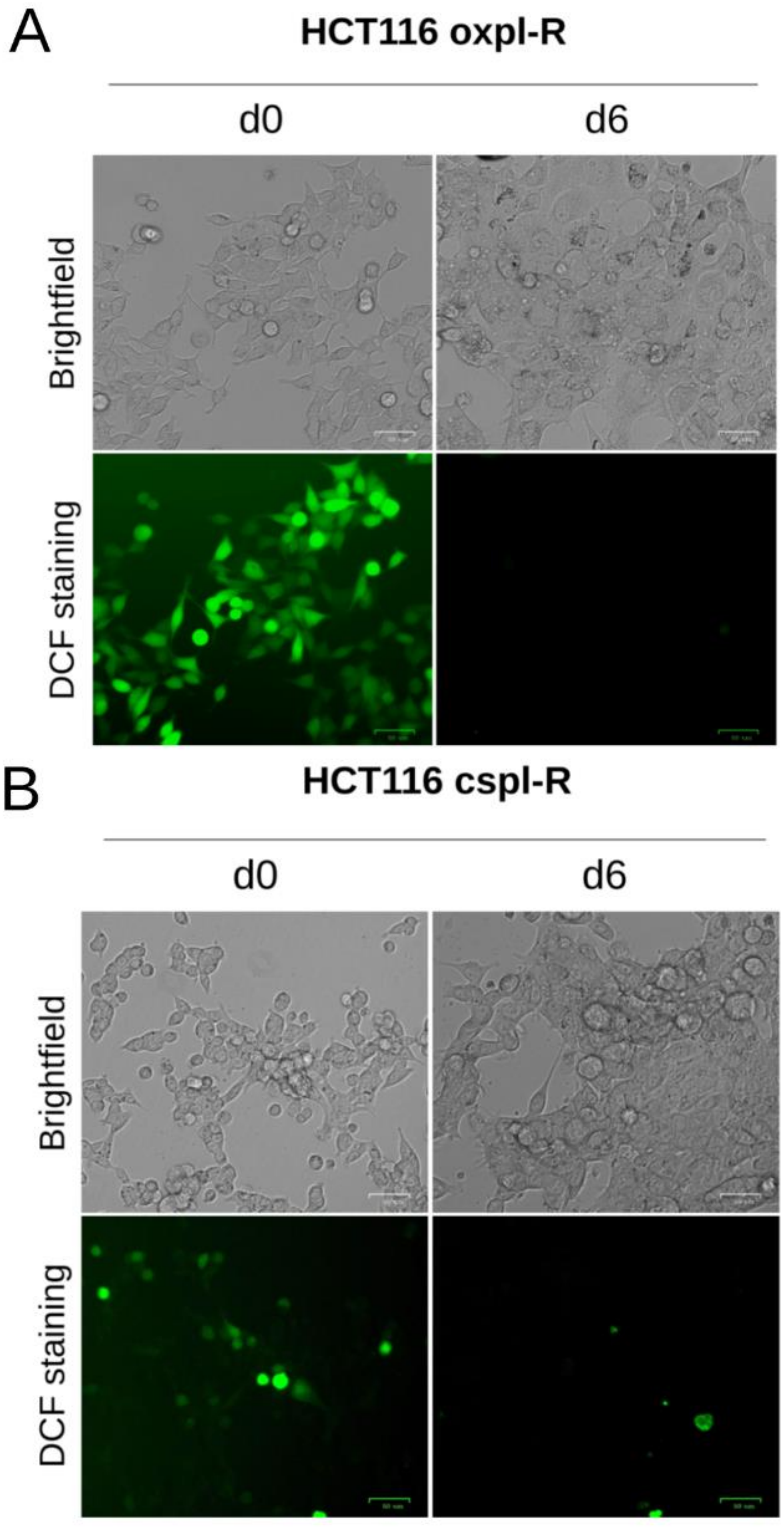
A
HCT116 oxpl-R
d0
d6
Brightfield
DCF staining
B
HCT116 cspl-R
d0
d6
Brightfield
DCF staining

HCT116 oxpl-R - proliferating

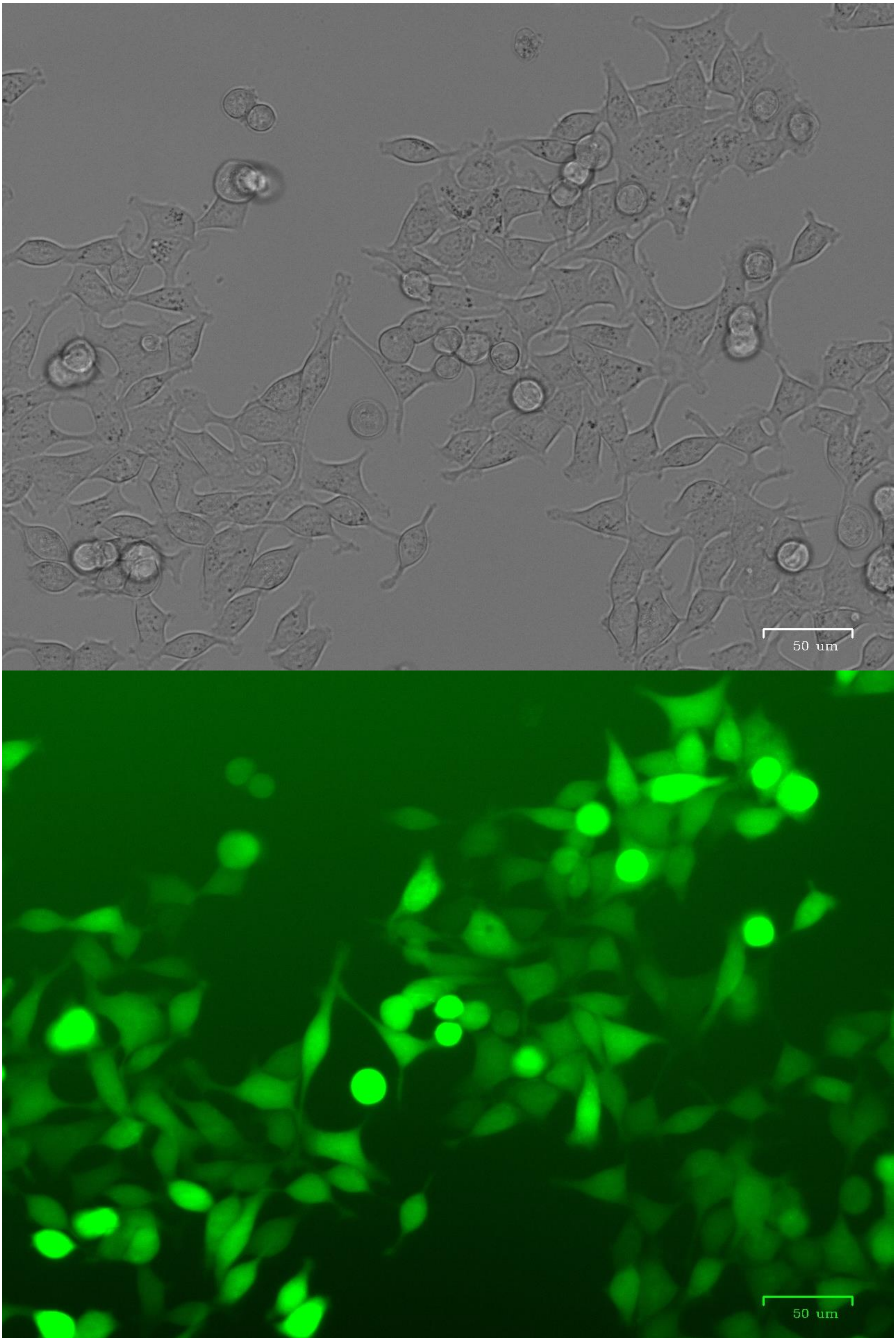

HCT116 oxpl-R - dormant

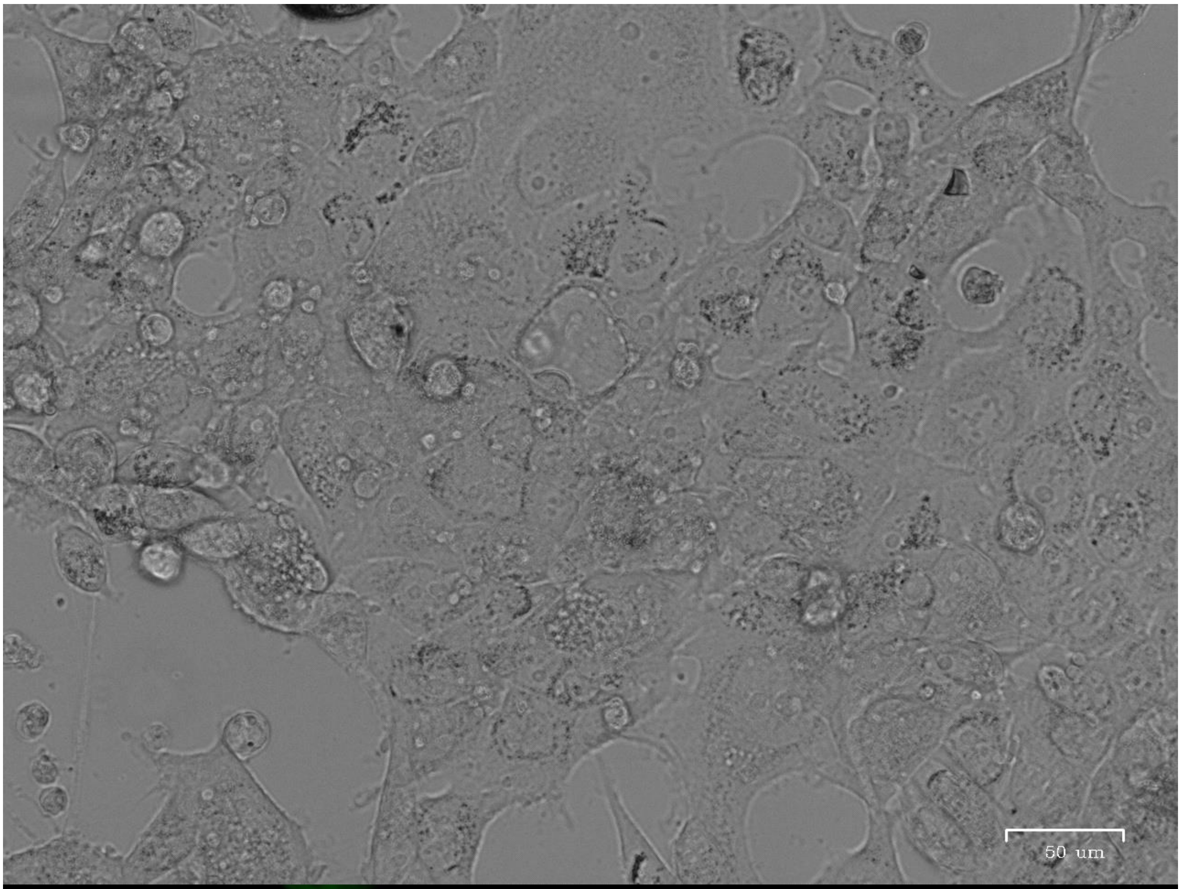

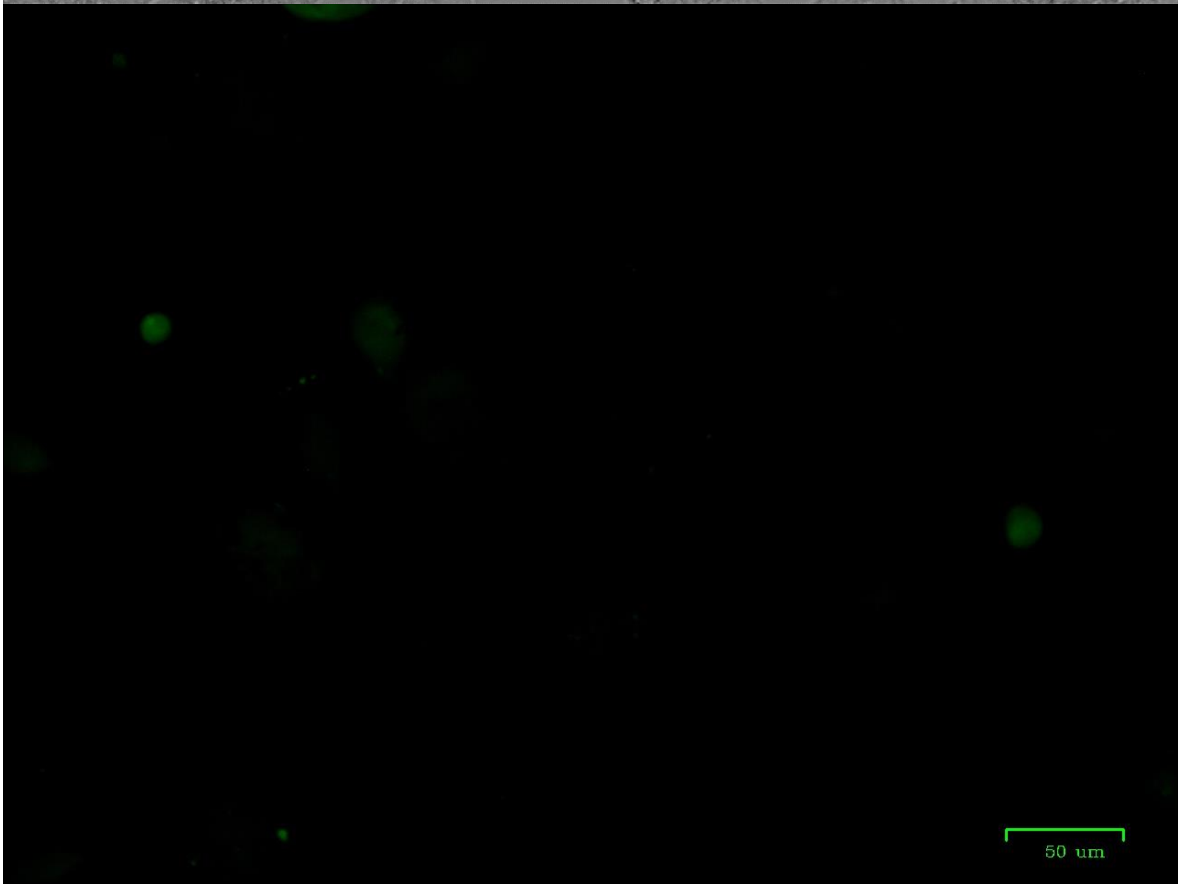

HCT116 cspII-R - proliferating

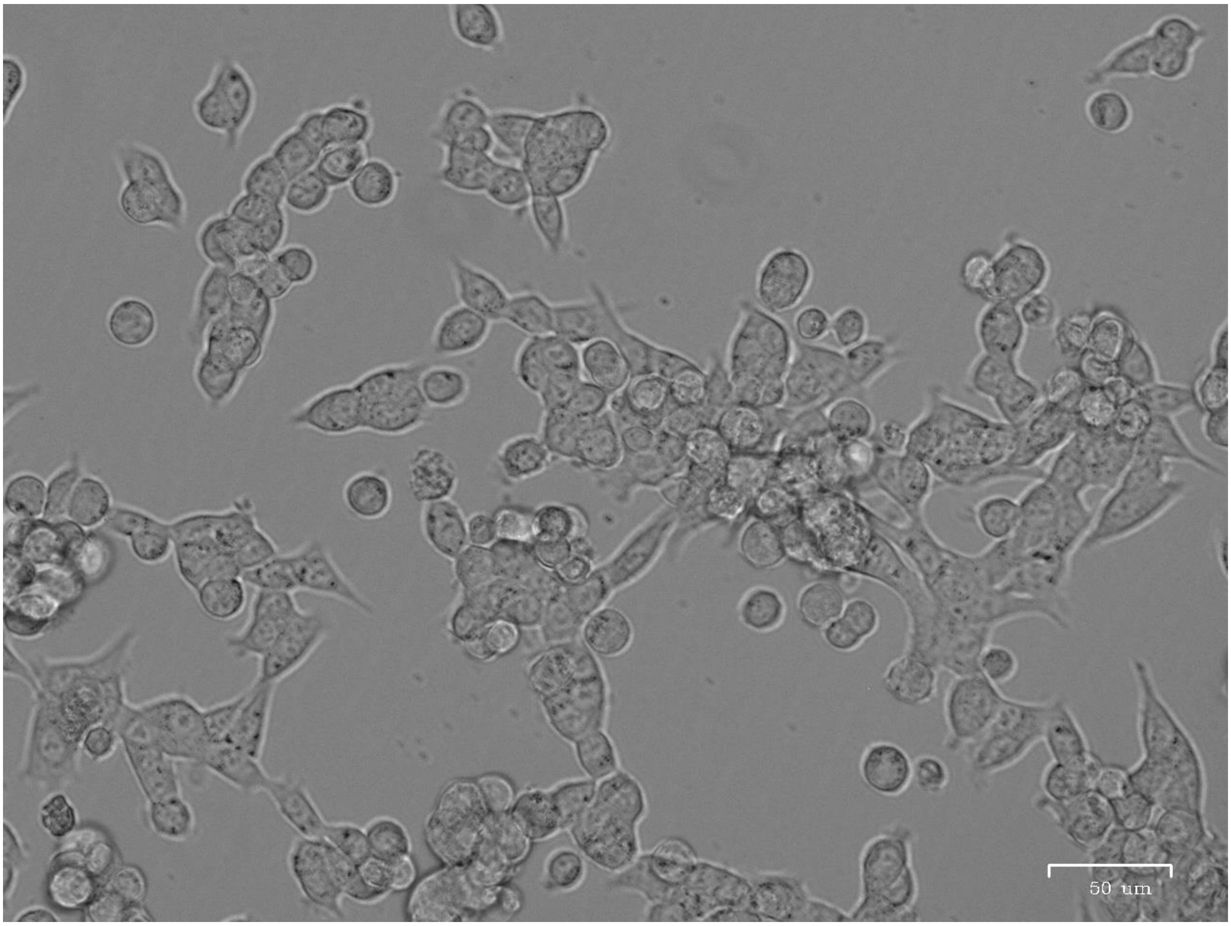

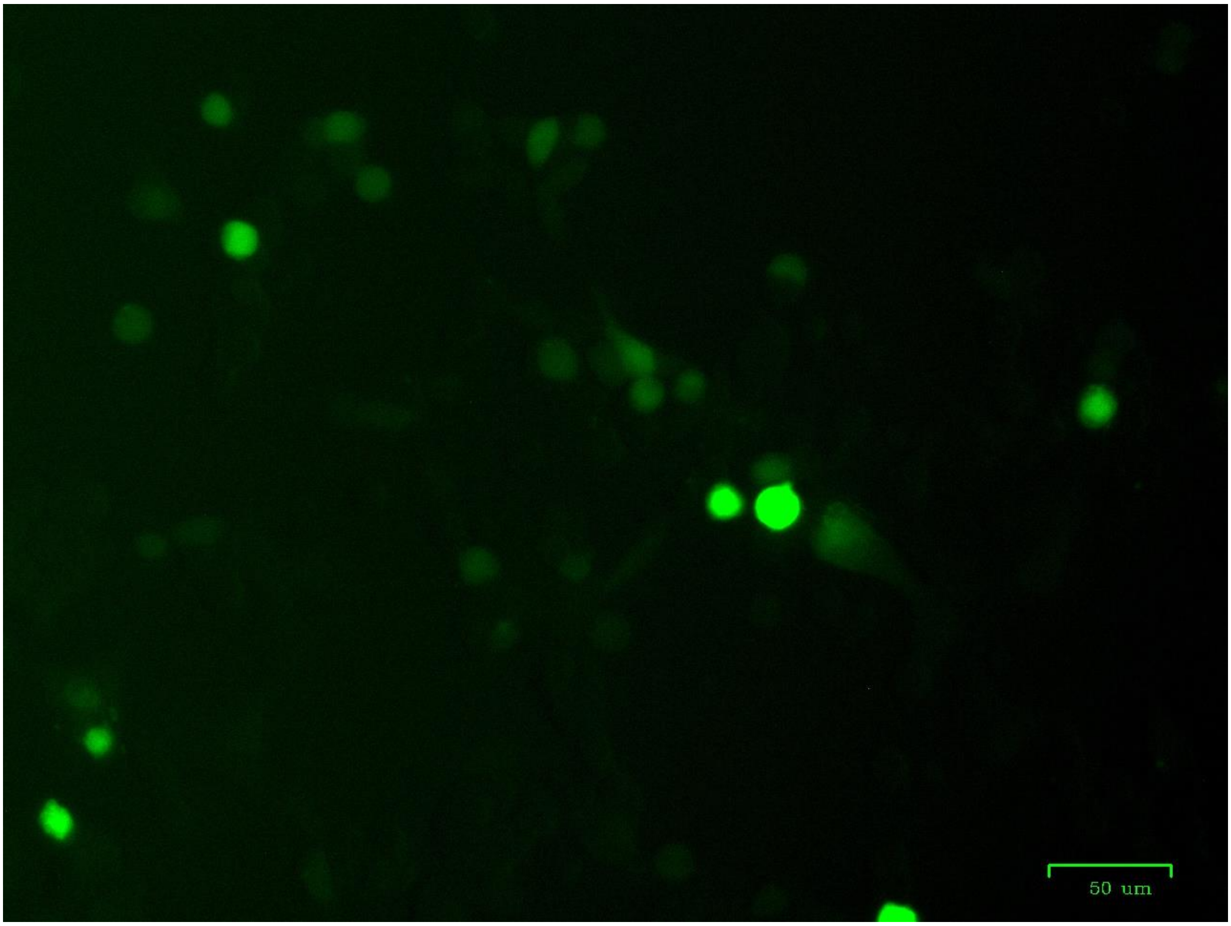

HCT116 cspll-R - dormant

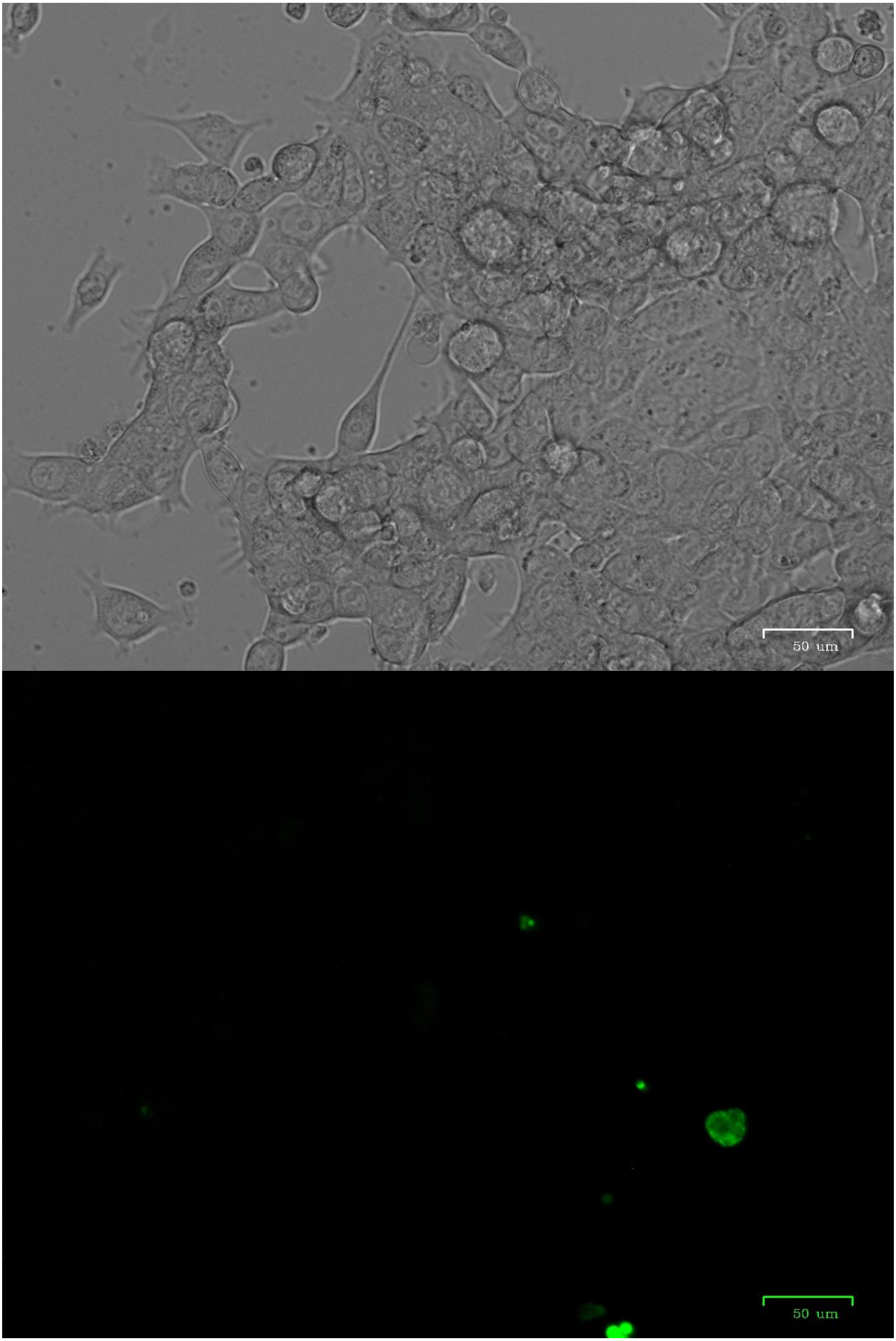

Minimal data set

qPCR Figure 2, Supplementary 3

Gene		R1	R2	R3	Cq MEAN	Cq St Dev
MKI67	d0	22,44	22,51	22,475	22,5	0,05
	d3	23,74	23,51	23,625	23,6	0,16
	d6	28,39	28,28	28,335	28,3	0,08
	d10	26,92	27,06	26,99	27,0	0,10
	d22	26,31	25,91	26,11	26,1	0,28
AURKA	d0	22,44	22,51	22,475	22,5	0,05
	d3	23,74	23,51	23,625	23,6	0,16
	d6	28,39	28,28	28,335	28,3	0,08
	d10	26,92	27,06	26,99	27,0	0,10
	d22	24,86	24,61	24,62	24,7	0,14
cyclin A	d0	23,37	23,09	23,23	23,2	0,14
	d3	26,26	25,78	26,02	26,0	0,24
	d6	26,03	25,52	25,775	25,8	0,26
	d10	27,14	26,79	26,965	27,0	0,18
	d22	26	25,5	25,75	25,8	0,25
cyclin B	d0	22,02	21,83	21,925	21,9	0,09
	d3	24,73	24,57	24,65	24,7	0,08
	d6	26,29	26,16	26,225	26,2	0,07
	d10	24,19	24,03	24,11	24,1	0,08
	d22	24,83	24,37	24,6	24,6	0,23
GAPDH	d0	27,41	32,03	29,7	29,72	3,27
	d3	28,75	29,45	29,03	29,1	0,49
	d6	26,25	28,21	27,26	27,23	1,39
	d10	27,47	24,09	25.71	25,78	2,39
	d22	27,37	28,23	27.63	27,8	0,61

Gene		R1	R2	Cq MEAN	Cq St Dev
p21Waf1	d0	28,19	27,96	28,07	0,12
	d3	24,43	24,96	24,69	0,32
	d6	23,5	23,55	23,53	0,03
	d10	24,23	23,75	23,99	0,24
	d22	26,2	27,1	26,43	0,59
oct4	d0	28,77	28,49	28,63	0,20
	d3	28,51	29,08	29,08	0,40
	d10	29,96	29,82	29,89	0,10
	d22	29,87	30,24	30,46	0,26
sox2	d0	28,77	28,49	28,63	0,20
	d3	27,45	27,4	27,43	0,04
	d10	29,96	29,82	29,89	0,10
	d22	29,5	28,49	29,00	0,71
Clusterin	d0	31,19	31,09	31,14	0,07
	d3	30,93	30,96	30,94	0,02
	d10	30,87	31,21	31,04	0,24
	d22	31,1	31,01	31,1	0,06
GAPDH	d0	17,77	17,86	17,815	0,06
	d3	19,11	19,01	19,06	0,07
	d10	18,17	17,92	18,045	0,18
	d22	18,97	18,87	18,92	0,07

qPCR Figure 3, Figure 5

Gene		R1	R2	R3	MEAN	Cq SEM
LC3	d0	30,21	30,27		30,24	0,03
LC3	d1	26,06	26,11		26,085	0,025
LC3	d4	26,48	26,8		26,64	0,16
LC3	d6	26,47	26,47		26,47	0
LC3	d12	26,1	26,26		26,18	0,08
LC3	d18	25,2	25,19		25,195	0,005
LC3	d28	30,46	30,43		30,445	0,015
LC3	d33	28,88	29,28		29,08	0,2
Beclin	d0	N/A	N/A			
Beclin	d1	30,56	30,72		30,64	0,08
Beclin	d4	31,94	31,54		31,74	0,2
Beclin	d6	32,27	32,01		32,14	0,13
Beclin	d12	31,46	31,41		31,435	0,025
Beclin	d18	31,14	31,88		31,51	0,37
Beclin	d28	N/A	N/A			
Beclin	d33	34,44	34,61		34,525	0,085
GAPDH	d0	26,31	26,58		26,445	0,135
GAPDH	d1	23,5	22,84		23,17	0,33
GAPDH	d4	24,4	23,63		24,015	0,385
GAPDH	d6	26,46	25,71		26,085	0,375
GAPDH	d12	23,77	23,01		23,39	0,38
GAPDH	d18	23,19	22,45		22,82	0,37
GAPDH	d28	28,46	27,34		27,9	0,56
GAPDH	d33	26,22	25,3		25,76	0,46

Gene		R1	R2	R3	MEAN	Cq SEM
GAPDH	d0	25,7	25,79		25,745	0,045
GAPDH	d1	25,82	25,66		25,74	0,08
GAPDH	d4	27,92	26,98		27,45	0,47
GAPDH	d6	28,11	27,51		27,81	0,3
GAPDH	d12	25,88	25,85		25,865	0,015
GAPDH	d18	26,81	26,58		26,695	0,115
GAPDH	d28	25,83	25,72		25,775	0,055
GAPDH	d33	25,2	25,09		25,145	0,055
AURKA	d0	25,52	25,82		25,67	0,15
AURKA	d1	24,99	25,81		25,4	0,41
AURKA	d4	25,7	25,71		25,705	0,005
AURKA	d6	26,88	26,66		26,77	0,11
AURKA	d12	24,8	24,92		24,86	0,06
AURKA	d18	26,82	26,73		26,775	0,045
AURKA	d28	24,87	24,8		24,835	0,035
AURKA	d33	24,14	24,01		24,075	0,065
MKI67	d0	24,21	24,37		24,29	0,08
MKI67	d1	21,84	23,46		22,65	0,81
MKI67	d4	22,66	22,39		22,525	0,135
MKI67	d6	25,29	26,18		25,735	0,445
MKI67	d12	21,89	21,91		21,9	0,01
MKI67	d18	24,62	24,75		24,685	0,065
MKI67	d28	22,39	22,58		22,485	0,095
MKI67	d33	21,45	21,42		21,435	0,015
E2F	d0	22,29	22,22		22,255	0,035
E2F	d1	22,46	22,4		22,43	0,03
E2F	d4	23,61	23,67		23,64	0,03
E2F	d6	25,09	25,04		25,065	0,025
E2F	d12	21,71	21,93		21,82	0,11
E2F	d18	24,61	24,47		24,54	0,07
E2F	d28	22,55	22,62		22,585	0,035
E2F	d33	21,94	22,06		22	0,06
Cyclin A	d0	23,51	23,56		23,535	0,025
Cyclin A	d1	22,72	22,7		22,71	0,01
Cyclin A	d4	24,04	24,14		24,09	0,05
Cyclin A	d6	25,82	25,8		25,81	0,01
Cyclin A	d12	22,49	22,53		22,51	0,02
Cyclin A	d18	24,91	25,09		25	0,09
Cyclin A	d28	22,94	23,02		22,98	0,04
Cyclin A	d33	21,15	21,53		21,34	0,19
Cyclin B	d0	23,01	23,15		23,08	0,07
Cyclin B	d1	22,51	22,44		22,475	0,035
Cyclin B	d4	23,98	24,08		24,03	0,05
Cyclin B	d6	25,1	25,16		25,13	0,03
Cyclin B	d12	23,12	23,28		23,2	0,08
Cyclin B	d18	24,87	25,14		25,005	0,135
Cyclin B	d28	22,78	22,7		22,74	0,04
Cyclin B	d33	20,8	21,07		20,935	0,135
p16	d0	24,03	24,14		24,085	0,055
p16	d1	24,08	23,63		23,855	0,225
p16	d4	25,67	25,32		25,495	0,175
p16	d6	25,39	25,14		25,265	0,125
p16	d12	24,06	23,91		23,985	0,075
p16	d18	24,84	24,91		24,875	0,035
p16	d28	24,38	25,05		24,715	0,335
p16	d33	23,31	23,46		23,385	0,075

MTT (Fig. 4)

MTT (fig. 4)		R1	R2	R3	MEAN	SD	FOLD
proliferating cells (day 0)	control	0,72	0,686	0,684	0,70	0,02	1,0
	cspl	0,697	0,655	0,681	0,68	0,02	1,0
	oxpl	0,52	0,529		0,52	0,01	0,8
	etop	0,516	0,461	0,47	0,48	0,03	0,7
	iri	0,363	0,384	0,353	0,37	0,02	0,5
	5-FU	0,361	0,339	0,342	0,35	0,01	0,5
	dxr	0,264	0,199	0,257	0,24	0,04	0,3
quiescent cells (day 6)	control	1,005	0,917	0,954	0,96	0,04	1,0
	cspl	0,779	0,949	0,981	0,90	0,11	0,9
	oxpl	0,848	0,901	0,917	0,89	0,04	0,9
	etop	0,883	1,132	1,071	1,03	0,13	1,1
	iri	0,732	0,727	0,754	0,74	0,01	0,8
	5-FU	0,742	0,767	0,715	0,74	0,03	0,8
	dxr	0,892	0,825	0,902	0,87	0,04	0,9

FUCCI (Fig 2)

Signal	Point	MEAN
**mKO2-Cdt1**	0	9,62
**mKO2-Cdt1**	2	52,08
**mKO2-Cdt1**	4	97,49
**mKO2-Cdt1**	6	81,08
**mKO2-Cdt1**	9	81,48
**mKO2-Cdt1**	13	37,97
Clover-geminin	0	90,38
Clover-geminin	2	47,92
Clover-geminin	4	2,51
Clover-geminin	6	18,92
Clover-geminin	9	18,52
Clover-geminin	13	62,03

p-p38 : p-ERK ratio (Fig 3)

	**p-p38**		**pERK**	
	MEAN	SEM	MEAN	SEM
0	50	4	50	4
1	77,807	7	22,193	7
4	63,708	9	36,292	9
18	88,229	5	11,771	5
28	81,18	7	18,82	7
33	51,502	5	48,498	5

raw band density (MEAN)

	**p-p38**	**pERK**
0	1315	1702
1	1148	423
4	570	420
18	33866	5848
28	12896	3870
33	6046	7370

qPCR Figure 6

Gene		R1	R2	R3	MEAN	Cq SEM
VIM	d0	N/A	N/A			
VIM	d1	N/A	N/A			
VIM	d4	N/A	N/A			
VIM	d6	32,46	31,84		32,15	0,31
VIM	d12	31,3	30,76		31,03	0,27
VIM	d18	32,61	32,35		32,48	0,13
VIM	d28	N/A	N/A			
VIM	d33					
N-Cadherin	d0	29,48	29,81		29,645	0,165
N-Cadherin	d1	29,48	29,76		29,62	0,14
N-Cadherin	d4	29,49	29,65		29,57	0,08
N-Cadherin	d6	29,26	29,15		29,205	0,055
N-Cadherin	d12	29,5	29,79		29,645	0,145
N-Cadherin	d18	29,54	29,63		29,585	0,045
N-Cadherin	d28	29,48	29,23		29,355	0,125
N-Cadherin	d33	29,27	29,1		29,185	0,085
E-Cadherin	d0	25,26	25,08		25,17	0,09
E-Cadherin	d1	24,09	24,07		24,08	0,01
E-Cadherin	d4	23	23,19		23,095	0,095
E-Cadherin	d6	23,22	23,72		23,47	0,25
E-Cadherin	d12	22,81	22,8		22,805	0,005
E-Cadherin	d18	23,04	23,21		23,125	0,085
E-Cadherin	d28	23,72	23,58		23,65	0,07
E-Cadherin	d33	23,26	23,49		23,375	0,115
TWIST	d0	35,32	35,07		35,195	0,125
TWIST	d1	33,81	34,12		33,965	0,155
TWIST	d4	34,92	34,96		34,94	0,02
TWIST	d6	33,41	33,92		33,665	0,255
TWIST	d12	33,26	33,51		33,385	0,125
TWIST	d18	34,23	33,06		33,645	0,585
TWIST	d28	33,43	33,51		33,47	0,04
TWIST	d33	34,18	33,65		33,915	0,265

Gene		R1	R2	R3	MEAN	Cq SEM
OCT4	d0	28,28	28,34		28,31	0,03
OCT4	d1	27,25	27,2		27,225	0,025
OCT4	d4	26,04	26,26		26,15	0,11
OCT4	d6	26,28	26,25		26,265	0,015
OCT4	d12	25,12	25,02		25,07	0,05
OCT4	d18	26,12	26,12		26,12	0
OCT4	d28	26,36	26,45		26,405	0,045
OCT4	d33	26,1	26,14		26,12	0,02
SOX2	d0	27,58	27,98		27,78	0,2
SOX2	d1	27,5	28,05		27,775	0,275
SOX2	d4	27,13	27,38		27,255	0,125
SOX2	d6	27,03	27,12		27,075	0,045
SOX2	d12	26,27	26,33		26,3	0,03
SOX2	d18	27,05	26,99		27,02	0,03
SOX2	d28	27,22	27,15		27,185	0,035
SOX2	d33	27,11	27,09		27,1	0,01
NANOG	d0	N/A	N/A			
NANOG	d1	N/A	N/A			
NANOG	d4	32,95	32,42		32,685	0,265
NANOG	d6	32,94	32,38		32,66	0,28
NANOG	d12	31,19	31,11		31,15	0,04
NANOG	d18	32,35	32,07		32,21	0,14
NANOG	d28	N/A	N/A			
NANOG	d33	32,54	32,4		32,47	0,07
CLU	d0	24,45	24,48		24,465	0,015
CLU	d1	24,72	24,65		24,685	0,035
CLU	d4	24,34	24,53		24,435	0,095
CLU	d6	23,96	24,1		24,03	0,07
CLU	d12	23,13	23,15		23,14	0,01
CLU	d18	23,16	23,33		23,245	0,085
CLU	d28	23,83	23,81		23,82	0,01
CLU	d33	23,35	23,43		23,39	0,04
CD44	d0	37,05	36,15		36,6	0,45
CD44	d1	33,04	33,85		33,445	0,405
CD44	d4	32,39	32,24		32,315	0,075
CD44	d6	34,69	33,7		34,195	0,495
CD44	d12	32,55	32,02		32,285	0,265
CD44	d18	31,39	31,06		31,225	0,165
CD44	d28	35,76	37,16		36,46	0,7
CD44	d33	36,35	35,38		35,865	0,485

Target	Sample	1	2	Cq Mean	Cq Std. Dev
GAPDH	iPSC	18,07	17,83	17,95	0,170
GAPDH	HCT116/C d6	20,05	20,1	20,075	0,035
cd44	iPSC	28,67	28,71	28,69	0,028
cd44	HCT116/C d6	27	26,86	26,93	0,099
clusterin	iPSC	23,21	23,21	23,21	0,000
clusterin	HCT116/C d6	25,07	25,03	25,05	0,028
oct4	iPSC	21,33	21,42	21,375	0,064
oct4	HCT116/C d6	34,4	34,17	34,285	0,163
oct4	HCT116/C d6	33,32	33,99	33,655	0,474
oct4	iPSC	21,05	21,14	21,095	0,064
Nanog	HCT116/C d6	31,62	31,62	31,62	0,000
Nanog	iPSC	26,47	26,58	26,525	0,078
sox2	HCT116/C d6	31,28	31,31	31,295	0,021
sox2	iPSC	24,7	25,02	24,86	0,226

Gene		R1	R2	R3	MEAN	Cq SEM
GAPDH	d0	25,7	25,79		25,745	0,045
GAPDH	d1	25,82	25,66		25,74	0,08
GAPDH	d4	27,92	26,98		27,45	0,47
GAPDH	d6	28,11	27,51		27,81	0,3
GAPDH	d12	25,88	25,85		25,865	0,015
GAPDH	d18	26,81	26,58		26,695	0,115
GAPDH	d28	25,83	25,72		25,775	0,055
GAPDH	d33	25,2	25,09		25,145	0,055

**Sox2**	EXP1 FOLD	EXP2 FOLD	EXP3 FOLD	MEAN	sd	se
d0	1	1	1,00	1	0	0,0
d1	2,8	1,5	2,23	2,2	0,6	0,4
d4	6,1	2,4	2,72	3,7	2,0	1,2
d6	16,9	3,7	5,39	8,7	7,2	4,2
d12	6,9	3,1	2,32	4,1	2,4	1,4
d18	9,1	2,3	2,72	4,7	3,8	2,2
d28	2,9	1,8	2,01	2,2	0,6	0,3
d33	2,8	1,3	1,29	1,8	0,9	0,5
d42		1,4	1,41	1,4	0,0	0,0
**oct4**	EXP1 FOLD	EXP2 FOLD	EXP3 FOLD	MEAN	sd	se
d0	1	1	1,00	1	0	0,0
d1	3,8	3,2	4,10	3,7	0,4	0,3
d4	10,0	7,5	9,09	8,8	1,3	0,7
d6	18,4	9,3	14,27	14,0	4,6	2,6
d12	17,0	10,5	12,30	13,2	3,3	1,9
d18	7,8	6,1	8,25	7,4	1,1	0,6
d28	5,0	4,4	5,84	5,1	0,7	0,4
d33	4,7	3,6	4,87	4,4	0,7	0,4
d42		4,6	5,72	5,2	0,8	0,5
**nanog**	EXP1 FOLD	EXP2 FOLD	EXP3 FOLD	MEAN	sd	se
d0	0	0,00	0	0	0	0,0
d1	2,4	1,29	1,9	0,8	0,6	0,3
d4	3,3	2,90	3,1	0,3	0,2	0,1
d6	4,7	4,50	4,6	0,1	0,1	0,1
d12	2,7	3,78	3,3	0,7	0,5	0,3
d18	0	3,20	1,6	2,3	1,6	0,9
d28	1,3	1,54	1,4	0,1	0,1	0,1
d33	2,0	1,27	1,6	0,5	0,4	0,2
d42	3,6	2,22	2,9	1,0	0,7	0,4

DCF staining (Fig 7)

	Mean FITC-A	rSD FITC-A
HCT116 cspl-R plf	12761,9	6133,4
HCT116 cspl-R dorm	10370,9	3219,3
HCT116 oxpl-R plf	83132,0	44012,1
HCT116 oxpl-R dorm	23289,6	8712,8

MTT (Fig 8)

HCT116 cspl-R			r1	r2	r3	r4	MEAN	FOLD
		ctrl mean	0,638				0,64	1,0
d3	A	25uM 6h	0,391	0,42	0,43	0,477	0,43	0,7
	B	25uM 24h	0,392	0,4	0,34	0,394	0,38	0,6
	C	75uM 6h	0,158	0,124	0,055		0,11	0,2
	D	75uM 24h	0,102	0,099	0,049		0,08	0,1
d6	A	25uM 6h	0,91				0,91	1,4
	B	25uM 24h	0,633	0,703	0,591	0,623	0,64	1,0
	C	75uM 6h	0,235		0,255	0,133	0,21	0,3
	D	75uM 24h	0,083	0,123	0,095		0,10	0,2
d10	A	25uM 6h	0,531	0,536	0,655		0,57	0,9
	B	25uM 24h	0,617	0,407	0,473		0,50	0,8
	C	75uM 6h	0,339	0,194			0,27	0,4
	D	75uM 24h	0,07	0,19			0,13	0,2
d18	A	25uM 6h	1,046	1,082	1,201	0,92	1,06	1,7
	B	25uM 24h	0,814	0,837	0,857		0,84	1,3
	C	75uM 6h	0,51				0,51	0,8
	D	75uM 24h	0,064	0,06	0,074		0,07	0,1

HCT116 cspl-R			r1	r2	r3	r4	MEAN	FOLD
		ctrl mean	0,24				0,24	1,0
	ctrl	-	0,964				0,964	1,6
d3	A	25uM 6h	0,783	0,826	0,748		0,79	1,3
	B	25uM 24h	0,671	0,635	0,6		0,64	1,1
	C	75uM 6h	0,452	0,384	0,455		0,43	0,7
	D	75uM 24h	0,185	0,168			0,18	0,3
d6	A	25uM 6h	0,42	0,521	0,5		0,48	0,8
	B	25uM 24h	0,312	0,37	0,339		0,34	0,6
	C	75uM 6h	0,328	0,292	0,309		0,31	0,5
	D	75uM 24h	0,122	0,119			0,12	0,2

HCT116 oxpl-R			r1	r2	r3	MEAN	FOLD	SD
		ctrl mean	0,407			0,41	1,0	
d3	A	50uM 6h	0,285	0,227	0,248	0,25	0,6	0,03
	B	50uM 24h	0,22	0,222	0,208	0,22	0,5	0,01
	C	150uM 6h	0,197	0,208	0,206	0,20	0,5	0,01
	D	150uM 24h	0,158	0,198		0,18	0,4	0,03
d6	A	50uM 6h	0,808	0,799	0,702	0,77	1,9	0,06
	B	50uM 24h	0,37	0,342	0,349	0,35	0,9	0,01
	C	150uM 6h	0,467	0,434	0,524	0,48	1,2	0,05
	D	150uM 24h	0,262	0,298		0,28	0,7	0,03
d10	A	50uM 6h	1,001	0,971	1,0255	1,00	2,5	0,03
	B	50uM 24h	0,537	0,308	0,299	0,38	0,9	0,13
	C	150uM 6h	0,488			0,49	1,2	0,10
	D	150uM 24h	0,208	0,244		0,23	0,6	0,03
d14	A	50uM 6h	monolayer, hypoxia					
	B	50uM 24h	0,352	0,529	0,458	0,45	1,1	0,09
	C	150uM 6h	0,466	0,554	0,661	0,56	1,4	0,10
	D	150uM 24h	0,252	0,202	0,288	0,25	0,6	0,04
d18	A	50uM 6h						
	B	50uM 24h	0,818	0,797	0,613	0,74	1,8	0,11
	C	150uM 6h	0,848	0,815	0,838	0,83	2,0	0,02
	D	150uM 24h	0,151	0,141		0,15	0,4	0,01

MTT (Fig S1)

			R1	R2	R3	MEAN	SD	FOLD
HCT116	ctrl	48	1,037	0,904	1,015	0,99	0,07	1,00
	cisp 25 uM	48	0,43	0,376	0,478	0,43	0,05	0,43
	oxpl 10 uM	48	0,407	0,367	0,366	0,38	0,02	0,39
HCT116 cspl-R	ctrl	48	1,029	0,954		0,99	0,05	1,00
	cisp 25 uM	48	0,902	0,856		0,88	0,03	0,89
	oxpl 10 uM	48	0,455	0,403		0,43	0,04	0,43
HCT116 oxpl-R	ctrl	48	0,655	0,706		0,68	0,04	1,00
	cisp 25 uM	48	0,577	0,582		0,58	0,00	0,85
	oxpl 10 uM	48	0,471	0,459	0,422	0,45	0,03	0,66

FLOW CYTOMETRY (Fig S1)

Sub G1 - apoptotic cells, %		
Cells	ctrl	cisp 48h
HCT116	4,4	44,67
HCT116/C	2,18	10,67

Sub G1 - apoptotic cells, %		
Cells	ctrl	oxpl 48h
HCT116	1,52	12,95
HCT116/O	1,12	1,7

Cloning efficiency, % (Fig S1)

c	Area Percent						
HCT116	R1	R2	R3	MEAN	StDev	FOLD	StDev
ctrl	21,17	18,54	16,23	18,65	2,47	100	13,26
Oxpl	2,03	0,78	0,58	1,13	0,79	6,06	4,21

HCT116 oxpl-R	R1	R2	R3	MEAN	StDev	FOLD	StDev
ctrl	22,86	32,06	28,05	27,66	4,61	100	16,68
Oxpl	27,39	21,67	19,54	22,87	4,06	83	14,68
