# Supplementary material for: An in vitro tumor recurrence model based on platinum-resistant colon cancer cells as a research tool for studying cancer cell dormancy
Source: PLoS One. 2025 Oct 8;20(10):e0333671. doi: 10.1371/journal.pone.0333671 (PMC12507233; doi:10.1371/journal.pone.0333671)
Supplement: Attachment — Submitted filename: Response to Reviewers.pdf [file pone.0333671.s006.pdf]

PLOS One

REBUTTAL LETTER

Dear Li Yang,

We would like to thank you and the reviewers for the thorough evaluation of our manuscript and the insightful comments provided. We have carefully considered all the feedback and made substantial revisions to improve the overall quality of the paper. In particular, we have significantly enhanced the language, correcting grammatical issues and removing stylistic errors to ensure greater clarity and readability.

During the revision process, we identified an error in the MTT assay graphs on Figure 4 where two data point labels were switched; this has now been corrected to accurately reflect the results. Additionally, we replaced one of the photographs in panel of Figure 1 with a more representative image, and to ensure transparency and completeness, we have included the full, uncropped images in the supplementary materials.

At the reviewers' request, we expanded the discussion section and the figure legends, and we also improved the quality of the illustrative materials to better highlight key findings. Furthermore, we conducted additional experiments to extend the data presented in Figure 7, and these have been included along with supplementary experiments demonstrating the cells' stability in response to platinum-based drugs. Moreover, we supplemented Figures 5 and 8 with results obtained from HCT116 cspl-R cells.

We have also revised the formatting of the manuscript and figures to comply with the requirements of PLOS ONE. Raw blot images attached as supporting Information file. As for the funding statement, we added the following, as recommended: "The funders had no role in study design, data collection and analysis, decision to publish, or preparation of the manuscript." Text changes throughout the manuscript have been highlighted in yellow. Additionally, we have prepared detailed point-by-point responses to each reviewer's comments below.

Response to reviewer 1

We sincerely thank the reviewer for their interest in our manuscript and for the thorough evaluation. Your insightful comments have helped us to improve the quality and informativeness of the article. Below, we will address your questions point by point:

1. The authors did not directly compare drug-resistance cells with parental cells, such as IC50 shifts, survival curves or dose response study.

- Since this article is mostly focused on the dormancy and recurrence model, we didn't include details on developing drug resistance and characterization of the cell lines. HCT116 cspl-R was characterized in a distinct article (10.1134/S1990519X22060037) published earlier, and HCT116 oxpl-R - in another article to be published this year. However, we recognize the importance of this data for dormancy modeling.

Therefore, we have included the changes in IC50 as drug resistance develops, along with dose-dependent survival curves for both parental cells and cspl-R/oxpl-R cells, in the Supplementary Materials (S1).

2. The authors did not perform short-term stability studies of the new cells, such as colony formation study and apoptosis studies when applied to platinum drugs.

- Regarding the reviewer's comment, we note that a detailed stability characterization of the derived cell lines will be published separately in an upcoming manuscript. However, we have included preliminary data on colony formation, DNA-content distribution using platinum-based compounds in the Supplementary Materials (S1).

3. The authors did not perform long-term stability studies of the new cells, such as passage-stability testing.

- Thank you for pointing that out. It should be noted that we do not maintain resistant cells with continuous platinum treatment, as platinum resistance remains stable and persists even after cryopreservation. However, if we observe a decline in resistance, we briefly re-expose the cells to platinum to restore their resistance. This approach ensures consistent resistance levels throughout our experiments. We have clarified this in the Materials and Methods section.

4. I'm wondering if the authors observed cross drug-resistance on HCT116 cspl-R or HCT116 oxpl-R? Can these cell lines resist other platinum drugs?

We appreciate the reviewer's insightful question regarding cross-resistance in our HCT116 cisplatin-resistant (HCT116 cispl-R) and oxaliplatin-resistant (HCT116 oxpl-R) cell lines. Indeed, we evaluated cross-resistance to other platinum-based drugs (e.g., oxaliplatin in HCT116 cispl-R and cisplatin in HCT116 oxpl-R).
According to our experimental data, cisplatin-resistant HCT116 cspl-R cells do not exhibit cross-resistance to oxaliplatin, a next-generation platinum drug (Fig S1D). However, they show slight cross-resistance to other DNA-damaging agents, including actinomycin D, etoposide, and adriamycin (doxorubicin). Notably, these cells remain sensitive to the topoisomerase I inhibitor irinotecan, the antimetabolite 5-fluorouracil, and histone deacetylase inhibitors such sodium butyrate (data not shown).
Oxaliplatin-resistant cells also demonstrate high resistance to cisplatin, at a level comparable to cisplatin-resistant cells, which allows considering them as a more universal model of generalized resistance to platinum drugs. Nevertheless, the overall spectrum of drugs to which HCT116 oxpl-R and HCT116 cspl-R cells exhibit cross-resistance largely overlaps. We have added this information in the manuscript (data not shown) and provided experimental data on cross-resistance to platinum drugs as supplementary material (Fig S1).

5. The language used occasionally feels a bit choppy, with ideas jumping abruptly from one paragraph to the next.

- We have refined the language and incorporated some logical transitions to make the manuscript easier to read.

To sum up our response, we would like to highlight that we have included in the manuscript a brief characterization of the resistant cells, while more comprehensive analysis including karyotyping, transcriptome profiling of oxaliplatin-resistant cells, and cross-resistance data will be published later this year in a separate article.

Response to reviewer 2

Thank you very much for your attention to our study and for your constructive comments. Your input has been valuable in making the article more comprehensive and of higher quality. Below, we will address your questions point by point:

1. The authors state: "To date, no dormancy models utilizing drug-resistant cells have been reported in the literature." (Lines 439–441)

While this claim may be valid, it would benefit from clearer positioning against existing models of dormancy. For example, the recent review by Kamat et al. (Molecular Cancer, 2025, Vol. 24) discusses a range of drug-tolerant and dormancy-related systems. The authors should acknowledge these works and better articulate the specific novelty of their approach.

- We appreciate the reviewer’s insightful comment and agree that our statement regarding the novelty of drug-resistant dormancy models could be more precisely contextualized within the existing literature. While we acknowledge the existing of drug-tolerant and dormancy-related systems, our claim specifically refers to the absence of in vitro dormancy models that incorporate drug-resistant cancer cells (e.g., those with defined resistance mutations or long-term resistance phenotypes) to study dormant cell reawakening. After an in-depth literature review, we managed to identify a few models based on resistant cells, and we have cited them in the discussion. To clarify this distinction, we revised the manuscript, referring to a review devoted to modeling cancer cell dormancy, as well as a number of original papers, to take into account broader models of dormancy and drug tolerance: “Drug-induced dormancy reported in literature covers various approaches [6]. Wu et al. induced dormancy in EGF-treated colorectal cancer cells (LoVo, HCT116) using 5-FU for 48 hours, resulting in slow-cycling/dormant cells with elevated stem cell markers (CD133, CD44, LGR5) and enhanced chemoresistance [33]. Li et al. modeled dormancy in breast or prostate cancer cells with high-dose Docetaxel or Doxorubicin, identifying dormant cells after 8-10 days with high p21/Waf1 and PKH26 retention, which resumed proliferation after 18-22 days and formed “recurrent” colonies [34]. Doxorubicin-resistant (DoxR) triple-negative breast cancer (TNBC) models (e.g., MDA-MB-231, BT-549) exhibit dormancy features such as G0/G1 arrest, upregulated p53/CHK2 signaling, and suppressed lipid metabolism. However, these models primarily focus on transient drug tolerance rather than stable resistance with defined genetic mutations, as in our system [35]. Guiro et al. studied dormancy in carboplatin-resistant breast cancer cells using 3D poly(ε-caprolactone) scaffolds, showing scaffold architecture influences dormancy kinetics [36]. Hangauer et al.

modeled minimal residual disease in HER2-amplified BT474 cells with lapatinib, producing quiescent survivors that regained proliferation post-drug withdrawal, suggesting non-mutational resistance [37]. In contrast, our model examines persistent resistance from prolonged chemotherapy, likely involving genetic alterations.

- Despite numerous models of drug-induced dormancy described to date, models specifically utilizing drug-resistant cancer cells remain scarce in the literature. Drug-resistant cell lines are typically established to investigate mechanisms of resistance, whereas dormancy is often studied either as a related phenomenon or by deriving dormant cells from drug-sensitive lines. In most dormancy-focused studies, drug-sensitive cells undergo short-term treatment, resulting in the simultaneous emergence of resistance and dormancy within the model. The key difference in our approach is the use of drug-resistant cells for modeling."

2. The use of FUCCI labeling is appropriate and informative. However, additional validation—such as Ki-67 immunostaining—would help more definitively distinguish true G0 quiescence from G1 arrest.

- We thank the reviewer for acknowledging the utility of FUCCI labeling in our study and for the suggestion to further validate distinctions between G0 quiescence and G1 arrest. As recommended, we have now performed additional molecular validation using qPCR analysis of ki-67 and key proliferative genes (e.g., aurka, cyclin A, cyclin B). These data have been incorporated into Figure 2D in the revised manuscript.
  We have included explanations of the results obtained in the main text of the manuscript:
  "qPCR analysis revealed that expression of proliferation markers such as Aurora kinase A (AURKA), MKI67, E2F1, and cyclins A and B significantly decreased 3-6 days after oxaliplatin treatment in both HCT116 oxpl-R and HCT116 cspl-R cell lines (Figs 2D, 3A). This decline coincided with peak mKO2/Cdt1 staining (marking G0/G1 phase) (Fig 2A). Expression level of these markers returns to baseline after repopulation (day 33). Conversely, CDK inhibitors p21/Waf1 and p27/Kip1 accumulate during the quiescent state (days 1–9), followed by downregulation upon cell cycle re-entry (Fig 3B,C). These molecular data provide independent evidence supporting our initial FUCCI-based conclusions regarding cell cycle arrest and recovery."
  Representative Ki-67 immunostaining (or Western blot) was considered. However, qPCR enabled quantitative, multi-gene profiling of cells at different stages of recurrence, aligning best with our mechanistic focus on transcriptomic regulation of quiescence.
  We agree that combining dynamic reporters (FUCCI) with molecular markers such as Ki-67 offers a more comprehensive view of cell-cycle states, and believe these additions significantly enhance our findings.

3. The manuscript reports dynamic changes in dormancy-related markers (e.g., FoxO1/3, LC3, Beclin, Twist1), but does not explore their causality. Are these markers drivers of dormancy, or merely consequences of treatment-induced stress?

- The observational nature of our data limits causal inferences. FoxO1/3, LC3, Beclin-1, and Twist1 are implicated in dormancy, but their driver or consequence status requires further investigation. These findings highlight their potential as biomarkers and therapeutic targets. Further genetic/pharmacological loss-of-function experiments are needed to determine the role of core pathways or protein in regulation of dormancy entry or maintenance. We intend to present the results of modulating key pathways (p38 MAPK, autophagy, ROS detoxification, FOXO1/3) in dormant cells in a separate publication. These findings may provide new insights into the mechanisms of maintaining the dormant state of cells, which may be useful for developing the therapeutic strategies to prevent relapse, such as reactivating dormant cells for subsequent cytotoxic therapy, maintaining dormancy, or selectively eliminating dormant populations. We are currently conducting experiments to investigate the role of FoxO transcription factors and autophagy modulation in maintaining dormancy using the cell model presented in this Article. This Article is conceptually intended by us as a detailed description of the model, followed by a series of papers presenting experimental results obtained based on the described model, with this article serving as the foundational groundwork.

4. The manuscript requires thorough language editing for grammar, clarity, and consistency.

- We have refined the language and incorporated logical transitions to make the manuscript easier to read.

Response to reviewer 3

We appreciate the reviewer's careful reading of our work and the valuable feedback provided. Your suggestions have significantly strengthened the manuscript and enhanced its overall quality and clarity. Below, we will address your questions point by point:

Major Comments

1. Inconsistent Use of Resistant Cell Lines: Although both cisplatin- and oxaliplatin-resistant cell lines are described, the authors alternate between them across experiments despite noting that they exhibit different repopulation kinetics (lines 453–458). This inconsistency may raise concerns about selective data presentation. To enhance the model's generalizability and scientific transparency, key findings should be validated in both cell lines.

- We appreciate the reviewer's attention to these details and regret any confusion caused by the data presentation. The discrepancies regarding the cell lines arose from our transition to an oxaliplatin-resistant subline, which was selected for its broader relevance due to cross-resistance across platinum-based therapies. While this subline shares key characteristics with the cisplatin-resistant cells, its enhanced universality better aligns with our study's goals. To address these concerns directly,

we have performed additional validation experiments, including immunoblotting (and qPCR analysis) of key dormancy markers in the oxaliplatin-resistant cells. We expanded the functional characterization of cisplatin-resistant cells with LysoTracker (autophagy) and DCF (ROS) assays to confirm conserved dormancy phenotypes, updating Figures 5 and 7 (and Supplementary Materials) with new experimental data to ensure full transparency.

2. Lack of Functional Validation of Dormancy Pathways: While dormancy-associated features are well-characterized, functional testing of core pathways (e.g., p38 MAPK, autophagy, ROS detoxification) is lacking. Perturbing these pathways using small-molecule inhibitors (e.g., SB203580 for p38, 3-MA for autophagy) or gene knockdown approaches (e.g., FOXO3 or Beclin1) would significantly strengthen the mechanistic conclusions.

- We are grateful to the reviewer for this reasonable remark on the development of research directions using the dormancy/tumor recurrence model presented in this Article. We intend to present the results of modulating key pathways (p38 MAPK, autophagy, ROS detoxification, FOXO1/3) in dormant cells in a separate publication. These findings may provide new insights into the mechanisms of maintaining the dormant state of cells, which may be useful for developing the therapeutic strategies to prevent relapse, such as reactivating dormant cells for subsequent cytotoxic therapy, maintaining dormancy, or selectively eliminating dormant populations. We are currently conducting experiments to investigate the role of FoxO transcription factors and autophagy modulation in maintaining dormancy using the cell model presented in this Article. This Article is conceptually intended by us as a detailed description of the model, followed by a series of papers presenting experimental results obtained based on the described model, with this article serving as the foundational groundwork.

3. Inconsistencies in EMT Marker Expression: The simultaneous upregulation of E-cadherin and N-cadherin is acknowledged but insufficiently addressed. This contradiction could reflect epithelial-mesenchymal plasticity or cellular heterogeneity. Follow-up experiments such as single-cell transcriptomics or immunofluorescence staining are recommended to clarify the phenotype

- We appreciate the reviewer’s insightful observation regarding the simultaneous upregulation of E-cadherin and N-cadherin in our study. We acknowledge that this apparent contradiction could indeed reflect epithelial-mesenchymal plasticity or cellular heterogeneity within the examined population, as suggested. Clarifying this issue may be an interesting direction for our future work. Thank you for this valuable suggestion, which will guide our further research on this topic. Currently, we analyse transcriptomic data of our oxpl-R cells versus sensitive HCT116 cells. With these data, we can perform a comprehensive study on EMT under oxaliplatin in these cells. While these experiments were beyond the scope of the current manuscript, we have now included a more detailed discussion of this point with additional literature data. We hope this clarification strengthens the interpretation of our findings.
  In Text: “Our qPCR data show increased EMT marker expression in the model. (Fig 6A). We showed that resistant cells, after platinum treatment, upregulated the expression of TWIST1, a factor inducing EMT, which allows us to assume that

dormant cells in our model undergo EMT. However, epithelial marker E-cadherin, which usually shows opposite dynamics to N-cadherin and is known to be downregulated during EMT, is also upregulated in our model. This unexpected E-cadherin activation may reflect population heterogeneity or incomplete EMT in dormant cells. It's reported that E-cadherin accumulation in quiescent cells can promote proliferation resumption via mesenchymal-epithelial transition (MET) [20]. There is a concept of epithelial-mesenchymal plasticity (EMP), stating that sometimes cells may not require complete EMT but rather fluid transitions between hybrid epithelial-mesenchymal phenotypes [50,51]. This hybrid state is highly plastic and can switch between different EMT states, enhancing the cancer's ability to invade, metastasize, and resist therapy [26,52].

According to Ruth et al., in dormant residual cells, N-cadherin activation is not always associated with E-cadherin suppression. In particular, in Her2-driven tumors, N-cadherin is more active in dormant cells compared to proliferating tumor cells, while E-cadherin expression remains unchanged. By contrast, in Wnt-driven tumors, E-cadherin levels are elevated in dormant cells without N-cadherin activation [53]. It has also been reported that E-cadherin expression can coexist with the EMT-promoting factor Slug [54].

While our current data do not allow us to identify the exact mechanisms, the findings highlight the complexity of EMT regulation and suggest governing E-cadherin/N-cadherin balance may be critical for understanding their roles in dormant state regulation and therapeutic resistance."

4. Discussion of Model Limitations: The current discussion does not adequately address the model's limitations, particularly the absence of tumor microenvironmental components (immune, stromal, and hypoxic factors), which are critical regulators of dormancy in vivo. This gap should be more thoroughly considered in the context of the model's translational relevance.

- Thank you for pointing this out. We have reworked the discussion to put more emphasis on the model's limitations:

  In Text: "While we cannot directly address the other two limitations related to immune component and recurrence timing in this in vitro system, the model's reproducibility and usability of our model make it a promising tool for primary exploratory research. Moreover, the presented model has the potential for further upgrade and refinement, such as incorporating soluble factors into the culture medium or integrating it into direct or indirect co-culture systems with immune cells. Future work will explore adapting this model to in vivo conditions, which could help partially overcome the aforementioned limitations by better recapitulating the tumor microenvironment and immune interactions. This advancement would enhance the physiological relevance of the model and expand its applicability for studying tumor dormancy and drug resistance."

5. Data Presentation and Clarity: Several figures and legends are overly dense or lack clarity. Normalization methods, time points, and statistical annotations (including p-values and sample size) should be consistently reported. Graphs would benefit from cleaner labeling and clearer presentation of fold-changes relative to key states (e.g., peak quiescence or baseline proliferation).

- We improved the quality of the illustrative materials, made the figure legends clearer and more explicit, clearly indicated the sample sizes and the metrics used to represent error bars in the figure captions, and specified the P value thresholds in the Materials and Methods section. As you recommended, we marked peak quiescence and baseline proliferation points in schemes.

6. Figure Legends and Abbreviations: Figure legends should be more comprehensive and include all abbreviations and treatment details referenced in the figure. This will improve interpretability without needing to refer back to the main text.

- Thank you for this remark, we expanded the figure legends with more details.

7. Discrepancy Between Imaging and Graphs in Figure 2: There is a mismatch between the days shown in fluorescent images (days 2 and 4) and the time points in the graph (days 3, 4, 5). This should be clarified or aligned for consistency.

- There was indeed an error in the label, which we have now corrected to ensure consistency throughout the figure.

8. Drug Treatment Justification: In Materials and Methods (line 92), the rationale for selecting 25–50 μM cisplatin and 50–150 μM oxaliplatin with exposure times of 6/24 hours should be explicitly stated or supported by literature references.

- The drug concentrations (25–50 μM cisplatin, 50–150 μM oxaliplatin) and exposure times (6/24 hours) were selected based on empirical optimization relative to the IC50 values of the resistant cell lines. To ensure relevance to the drug-resistant phenotype, we used concentrations proportional to those applied during the development of resistance (see Materials and Methods Section). We clarified this in text: “To choose the optimal parameters of the model, HCT116 oxpl-R cells were treated with 50 μM or 150 μM oxaliplatin, while HCT116 cispl-R cells received 25 μM or 75 μM cisplatin. These concentrations are proportional to the known IC50 values and include the working concentrations used for drug resistance development [31, 32].”

9. Drug Concentration Justification (Line 276): Drug concentrations for viability testing (e.g., etoposide, irinotecan, 5-FU) are listed without justification. Appropriate references or pilot data should be provided to validate the chosen doses.

- The drug concentrations used for viability tests (etoposide, irinotecan, 5-FU) were selected based on their established IC50 values for the parental, platinum-sensitive HCT116 cell line, as determined in our preliminary dose-response experiments (data not shown). By using doses near the IC50 of sensitive cells, we were able to

effectively compare drug responses between parental and resistant cell populations. We have now clarified this rationale in the corresponding section of results.

10. Inconsistency in Figure 5 Treatments: The rationale for using cisplatin in Figure 5a and oxaliplatin in Figures 5b/c is unclear. Uniform treatment or explanation of the differential use is necessary.

- We supplemented Figures 5 and 8 with results obtained from HCT116 cspl-R cells, so now both lines are presented: cisplatin-treated HCT116 cspl-R cells and oxaliplatin-treated HCT116 oxpl-R cells.

11. Unjustified Reagent Choice (Line 225): The detailed 0–33-day time-course is performed using cisplatin, not oxaliplatin. A justification for this choice is needed, or results should be presented for both agents to support the model's robustness.

- We treat each line with a corresponding platinum drug: cisplatin for modeling recurrence in cisplatin-resistant cells (HCT116 cspl-R) and oxaliplatin for modeling recurrence in oxaliplatin-resistant cells (HCT116 oxpl-R). To avoid confusion, we added this note to the Materials and Methods section.

12. Statistical Reporting: The authors report SEM instead of SD without justification. Given the small number of replicates (n=3), reporting SD may more accurately reflect biological variability unless multiple independent experiments were conducted.

- We appreciate the reviewer's concern regarding the reporting of SEM (Standard Error of the Mean) instead of SD (Standard Deviation).
  We used SEM to estimate the accuracy of the average over several experiments. Due to the fact that SEM is low, we can conclude that the sample we examined accurately displays the general population and that the sample mean does not significantly differ from the true average of the general population (SEM shows how much does the sample average deviate from the true average of the general population). To address this, we have now included both SD and SEM in the revised manuscript and clarified in the Methods section that the replicates represent biologically independent experiments. This dual reporting ensures transparency while allowing readers to assess both variability (SD) and estimation precision (SEM).
  Modified text in Materials and methods section:
  "The plots represent the mean of 3 independent experiments ± standard error of the mean (SEM) or 3 independent samples ± standard deviation (SD), the metric applied is included in the labels for each figure."
  We hope this adjustment meets the reviewer's expectations. Thank you for the constructive feedback.

13. Misinterpretation of FUCCI Data (Line 224): The claim that cell cycle distribution "returns to its initial state" by day 13 is not fully supported by the data. mKO2-Cdt1-positive cells still represent ~35% versus ~10% at day 0. This needs rephrasing or additional justification.

- Indeed, in accordance with Figure 2, as well as our other data, only some individual cells return to proliferation 13 days after drug treatment. Whereas a complete return of cell cycle distribution to the initial state occurs only after 25-33 days. We have

rewritten the paragraph, changing the statement about the cell cycle distribution returning to its initial state by day 13 as: “Recurrence modeling on FUCCI-labeled cells reveals a pronounced accumulation of red fluorescent (mKO2-Cdt1) upon entering the quiescent state, suggesting transition to G0/G1 phase (Figure 2b,c). Cells in G0/G1 are prevalent until day 7-9. After that, individual cells switch to early S-phase, which is marked by colocalized green and red signals, visible as yellow, indicating the onset of cell repopulation. Concurrently, a reduction in the total red signal reflects progressive exit from G0/G1. The cell cycle distribution tends to return to its initial value.”

The revised text maintains the key finding of cell cycle re-entry while properly contextualizing the extended timeline for complete distribution normalization.

14. Missing p27 mRNA Data: In Figure 3, the accumulation of p27 is shown by western blot, but not confirmed at the transcript level. Including qPCR results would provide a more complete assessment of cell cycle regulation.

- We appreciate the reviewer’s suggestion regarding p27 mRNA analysis. Unfortunately, at this stage, we are unable to perform qPCR with primers for p27. While qPCR data would indeed provide additional insight into transcriptional regulation, our current study focuses on the functional role of p27 protein accumulation in drug-induced quiescence, as demonstrated by Western blot (Fig. 3C). Since p27 is primarily regulated post-translationally (e.g., via proteasomal degradation), its protein levels are most relevant to cell cycle arrest rather than transcript abundance.

15. Overstated Persistence of Beclin (Line 304): The text claims Beclin accumulation persists through repopulation, but Figure 5 suggests otherwise. This conclusion should be revised or supported with additional quantification.

- We sincerely thank the reviewer for this careful observation regarding the interpretation of Beclin-1 dynamics. Upon re-examination of our data, we agree that the original statement overstated Beclin-1 persistence during repopulation and have revised our conclusions accordingly.

  Original: "Beclin accumulation persists through repopulation..."

  Revised: "Our recurrence model demonstrates biphasic regulation of autophagy. While Beclin-1 levels remain elevated during dormancy (Days 4–14), quantitative analysis reveals a significant decrease during repopulation (Day 21) compared to peak dormancy levels (Figure 5C; Supplementary 3), suggesting that partial autophagy downregulation accompanies tumor regrowth."

  According to our data, Beclin-1 expression increases during the transition to dormancy and subsequently declines upon tumor repopulation.

  We appreciate this opportunity to improve the accuracy of our reporting and believe these revisions provide a more precise representation of the Beclin-1 dynamics while maintaining the key finding of dormancy-associated autophagy activation. The modified text and it now aligns with the quantitative evidence in Figure 5 while acknowledging the complexity of autophagy regulation during cancer recurrence.

16. Need for Quantification of Immunoblots (Line 307): The claim that survivin accumulation corresponds with LC3/Beclin decline is not visually convincing. Quantitative densitometry should be provided to support this interpretation.

- We appreciate the reviewer's careful evaluation of our immunoblot data and agree that quantitative analysis is important to interpretation of the relationship between survivin accumulation and LC3/Beclin-1 decline. We have performed quantitative densitometry (ImageJ) on all relevant immunoblots, normalizing intensity levels to their respective loading controls (α-Tubulin).
  We acknowledge that in the initial submission, while densitometry values were provided beneath the blots, the small font size may have compromised readability. In the revised version of the manuscript, we increased the font size of all densitometry values, changed text color from gray to high-contrast black bold style for improved visibility. We updated Figure legend as "Quantification values shown beneath each lane represent band intensity normalized to loading control and relative to day 0 (set as 1.0)."
  We understand that immunoblots alone cannot establish causality; future studies with survivin knockdown/overexpression will further validate this mechanism.

Minor Comments

• *A brief comparison with other drug-induced dormancy models (e.g., breast or lung cancer models treated with doxorubicin or docetaxel) would help contextualize the significance of this work within the field.*

- We appreciate the reviewer's suggestion to contextualize our drug-resistant dormancy model within the broader landscape of drug-induced dormancy studies. In the Discussion section of manuscript, we briefly compare our model with other drug-induced dormancy models, highlighting the novelty of our approach.

• *Minor typographical and formatting issues (e.g., inconsistent italicization of gene names, figure label placement) should be corrected throughout the manuscript.*

- We checked and corrected the spelling of genes consistently throughout the text and in the figure captions according to HUGO Gene Nomenclature (italicised, with all letters in uppercase).

• *In Figure 1b, visual cues such as arrows should be added to highlight key features (e.g., vacuolization, multinucleation) for clarity.*

- In Figure 1b we have added arrows to make it easier to focus on certain details (vacuolization, multinucleation)

We hope these revisions address the reviewer's concern and provide robust support for our conclusions. We appreciate your insightful comments that contributed to improving the quality of our manuscript.

Response to reviewer 4

p. 4, lines 74: The term "present" suggests that there should be a "presented."

- We sincerely appreciate the reviewer's positive evaluation of our manuscript. We have made a corresponding change in the text.

Please provide original full resolution images of Fig 1b

- We have included full uncropped images of Fig 1b in the Supplementary Materials (S2) and in the Supporting information.

We believe these revisions have strengthened our manuscript and hope that the improvements meet the expectations of both you and the reviewers. We remain open to any further suggestions and would be happy to address additional requests promptly.

Thank you very much for your consideration.

Sincerely,
Alisa Morshneva
On behalf of the co-authors

Corresponding Author:
Alisa Morshneva
Email: 1195alisa@gmail.com
Department of Molecular and Cell Interaction, Group of Molecular Basis of Carcinogenesis (Maria Igotti's Research Group),
Institute of Cytology, Russian Academy of Sciences, 194064, Tikhoretsky Prospekt, 4, St. Petersburg, Russia.
