# Supplementary material for: An in vitro tumor recurrence model based on platinum-resistant colon cancer cells as a research tool for studying cancer cell dormancy
Source: PLoS One. 2025 Oct 8;20(10):e0333671. doi: 10.1371/journal.pone.0333671 (PMC12507233; doi:10.1371/journal.pone.0333671)
Supplement: Attachment — Submitted filename: Responce to Reviewers R2.pdf [file pone.0333671.s007.pdf]

REBUTTAL LETTER

Dear Li Yang,

We would like to thank you and the reviewers for the prompt revision and for the high appreciation of our work. We are glad that the reviewers are satisfied with our responses. However, there appears to be a misunderstanding, as the new comments from Reviewer 4 have already been addressed and corrected in response to requests from the other three reviewers.
In any case we provide copies of our previous responses below to address all comments from Reviewer 4.
Therefore, we have not made any additional changes to the manuscript text in this round of revision.

Response to reviewer 4

When applied to platinum medicines, the scientists did not conduct short-term stability investigations of the new cells, such as colony formation and apoptosis studies.

- Regarding the reviewer's comment, we note that a detailed stability characterization of the derived cell lines will be published separately in an upcoming manuscript. However, we have included preliminary data on colony formation, DNA-content distribution using platinum-based compounds in the Supplementary Materials (S1).

The novel cells were not subjected to long-term stability experiments by the authors, including passage-stability testing.

- Thank you for pointing that out. It should be noted that we do not maintain resistant cells with continuous platinum treatment, as platinum resistance remains stable and persists even after cryopreservation. However, if we observe a decline in resistance, we briefly re-expose the cells to platinum to restore their resistance. This approach ensures consistent resistance levels throughout our experiments. We have clarified this in the Materials and Methods section.

The figures must be clearly and meaningfully prepared at high quality. Both by themselves and with the typography, the manuscript's images lacked visual appeal. It was suggested that they be changed and modified.

- We improved the quality of the illustrative materials, made the figure legends clearer and more explicit

Occasionally, the wording feels a little chaotic, with concepts leaping from one paragraph to the next without explanation.

- We have refined the language and incorporated some logical transitions to make the manuscript easier to read.

The article does not investigate the causal relationship between the dynamic changes in dormancy-related markers (such as FoxO1/3, LC3, Beclin, and Twist1) that are reported in

the study. Do these indicators indicate reasons for dormancy, or are they just effects of stress brought on by treatment?

- The observational nature of our data limits causal inferences. FoxO1/3, LC3, Beclin-1, and Twist1 are implicated in dormancy, but their driver or consequence status requires further investigation. These findings highlight their potential as biomarkers and therapeutic targets. Further genetic/pharmacological loss-of-function experiments are needed to determine the role of core pathways or protein in regulation of dormancy entry or maintenance. We intend to present the results of modulating key pathways (p38 MAPK, autophagy, ROS detoxification, FOXO1/3) in dormant cells in a separate publication. These findings may provide new insights into the mechanisms of maintaining the dormant state of cells, which may be useful for developing the therapeutic strategies to prevent relapse, such as reactivating dormant cells for subsequent cytotoxic therapy, maintaining dormancy, or selectively eliminating dormant populations. We are currently conducting experiments to investigate the role of FoxO transcription factors and autophagy modulation in maintaining dormancy using the cell model presented in this Article. This Article is conceptually intended by us as a detailed description of the model, followed by a series of papers presenting experimental results obtained based on the described model, with this article serving as the foundational groundwork.

Absence of Functional Validation of Dormancy Pathways: Although characteristics linked to dormancy have been thoroughly described, there is a dearth of functional testing for key pathways (such as p38 MAPK, autophagy, and ROS detoxification). The mechanistic results would be much strengthened if these pathways were perturbed using gene knockdown techniques (e.g., FOXO3 or Beclin1) or smallmolecule inhibitors (e.g., SB203580 for p38, 3-MA for autophagy).

- We are grateful to the reviewer for this reasonable remark on the development of research directions using the dormancy/tumor recurrence model presented in this Article. We intend to present the results of modulating key pathways (p38 MAPK, autophagy, ROS detoxification, FOXO1/3) in dormant cells in a separate publication. These findings may provide new insights into the mechanisms of maintaining the dormant state of cells, which may be useful for developing the therapeutic strategies to prevent relapse, such as reactivating dormant cells for subsequent cytotoxic therapy, maintaining dormancy, or selectively eliminating dormant populations. We are currently conducting experiments to investigate the role of FoxO transcription factors and autophagy modulation in maintaining dormancy using the cell model presented in this Article. This Article is conceptually intended by us as a detailed description of the model, followed by a series of papers presenting experimental results obtained based on the described model, with this article serving as the foundational groundwork.

Limitation is missing in the study

- In Text: “While we cannot directly address the other two limitations related to immune component and recurrence timing in this in vitro system, the model's reproducibility and usability of our model make it a promising tool for primary exploratory research. Moreover, the presented model has the potential for further upgrade and refinement, such as incorporating soluble factors into the culture medium or integrating it into direct or indirect co-culture systems with immune cells. Future work will explore

adapting this model to in vivo conditions, which could help partially overcome the aforementioned limitations by better recapitulating the tumor microenvironment and immune interactions. This advancement would enhance the physiological relevance of the model and expand its applicability for studying tumor dormancy and drug resistance."

Disparities in EMT Marker Expression: Ecadherin and N-cadherin's concurrent overexpression is recognized but not adequately addressed

- We appreciate the reviewer's insightful observation regarding the simultaneous upregulation of E-cadherin and N-cadherin in our study. We acknowledge that this apparent contradiction could indeed reflect epithelial-mesenchymal plasticity or cellular heterogeneity within the examined population, as suggested. Clarifying this issue may be an interesting direction for our future work. Thank you for this valuable suggestion, which will guide our further research on this topic. Currently, we analyse transcriptomic data of our oxpl-R cells versus sensitive HCT116 cells. With these data, we can perform a comprehensive study on EMT under oxaliplatin in these cells. While these experiments were beyond the scope of the current manuscript, we have now included a more detailed discussion of this point with additional literature data. We hope this clarification strengthens the interpretation of our findings.
  In Text: "Our qPCR data show increased EMT marker expression in the model. (Fig 6A). We showed that resistant cells, after platinum treatment, upregulated the expression of TWIST1, a factor inducing EMT, which allows us to assume that dormant cells in our model undergo EMT. However, epithelial marker E-cadherin, which usually shows opposite dynamics to N-cadherin and is known to be downregulated during EMT, is also upregulated in our model. This unexpected E-cadherin activation may reflect population heterogeneity or incomplete EMT in dormant cells. It's reported that E-cadherin accumulation in quiescent cells can promote proliferation resumption via mesenchymal-epithelial transition (MET) [20]. There is a concept of epithelial-mesenchymal plasticity (EMP), stating that sometimes cells may not require complete EMT but rather fluid transitions between hybrid epithelial-mesenchymal phenotypes [50,51]. This hybrid state is highly plastic and can switch between different EMT states, enhancing the cancer's ability to invade, metastasize, and resist therapy [26,52].
  According to Ruth et al., in dormant residual cells, N-cadherin activation is not always associated with E-cadherin suppression. In particular, in Her2-driven tumors, N-cadherin is more active in dormant cells compared to proliferating tumor cells, while E-cadherin expression remains unchanged. By contrast, in Wnt-driven tumors, E-cadherin levels are elevated in dormant cells without N-cadherin activation [53]. It has also been reported that E-cadherin expression can coexist with the EMT-promoting factor Slug [54].
  While our current data do not allow us to identify the exact mechanisms, the findings highlight the complexity of EMT regulation and suggest governing E-cadherin/N-cadherin balance may be critical for understanding their roles in dormant state regulation and therapeutic resistance."

We remain open to any further suggestions and would be happy to address additional requests promptly.

Thank you very much for your consideration.

Sincerely,
Alisa Morshneva
On behalf of the co-authors

Corresponding Author:
Alisa Morshneva
Email: 1195alisa@gmail.com
Department of Molecular and Cell Interaction, Group of Molecular Basis of Carcinogenesis (Maria Igotti's Research Group),
Institute of Cytology, Russian Academy of Sciences, 194064, Tikhoretsky Prospekt, 4, St. Petersburg, Russia.
